# Regioselective Hydration of Terpenes with Cofactor‐Independent Carotenoid 1,2‐Hydratase

**DOI:** 10.1002/anie.202505942

**Published:** 2025-07-17

**Authors:** Philip Horz, Natalie Härterich, Andreas Schneider, Nicolas D. Travnicek, Bettina M. Nestl, Ursula Kahler, Bernhard Hauer

**Affiliations:** ^1^ Department of Technical Biochemistry Institute of Biochemistry and Technical Biochemistry Department of Technical Biochemistry University of Stuttgart Allmandring 31 70569 Stuttgart Germany; ^2^ Innophore GmbH Am Eisernen Tor 3 8010 Graz Austria

**Keywords:** Biocatalysis, Hydratase, Terpene, Terpenoids, Water addition

## Abstract

Terminally hydrated terpenes are highly sought‐after compounds in the flavor and fragrance industries. However, their selective synthesis remains a considerable challenge in catalysis. Regioselective hydration of non‐activated C─C double bonds is typically hindered by poor selectivity and low atom efficiency in conventional methods. In this study, we harness the underexplored potential of the acyclic carotenoid 1,2‐hydratase from *Rubrivivax gelatinosus* IL144, employing it as a whole‐cell biocatalyst for cofactor‐independent terminal hydration of a diverse range of terpenes. This enzyme demonstrates exceptional activity across more than 20 C_12_─C_20_ terpenes and shows notable tolerance to various functional groups, establishing it as a valuable tool for sustainable organic synthesis. We emphasize the critical influence of expression system choice in maximizing enzymatic performance, enabling high‐yield transformations on the gram scale. Through a combination of homology modeling, consensus analysis, and targeted mutagenesis, essential residues involved in catalytic activity were identified. Notably, enhanced catalytic efficiency was only achievable through the epistatic effect of three specific mutations. These findings highlight the biocatalytic potential of acyclic carotenoid hydratase, offering a green and efficient route to the production of valuable tertiary alcohols.

## Graphical abstract

This study reveals a highly regioselective and cofactor‐independent enzymatic approach to terpene and terpenoid hydration using a carotenoid 1,2‐hydratase from Rubrivivax gelatinosus. Beyond detailing reaction optimization strategies, an extensive substrate scope, and scale‐up potential, it explores mutagenesis and structural modeling to identify key catalytic residues and improve enzymatic activity.

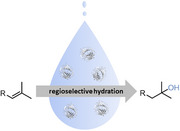

Adding water selectively to common olefins like terpenes is a promising strategy for producing valuable fragrances, such as lily of the valley and pharmaceutical molecules, like α‐terpineol (please see Figure S1 for examples).^[^ ^1^ ^]^ While the simple addition of water to a C─C double bond appears atom‐economical, its practical implementation poses several challenges (Figure 1): conventional chemical methods often require harsh conditions, such as high temperatures, pressures, strong acids or transition‐metal catalysts^[^ ^2^, ^3^, ^4^, ^5^, ^6^, ^7^ ^]^ which result in poor atom economy and unselective hydration. These limitations become more pronounced with terpenes containing multiple double bonds, necessitating complex multi‐step syntheses. (Figure S1).^[^ ^8^, ^9^, ^10^, ^11^ ^]^ The development of catalysts capable of achieving regioselective and cofactor‐independent hydration of non‐activated double bonds under mild conditions has thus become a critical focus in industrial and academic catalysis research. Nature has evolved a diverse set of hydratases that efficiently catalyze the addition of water to unactivated C─C double bonds via carbocation chemistry:^[^ ^12^, ^13^, ^14^, ^15^, ^16^ ^]^ Fatty acid hydratases (FAHs) have been used to hydrate various fatty acid derivatives,^[^ ^17^ ^]^ small alkenes^[^ ^18^, ^19^ ^]^ and styrenes^[^ ^20^ ^]^ with excellent stereocontrol (*ee* > 99%). However, these enzymes rely on oxygen‐sensitive flavin cofactors, which limits their practical application. For terminal terpene hydration, the kievitone hydratase has emerged as a potential candidate but is currently restricted to bulky prenylated isoflavonoids. (Figure S2).^[^ ^21^, ^22^ ^]^ Carotenoid 1,2‐hydratases (CrtCs), on the other hand, naturally catalyze cofactor‐independent terminal hydration of lycopene **1,** a molecule with 13 C─C double bonds (Figure S3).^[^ ^23^ ^]^ Despite previously reported low activity and narrow substrate scope, we were intrigued by their potential for broader application.^[^ ^24^, ^25^ ^]^

**Figure 1:**
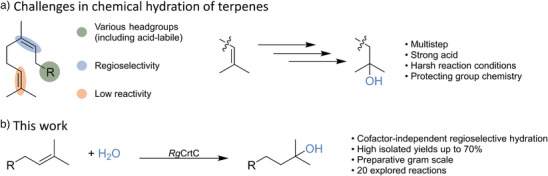
Challenges and synthesis routes for regioselective hydration of unactivated olefins. a) Molecules with multiple C─C double bonds are particularly challenging for the regioselective addition of water, especially terminal unactivated double bonds due to their low reactivity. Chemical hydration is used for the production of molecules of industrial interest, such as valuable flavors, fragrances and pharmaceutical molecules, by multistep chemical protection group synthesis under harsh reaction conditions. b) Enzymatic regioselective addition of water to unactivated olefins by a carotenoid 1,2‐ hydratase.

Here, we report on employing the highly active natural acyclic CrtC from *Rubrivivax gelatinosus* IL144 (*Rg*CrtC IL144) for the regioselective and cofactor‐independent terminal hydration of more than twenty terpenes (C_12_─C_20_). The biocatalyst exhibits high functional‐group tolerance, and allows for a simple preparation of tertiary alcohols on a preparative scale (Figure 1b). By combining mutagenesis and computational analysis, crucial positions for catalysis and substrate coordination were identified, and a strategy for the mutagenesis of these evolutionarily conserved enzymes was proposed.

To streamline the use of the regioselective catalyst, we adopted a whole‐cell setup, as previous studies have shown that membrane‐bound enzymes benefit from the native cell environment.^[^ ^26^, ^27^ ^]^ We successfully overexpressed the *Rg*CrtC IL144 in *E. coli* ITB94 as confirmed by SDS‐PAGE. Initial in vitro activity was validated using **1**, but due to its high hydrophobicity and membrane impermeability, terminal hydration to 1‐HO‐lycopene or 1,1′‐(HO)₂‐lycopene could not be achieved. (Figure S3). To overcome these limitations, we used a shorter analog C_18_ farnesylacetone **2** featuring a functional keto head group. Using 100 mg_cww_ mL^−1^ in 50 mM potassium phosphate (KPi) buffer (pH 7.0) with 1 mM substrate, we achieved 35% product formation of the terminally hydrated compound hydroxyfarnesylacetone **3** with complete regioselectivity. The product's structure was confirmed via isolation and NMR analysis (see substrate scope section for further details). This reaction then served as a model to optimize expression and catalytic parameters. (Figure S5). Compared to the previously described *Rg*CrtC S1,^[^ ^24^ ^]^ our naturally‐occurring enzyme variant *Rg*CrtC IL144 contains 17 globally distributed changes in amino acids, with minimal impact on enzymatic performance (Figure S4). A striking 50‐fold increase in product formation was observed using our expression system (pDHE1650 vector in ITB94 strain) versus the literature system (pET22b(+) vector in BL21(DE3)). We attribute this to tighter regulation and low‐copy plasmid maintenance, which may promote proper enzyme folding and activity. (35% versus 0.6%; Figure S5).^[^ ^28^, ^29^ ^]^ We next optimized reaction conditions, evaluating variables such as pH, temperature, 2‐hydroxypropyl‐β‐cyclodextrin (2HPCD) concentration, cofactor addition, and the use of lyophilized whole cells (Table 1 and Figures S6–S11). Ultimately, product yield was improved by 1.4‐fold to reach 50%, establishing a robust standard system: 100 mg_cww_ mL^−1^, 50 mM KPi buffer (pH 7.0), 1 mM substrate, and 2HPCD.

**Table 1:** Reaction parameter optimization of the model reaction with 2 toward 3 using the wildtype and knock‐out variant (D268 mutated to alanine).

Entry	*Rg*CrtC formulation	Change in conditions	Conversion (%)
**1**	Whole cell^a)^	standard conditions	35.3 ± 3.8
**2**	Whole cell KO^a)^	–	–
**3**	Whole cell^a)^	lyophilized	25.8 ± 1.3
**4**	Whole cell^a)^	citrate buffer 50 mM pH 5.0	32.8 ± 2.7
**5**	Whole cell^a)^	KPi buffer 50 mM pH 8.0	31.9 ± 3.0
**6**	Semi purified^b)^	addition of 1 mM NADH and 0.1 mM FAD	35.9 ± 2.0
**7**	Semi purified^b)^	addition of 1 mM NADPH and 0.1 mM FAD	35.5 ± 0.5
**8**	Whole cell^a)^	addition of 1 mM 2HPCD	35.7 ± 2.0
**9**	Whole cell^a)^	addition of 5 mM 2HPCD	49.9 ± 1.0
**10**	Purified^c)^	–	17.7 ± 0.7

^a)^ Whole cell reactions were performed with 100 mg_cww_ mL^−1^ in 50 mM buffers pH = 5.0–8.0, 1 mM of substrate 2 in DMSO, for 24 h at 30 °C.

^b)^ Semi purified reactions were carried out with solubilized enzyme (from 100 mg_cww_ mL^−1^) in 50 mM KPi pH = 7.0 + cofactors, 1 mM of substrate 2 in DMSO, for 24 h at 30 °C.

^c)^ Reactions with purified enzyme were performed with 0,27 µM µL^−1^ Enzyme in 50 mM NaPi pH = 7.0, 1 mM of substrate 2 in DMSO, for 24 h at 30 °C. Measurement was conducted without internal standard and relative conversion is indicated. All conversions represent mean values of technical triplicates (*n* = 3).

After identifying the optimal reaction conditions, we commenced an extensive study of various head‐to‐tail‐fused terpenes featuring diverse functional head groups. Product formation was analyzed using GC‐FID (Figures S12–S15) and GC‐MS (Figures S16–S22), with negative controls (Empty Vector and Buffer) employed. Mass fragmentation patterns were thoroughly examined. It is well‐documented in literature that terminally hydrated prenyl units such as in laurinal, α‐terpineol **4**, and hydroxyfarnesol **5** exhibit a mass of 59 *m/z*. Furthermore, the hydrated products displayed a decrease in 69 *m/z*. A characteristic of the terminal prenyl moiety in the substrate, aligning with existing literature findings.^[^ ^30^, ^31^, ^32^, ^33^ ^]^

With favorable conversions for the model substrate **2**, shorter ketones were investigated first. The C_13_ terpenes geranylacetone **6** and its conjugated analog pseudoionone **7** were used as substrates producing, hydrated products hydroxygeranylacetone **8** and hydroxypseudoionone **9** with 31% and 48%, respectively. We also investigated alcohol substrates. While longer alcohols such as geranylgeraniol yielded only 4% conversion to its diol **10**, shorter alcohols such as bishomofarnesol and homofarnesol **11** were converted at high rates to **12** and **13** (50% and 78%, respectively). Mid‐chain alcohols (C_12_─C_15_) such as farnesol, geranylisopropanol and calmusol, were transformed to 17% of product **5**, 7% of the terminally hydrated hydroxygeranylisopropanol **14** and 2% of the diol hydroxycalmusol **15**. Product hydroxygeranyllinalool **16** was formed with 25% product formation from the vinyl alcohol geranyllinalool. Terpenes with a carboxylic acid head group were then used as substrates, yielding terminally hydrated homofarnesylic acid **17** with a high product formation of 57% from homofarnesylic acid. Acid labile ethers and esters such as geranyl butyl ether, geranyl ethyl ether, homofarnesylacetate, geranylacetate and geranylthioacetate resulted in the production of hydration products **18**, **19**, **20**, **21**, and **22** with product formations ranging from 3% to 35%. It is worth mentioning that for the esters homofarnesylacetate and geranylacetate partial ester cleavage was detected. Unfunctionalized and bulky terpenes such as α‐ and β‐farnesene yielded hydration products **23** and **24** at moderate levels (21% and 17%), whereas more structurally hindered terpenes like santalene and bergamotene showed only ≤1% conversion to the products **25** and **26**. Despite the variation in substrate structure and reactivity, all reactions demonstrated absolute regioselectivity (Figure 2a).

**Figure 2:**
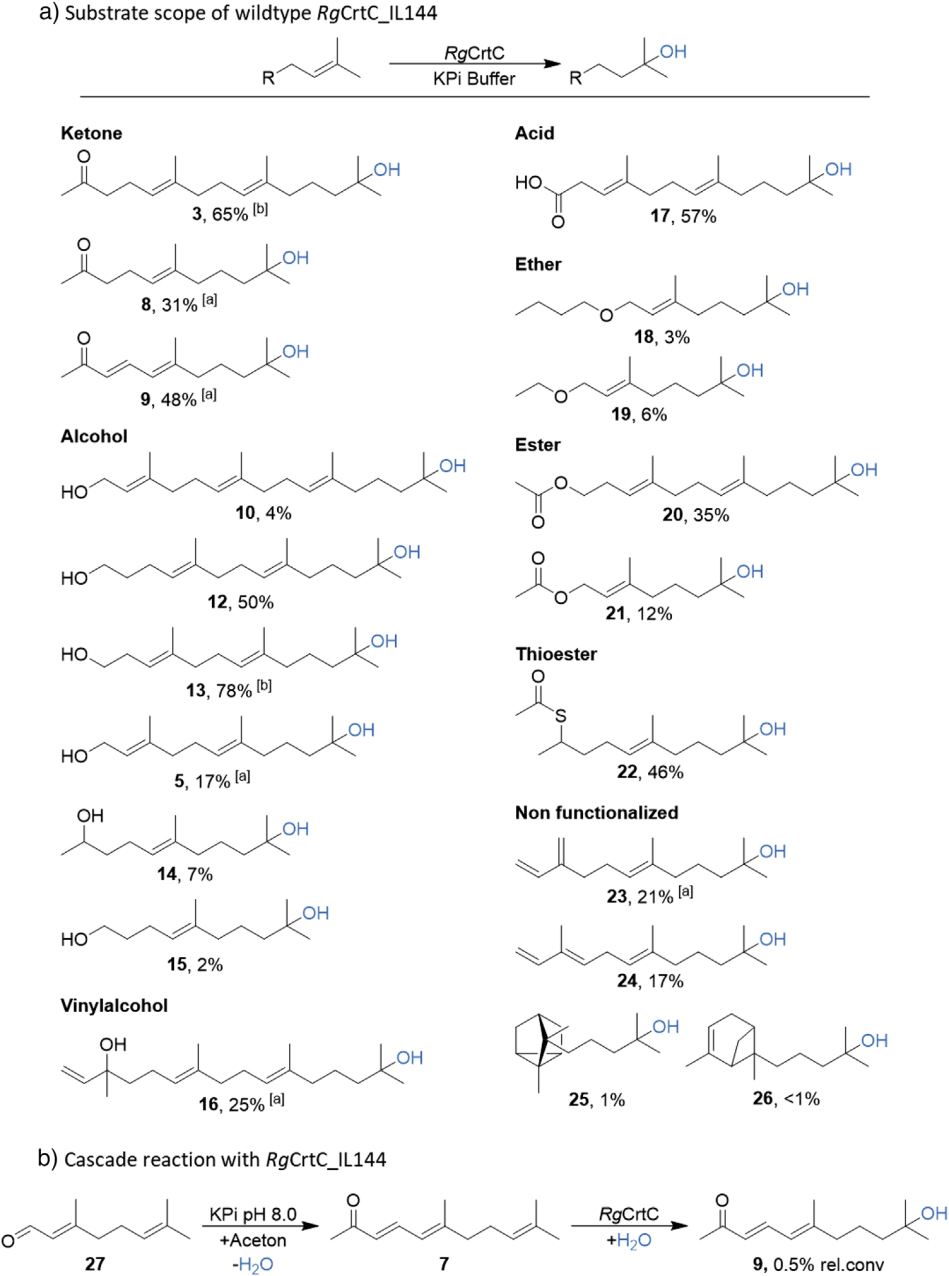
Terpene substrate scope of terminal regioselective hydration reactions and cascade application with wildtype *Rg*CrtC IL144. a) Schematic terminal hydration reaction. Standard reaction conditions: 100 mg_cww_/mL in KPi buffer pH = 7.0, 1 mM 2HPCD, 1 mM substrate in DMSO, 24 h at 30 °C. The yields represent mean values of technical triplicates (*n* = 3). ^[a]^ Scale‐Up reactions were performed with 40 mg_cww_/mL in KPi buffer pH = 7.0, 10 mM 2HPCD, 10 mM substrate for 5 d at 30 °C. Reactions were monitored via GC. ^[b]^ Scale‐Up reaction was performed with 40 mg_cww_/mL in KPi buffer pH = 7.0, 50 mM 2HPCD, 50 mM substrate for 5d at 30 °C b) One‐pot alkaline aldol condensation with subsequent hydration to product 9. Reaction conditions: 10 mg_cdw_ mL^−1^ in KPi buffer pH = 8.0, 5 mM 27 in acetone, 24 h at 30 °C.

Several aldehydes including, calmusal, citral **27** and (*R‐* or *S*)‐citronellal (Figure S23) were evaluated. No hydrated products were observed, likely due to their in vivo reduction.

Nevertheless, taking advantage of the enzyme's broad pH tolerance, we conceived a one‐pot formation of **9** starting from **27**. For this purpose, **27** was dissolved in acetone as a cosolvent and added to the lyophilized cell suspension prepared in basic KPi 50 mM pH  8.0. The basic conditions prompted the aldol condensation of **27** and acetone to form **7**, which was then enzymatically hydrated to yield **9** with 0.5% relative conversion (Figure 2b).

Further monoterpenes encompassing 6‐methyl‐5‐hepten‐2‐one, geraniol, (+)‐isopulegol, (−)‐isopulegol, linalool, myrcene, (+)‐β‐citronellen, (−)‐β‐citronellen, (*R*)‐limonene, (*S*)‐limonene, (+)‐α‐pinene, (−)‐α‐pinene, terpinolene and sabinene (Figure S23) failed to undergo hydration with the *Rg*CrtC IL144 wildtype.

To assess scalability, biotransformations were performed at volumes of 100, 125, or 500 mL, resulting in the isolation of 52 to 701 mg of products **3, 5, 8, 9, 16**, and **23** which were characterized via NMR (SI NMR spectra, Figure S24). In addition, **13** was produced on gram scale (1.11 g, 70.2% isolated yield) using 50 mM substrate in 125 mL, highlighting the robustness of the reaction setup. *Rg*CrtC IL144 wild‐type demonstrates broad substrate tolerance and excellent regioselectivity across 20 tested terpenes. The best results were obtained with C_13_─C_18_ substrates bearing keto, alcohol, acid, ester, or thioester head groups. Even acid‐labile compounds were successfully hydrated, and the whole‐cell system delivered high‐purity products at preparative scales.^[^ ^34^ ^]^

To identify residues critical for catalysis, enhance enzyme activity, and expand the substrate scope — particularly toward smaller terpenes—we applied a combination of computational modeling and enzyme engineering strategies. (Figure 3a) Using an AlphaFold^[^ ^35^ ^]^‐predicted structure of *Rg*CrtC IL144, we conducted a detailed cavity analysis with Catalophore technology.^[^ ^36^ ^]^ This revealed an elongated substrate‐binding cavity positioned between the enzyme's two β‐barrels, extending to the protein surface. Docking studies using Yasara's VINA^[^ ^37^, ^38^ ^]^ implementation with the natural substrate **1** demonstrated that the terminal prenyl moiety aligns closely with the postulated catalytic residue D268, placing the unactivated double bond within 4 Å of the active site, suitable for protonation (Figure S25). A multiple sequence alignment of 171 CrtC homologs from the NCBI database (using BLAST^[^ ^39^ ^]^ and ClustalW2^[^ ^40^ ^]^) identified 19 out of 21 residues in the active site as highly conserved (88.9%–100%, Figure 3b). In particular, D268, W110, W283, and W285 emerged as key conserved residues. The only notable deviations in *Rg*CrtC IL144 were Met at position 384 (1.8% consensus) and Ile at position 276 (20.5% consensus). To probe the function of these residues, we performed site‐directed mutagenesis. Tryptophan substitutions (W110, W283, and W285) with phenylalanine and alanine drastically reduced or abolished activity, confirming their critical role in carbocation stabilization via cation–π interactions.^[^ ^41^, ^42^ ^]^ Similarly, D268 substitutions with leucine, asparagine, or alanine were detrimental to catalytic function, supporting its role in protonation (Figures S32 and S33).^[^ ^25^ ^]^ Docking all active substrates revealed that their terminal prenyl units consistently occupy a position allowing protonation by D268. This underscores a universal binding mode for terpene substrates across diverse structure (Figures S25–S31).^[^ ^43^ ^]^

**Figure 3:**
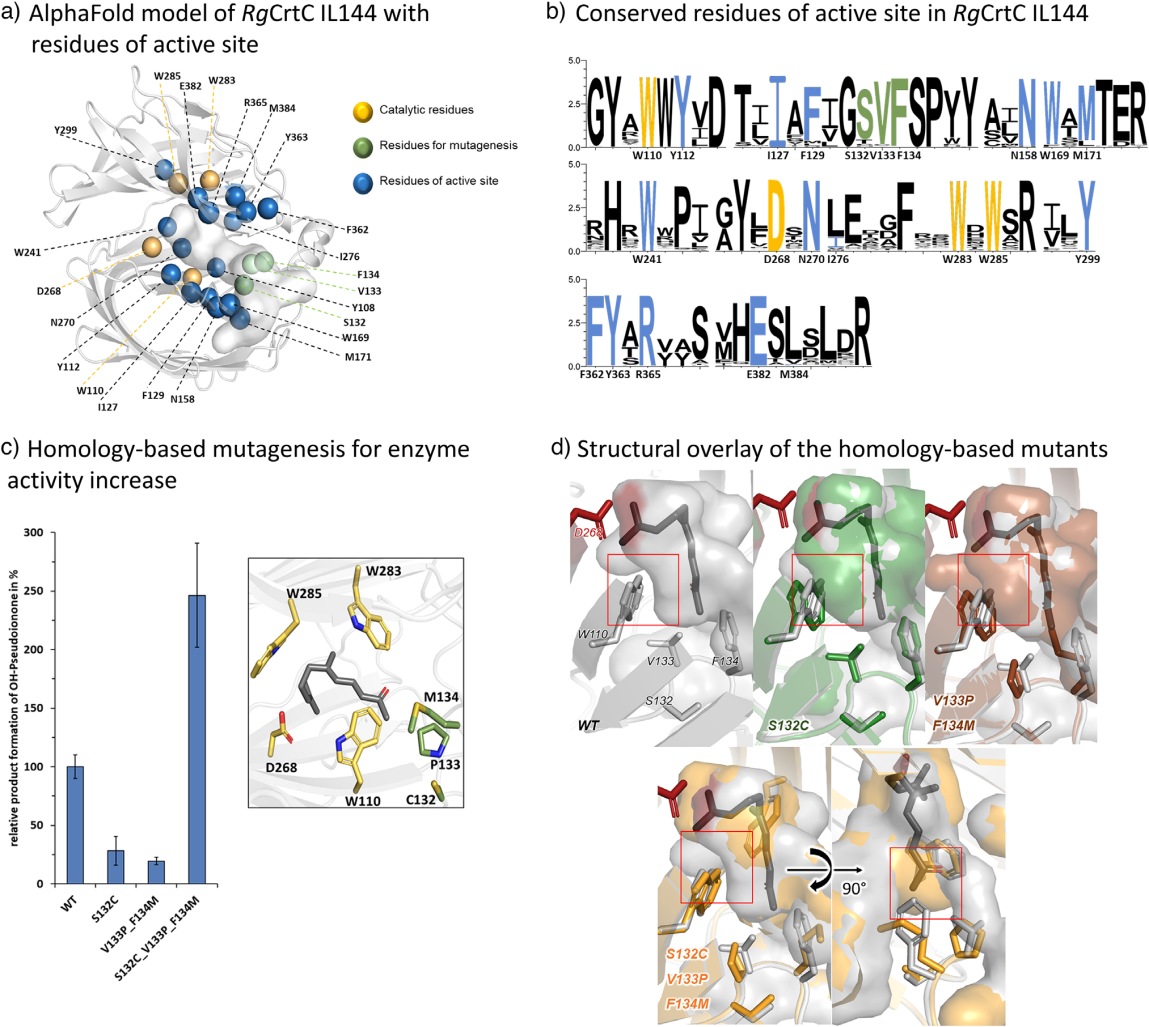
Investigation of crucial amino acids for catalysis and increased activity of *Rg*CrtC IL144 hydration reactions. a) The AlphaFold model displays the active site cavity (light gray surface) without membrane anchor (is not folded correctly during modeling). Important residues like catalytic active site residues (orange), residues for the homology‐based mutagenesis (green) approach and residues of the active site/cavity (blue) are displayed as spheres. b) The Weblogo^[^ ^44^ ^]^ shows the conserved residues in *Rg*CrtC IL144, compared to 171 homologous sequences. The coloring of the positions represents the respective residues analogous to (a). c) Homology‐based variants tested for increasing the hydration activity of *Rg*CrtC IL144. Mutated positions C132, P133 and M134 (green‐yellow/blue) and catalytic important residues D268, W110, W283 and W285 (yellow‐red/blue) are shown as sticks in the docking of 7 (gray/red sticks). Expression and reactions took place in 96 DWPs. Biotransformations were conducted in KPi buffer pH = 7.0, 1 mM 2HPCD, 1 mM of substrate 7 in DMSO, 5 h at 30 °C. The yields represent mean values of technical triplicates (*n* = 3). d) Surface comparison of the *Rg*CrtC variants WT (gray), S132C (green), V133P_F134M (brown) and S132C_V133P_F134M (orange) with docked substrate 7 in *Rg*CrtC S132C_V133P_F134M. Structures were generated with Alphafold. The comparison highlights (red brackets) the repulsive effect on the carbocation stabilizing and π‐stacking W110 by the S132C and the V133P_F134M mutations, while in the triple variant this effect is not observed. The combined mutations also enable precise fitting of the substrate's keto‐methyl group. Please see supporting Figure S38 for full stereoview of the variants.

Based on the cavity, docking, and consensus analyses,^[^ ^45^, ^46^ ^]^ we selected three adjacent residues S132, V133, and F134 for mutagenesis (Figure 3a, green spheres). These positions, located along the active site tunnel and proximal to the functional groups of multiple substrates, were targeted to potentially expand substrate compatibility. Mutations were introduced using residues commonly found at these positions in CrtC homologs. (Figure S34). The resulting single and combinatorial variants (S132C, V133P_F134M, and the triple mutant S132C_V133P_F134M; Figure S35) were tested with substrates **2, 6, 7**, and **11** for terminal hydration. Intriguingly, only the triple variant showed a 2.5‐fold increase in product formation—but exclusively with **7**. This substrate‐specific enhancement suggests an epistatic interaction among the mutations (Figure S36 and Figure S37).^[^ ^47^ ^]^ In silico structural overlays of the variants with the docked substrate **7** highlighted changes in the active site (Figure 3d). Compared to the wild‐type enzyme (gray), both S132C (green) and V133P_F134M (brown) displaced the W110 residue away from the protonation site, likely impairing π‐stacking and carbocation stabilization. However, in the triple variant (orange), W110 maintained its critical positioning, while a new, well‐defined pocket formed to accommodate the keto‐methyl group of **7** (please see supporting Figure S38 for a comprehensive stereoview). This improved binding geometry may account for the enhanced activity with pseudoionone. Such substrate‐specific enhancement and active‐site fine‐tuning reflect the importance of spatial complementarity and active‐site rigidity. Similar mechanisms have been reported for squalene‐hopene cyclases,^[^ ^48^ ^]^ where tight packing around the terminal isoprenoid unit is crucial for carbocation chemistry. Together, these findings highlight the structural and evolutionary rigidity of the carotenoid 1,2‐hydratase active site, which shows limited tolerance to minor residue changes, yet, under precise conditions, can benefit from synergistic mutations.^[^ ^49^, ^50^ ^]^

Our study underscores the catalytic potential of the carotenoid 1,2‐hydratase from *Rubrivivax gelatinosus* IL144 as a robust and versatile biocatalyst. Operating under cofactor‐independent conditions, this enzyme enables highly regioselective terminal hydration across a wide array of terpenes, varying in both chain length and functional head groups–at gram‐scale. Its compatibility with lyophilized whole‐cell preparations offers a practical advantage for handling and storage. Furthermore, we demonstrate its seamless integration into one‐pot cascade reactions. Through active site and tunnel engineering, we identified key catalytic residues and revealed a striking evolutionary conservation of the hydratase's structural core. This work provides a solid foundation for expanding the biocatalytic toolbox and underscores the untapped potential of this underexplored enzyme family.

## Conflict of Interests

The authors declare no conflict of interest.

## Supporting information



Supporting Information

## Data Availability

The data that support the findings of this study are available from the corresponding author upon reasonable request.

## References

[1] N. Armanino , J. Charpentier , F. Flachsmann , A. Goeke , M. Liniger , P. Kraft , Angew. Chem. Int. Ed. 2020, 59, 16310–16344. 10.1002/anie.202005719 32453472

[2] F. M. Majewski , L. F. Marek , Ind. Eng. Chem. 1938, 30, 203–210.

[3] R. W. Taft Jr , J. Am. Chem. Soc. 1952, 74, 5372–5376.

[4] M. L. Bender , K. A. Connors , J. Am. Chem. Soc. 1961, 83, 4099–4100.

[5] A. J. Kresge , Y. Chiang , P. H. Fitzgerald , R. S. McDonald , G. H. Schmid , J. Am. Chem. Soc. 1971, 93, 4907–4908.

[6] D. J. Nelson , C. Brammer , R. Li , Tetrahedron Lett. 2009, 50, 6454–6456.

[7] J. E. McMurry , Organic Chemistry 2011, Cengage Learning.

[8] A. Alchenberger , C. Berbez , C. Finn , D. Lelievre , M. A. Lovchik , R. Poignon‐Martel , G. Romey , A. Fr , P. Fr , D. Lelievre , K. Ch , M. Alan , D. Ch , Organic Compounds, GB 2515128 (Givaudan), 2013.

[9] R. Ramachanderan , B. Schaefer , in ChemTexts, Lily‐of‐the‐Valley Fragrances, Vol. 5, Springer International Publishing, 2019.

[10] C. Sell , in The Chemistry of Fragrances, 2nd ed., Royal Society of Chemistry, Cambridge, U.K 2007, pp. 52–131.

[11] P. N. Davey , M. J. Earle , J. T. Hamill , S. P. Katdare , D. W. Rooney , K. R. Seddon , Green Chem. 2010, 12, 628.

[12] J. Jin , U. Hanefeld , Chem. Commun. 2011, 47, 2502–2510. 10.1039/c0cc04153j 21243161

[13] B. S. Chen , L. G. Otten , U. Hanefeld , Biotechnol. Adv. 2015, 33, 526–546. 25640045 10.1016/j.biotechadv.2015.01.007

[14] A. Hiseni , I. W. C. E. Arends , L. G. Otten , ChemCatChem 2015, 7, 29–37.

[15] M. Engleder , H. Pichler , Appl. Microbiol. Biotechnol. 2018, 102, 5841–5858. 29785499 10.1007/s00253-018-9065-7 PMC6013536

[16] R. M. Demming , M. P. Fischer , J. Schmid , B. Hauer , Curr. Opin. Chem. Biol. 2018, 43, 43–50. 29156448 10.1016/j.cbpa.2017.10.030

[17] M. Engleder , G. A. Strohmeier , H. Weber , G. Steinkellner , E. Leitner , M. Müller , D. Mink , M. Schürmann , K. Gruber , H. Pichler , Angew. Chem. Int. Ed. 2019, 58, 7480–7484. 10.1002/anie.201901462 PMC6563698 30848865

[18] R. M. Demming , K. B. Otte , B. M. Nestl , B. Hauer , ChemCatChem 2017, 9, 758–766.

[19] R. M. Demming , S. C. Hammer , B. M. Nestl , S. Gergel , S. Fademrecht , J. Pleiss , B. Hauer , Angew. Chem. 2019, 131, 179–183. 10.1002/anie.201810005 PMC6471033 30256501

[20] M. Gajdoš , J. Wagner , F. Ospina , A. Köhler , M. K. M. Engqvist , S. C. Hammer , Angew. Chem. Int. Ed. 2023, 62, 1–6. 10.1002/anie.202215093 PMC10107627 36511829

[21] M. Philippe , in Methode for Producing an Alkene Comprising the Step of Converting an Alcohol by an Enzymatic Dehydration Step, *WO2011076689A1* , 2011.

[22] M. Engleder , M. Horvat , A. Emmerstorfer‐Augustin , T. Wriessnegger , S. Gabriel , G. Strohmeier , H. Weber , M. Müller , I. Kaluzna , D. Mink , M. Schürmann , H. Pichler , PLoS One 2018, 13, e0192653. 29420618 10.1371/journal.pone.0192653 PMC5805349

[23] S. Steiger , A. Mazet , G. Sandmann , Arch. Biochem. Biophys. 2003, 414, 51–58. 12745254 10.1016/s0003-9861(03)00099-7

[24] A. Hiseni , I. W. C. E. Arends , L. G. Otten , Appl. Microbiol. Biotechnol. 2011, 91, 1029–1036. 21590288 10.1007/s00253-011-3324-1 PMC3145076

[25] A. Hiseni , L. G. Otten , I. W. C. E. Arends , Appl. Microbiol. Biotechnol. 2016, 100, 1275–1284. 26481619 10.1007/s00253-015-6998-y PMC4717167

[26] A. Schneider , C. Curado , T. B. Lystbaek , S. Osuna , B. Hauer , Angew. Chem. Int. Ed. 2023, 62, e202301607. 10.1002/anie.202301607 36939150

[27] C. W. Koo , F. J. Tucci , Y. He , A. C. Rosenzweig , Science 2022, 375, 1287–1291. 35298269 10.1126/science.abm3282 PMC9357287

[28] G. Lozano Terol , J. Gallego‐Jara , R. A. Sola Martínez , A. Martínez Vivancos , M. Cánovas Díaz , T. de Diego Puente , Front. Microbiol. 2021, 12, 1–12. 10.3389/fmicb.2021.682001 PMC8257044 34234760

[29] A. Schütz , F. Bernhard , N. Berrow , J. F. Buyel , F. Ferreira‐da‐Silva , J. Haustraete , J. van den Heuvel , J. E. Hoffmann , A. de Marco , Y. Peleg , S. Suppmann , T. Unger , M. Vanhoucke , S. Witt , K. Remans , STAR Protoc. 2023, 4, 102572. 37917580 10.1016/j.xpro.2023.102572 PMC10643540

[30] M. Bassereau , A. Chaintreau , S. Duperrex , D. Joulain , H. Leijs , G. Loesing , N. Owen , A. Sherlock , C. Schippa , P. J. Thorel , M. Vey , J. Agric. Food Chem. 2007, 55, 25–31. 17199309 10.1021/jf062028l

[31] J. M. Pineda‐Ríos , J. Cibrián‐Tovar , L. M. Hernández‐Fuentes , R. M. López‐Romero , L. Soto‐Rojas , J. Romero‐Nápoles , C. Llanderal‐Cázares , L. F. Salomé‐Abarca , Molecules 2021, 26, 2861. 34065875 10.3390/molecules26102861 PMC8150320

[32] C. Zhang , M. Li , G. R. Zhao , W. Lu , Microb. Cell Fact. 2019, 18, 1–10. 31547812 10.1186/s12934-019-1211-0 PMC6757357

[33] T. E. Goodwin , F. D. Brown , R. W. Counts , N. C. Dowdy , P. L. Fraley , R. A. Hughes , D. Z. Liu , C. D. Mashburn , J. D. Rankin , R. S. Roberson , K. D. Wooley , E. L. Rasmussen , S. W. Riddle , H. S. Riddle , S. Schulz , J. Nat. Prod. 2002, 65, 1319–1322. 12350155 10.1021/np010647c

[34] H. Lei , Q. Ma , Z. Wang , D. Zhang , X. Huang , M. Qin , H. Ma , W. Wang , Y. Cao , ACS Nano 2023, 17, 16870–16878. 37646337 10.1021/acsnano.3c03807

[35] J. Jumper , R. Evans , A. Pritzel , T. Green , M. Figurnov , O. Ronneberger , K. Tunyasuvunakool , R. Bates , A. Žídek , A. Potapenko , A. Bridgland , C. Meyer , S. A. A. Kohl , A. J. Ballard , A. Cowie , B. Romera‐Paredes , S. Nikolov , R. Jain , J. Adler , T. Back , S. Petersen , D. Reiman , E. Clancy , M. Zielinski , M. Steinegger , M. Pacholska , T. Berghammer , S. Bodenstein , D. Silver , O. Vinyals , et al., Nature 2021, 596, 583–589. 34265844 10.1038/s41586-021-03819-2 PMC8371605

[36] G. C. K. Gruber , G. Steinkellner , Determining Novel Enzymatic Functionalities Using Three‐Dimensional Point Clouds Representing Physico Chemical Properties of Protein Cavities 2020, WO2014080005A1.

[37] O. Trott , A. J. Olson , J. Comput. Chem. 2010, 31, 455–461. 19499576 10.1002/jcc.21334 PMC3041641

[38] E. Krieger , G. Vriend , Bioinformatics 2014, 30, 2981–2982. 24996895 10.1093/bioinformatics/btu426 PMC4184264

[39] S. F. Altschul , T. L. Madden , A. A. Schäffer , J. Zhang , Z. Zhang , W. Miller , D. J. Lipman , Nucleic Acids Res. 1997, 25, 3389–3402. 9254694 10.1093/nar/25.17.3389 PMC146917

[40] M. A. Larkin , G. Blackshields , N. P. Brown , R. Chenna , P. A. McGettigan , H. McWilliam , F. Valentin , I. M. Wallace , A. Wilm , R. Lopez , Bioinformatics 2007, 23, 2947–2948. 17846036 10.1093/bioinformatics/btm404

[41] T. Hoshino , T. Sato , Chem. Commun. 2002, 2, 291–301. 10.1039/b108995c 12120044

[42] J. Ludwig , C. Curado‐Carballada , S. C. Hammer , A. Schneider , S. Diether , N. Kress , S. Ruiz‐Barragán , S. Osuna , B. Hauer , Angew. Chem. Int. Ed. 2024, 63, 63. 10.1002/anie.202318913 38270537

[43] J. W. Torrance , G. L. Holliday , J. B. O. Mitchell , J. M. Thornton , J. Mol. Biol. 2007, 369, 1140–1152. 17466330 10.1016/j.jmb.2007.03.055

[44] G. E. Crooks , G. Hon , J. M. Chandonia , S. E. Brenner , Genome Res. 2004, 14, 1188–1190. 15173120 10.1101/gr.849004 PMC419797

[45] M. Lehmann , C. Loch , A. Middendorf , D. Studer , S. F. Lassen , L. Pasamontes , A. P. G. M. Van Loon , M. Wyss , Protein Eng. 2002, 15, 403–411. 12034860 10.1093/protein/15.5.403

[46] H. Jochens , U. T. Bornscheuer , ChemBioChem 2010, 11, 1861–1866. 20680978 10.1002/cbic.201000284

[47] C. Fröhlich , H. A. Bunzel , K. Buda , A. J. Mulholland , M. W. van der Kamp , P. J. Johnsen , H. K. S. Leiros , N. Tokuriki , Nat. Catal. 2024, 7, 499–509. 38828429 10.1038/s41929-024-01117-4 PMC11136654

[48] I. Kaneko , Y. Terasawa , T. Hoshino , Chem. ‐ Eur. J. 2018, 24, 11139–11157. 29732636 10.1002/chem.201801668

[49] D. L. Trudeau , D. S. Tawfik , Curr. Opin. Biotechnol. 2019, 60, 46–52. 30611116 10.1016/j.copbio.2018.12.002

[50] Y. J. Solano , M. P. Everett , K. S. Dang , J. Abueg , P. D. Kiser , Nat. Chem. Biol. 2024, 20, 779–788. 38355721 10.1038/s41589-024-01554-z PMC11142922

