# Supplementary material for: Regioselective Hydration of Terpenes with Cofactor‐Independent Carotenoid 1,2‐Hydratase
Source: Angew Chem Int Ed Engl. 2025 Jul 17;64(36):e202505942. doi: 10.1002/anie.202505942 (PMC12402844; doi:10.1002/anie.202505942)
Supplement: Supplementary file 1 — Supporting Information [file ANIE-64-e202505942-s001.docx]

Supporting Information:

Regioselective Hydration of Terpenes with Cofactor

Independent Carotenoid-1,2-Hydratase

Philip Horz^[a]^, Natalie Härterich^[a]^ Andreas Schneider^[a]^, Nicolas D. Travnicek^[a]^, Bettina M. Nestl^[b]^, Ursula Kahler^[b]^ and Bernhard Hauer*^[a]^

[a] P. Horz, N. Härterich, Dr. A. Schneider, N. D. Travnicek, Prof. Dr. B. Hauer
Department Department of Technical Biochemistry
Institution Institute of Biochemistry and Technical Biochemistry, University of Stuttgart
Allmandring 31, 70569 Stuttgart (Germany)
E-mail: Bernhard.hauer@itb.uni-stuttgart.de

[b] Dr. B. M. Nestl, Dr. U. Kahler
Innophore GmbH
Am Eisernen Tor 3, 8010 Graz (Austria)

**Table of content**

[1 Supporting figures and tables 4](#_Toc199283916)

[2 Materials and methods 36](#_Toc199283917)

[2.1 Materials 36](#_Toc199283918)

[2.1.1 Chemicals 36](#_Toc199283919)

[2.1.2 *E.coli strains* 36](#_Toc199283920)

[2.1.3 Molecular biological kits 36](#_Toc199283921)

[2.1.4 Buffer and media 36](#_Toc199283922)

[2.1.5 Primer 36](#_Toc199283923)

[2.2 General analytics 37](#_Toc199283924)

[2.2.1 Nuclear Magnetic Resonance 37](#_Toc199283925)

[2.2.2 Gas chromatography 37](#_Toc199283926)

[2.2.3 HPLC/MS 38](#_Toc199283927)

[2.2.4 Preparative HPLC 38](#_Toc199283928)

[2.3 Enzymatic preparative scale reactions 39](#_Toc199283929)

[2.3.1 Preparative biotransformation of hydroxyfarnesylacetone 3 39](#_Toc199283930)

[2.3.2 Preparative biotransformation of hydroxygeranylacetone 8 39](#_Toc199283931)

[2.3.3 Preparative biotransformation of hydroxypseudoionone 9 40](#_Toc199283932)

[2.3.4 Preparative biotransformation of hydroxyhomofarnesol 13 40](#_Toc199283933)

[2.3.5 Preparative biotransformation of hydroxyfarnesol 5 41](#_Toc199283934)

[2.3.6 Preparative biotransformation of hydroxygeranyllinalool 16 41](#_Toc199283935)

[2.3.7 Preparative biotransformation of hydroxy-α-farnesene 23 42](#_Toc199283936)

[2.4 Chemical synthesis 43](#_Toc199283937)

[2.4.1 Synthesis of geranylisopropanol 43](#_Toc199283938)

[2.4.2 Synthesis of Calmusol 44](#_Toc199283939)

[2.4.3 Synthesis of geranylthioacetate 38 44](#_Toc199283940)

[2.5 General methods 46](#_Toc199283941)

[2.5.1 Cloning of *Rg*CrtC gens and Funclib variants 46](#_Toc199283942)

[2.5.2 Expression-study of *Rg*CrtCs 46](#_Toc199283943)

[2.5.3 Expression for WC-biotransformations and purification 47](#_Toc199283944)

[2.5.4 Expression for 96-deep well plate (DWP) screening (homology-based mutagenesis) 47](#_Toc199283945)

[2.5.5 Semi-purification of *Rg*CrtC 47](#_Toc199283946)

[2.5.6 Purification of *Rg*CrtC 47](#_Toc199283947)

[2.5.7 Mutagenesis of *Rg*CrtC IL144 48](#_Toc199283948)

[2.5.8 Biotransformations for lycopene wild type reaction 48](#_Toc199283949)

[2.5.9 Biotransformations for expression study, reaction parameter optimization and active-site residues 49](#_Toc199283950)

[2.5.10 Biotransformations with semi purified *Rg*CrtC_IL144 50](#_Toc199283951)

[2.5.11 Biotransformations with purified *Rg*CrtC_IL144 50](#_Toc199283952)

[2.5.12 Biotransformations for substrate-screening 51](#_Toc199283953)

[2.5.13 Cascade reaction of aldol condensation and subsequent hydration 51](#_Toc199283954)

[2.5.14 Biotransformations for 96 DWPs (Homology-based mutagenesis) 52](#_Toc199283955)

[2.5.15 Preparative scale reactions 52](#_Toc199283956)

[2.5.16 Cavity analysis and YASARA^®^ docking 53](#_Toc199283957)

[2.5.17 Generating FuncLib 54](#_Toc199283958)

[2.5.18 Multiple sequence alignment and weblogo 54](#_Toc199283959)

[3 NMR spectra 61](#_Toc199283960)

[Hydroxypseudoionone 9: 64](#_Toc199283961)

[Hydroxyhomofarnesol 13: 65](#_Toc199283962)

[Hydroxyfarnesol 12: 66](#_Toc199283963)

[Hydroxygeranyllinalool 16: 67](#_Toc199283964)

[Hydroxy-α-farnesene 23: 68](#_Toc199283965)

[Gernylisorpopanol: 69](#_Toc199283966)

[(E)-5,9-dimethyldeca-4,8-dien-1-ol (calmusol): 70](#_Toc199283967)

[Geranylthioacete: 71](#_Toc199283968)

[4 Gen sequences and plasmid cards 72](#_Toc199283969)

[4.1 Carotenoid-1,2, hydratases 72](#_Toc199283970)

[4.2 Plasmid cards 73](#_Toc199283971)

# Supporting figures and tables


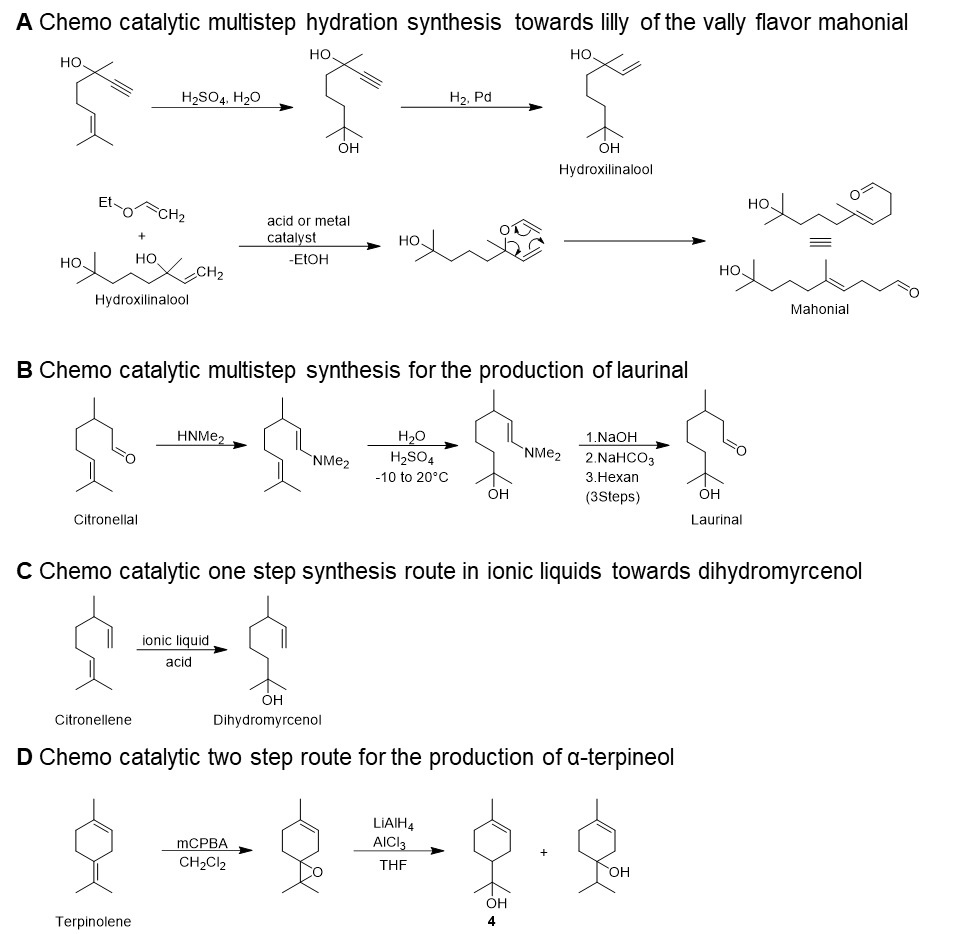


Figure S1: Chemical multistep synthesis for regioselective addition of water towards industrial interesting products. **A)** Four step state of the art synthesis towards mahonial from 3,7-dimethyloct-6-en-1-yn-3-ol. 3,7-dimethyloct-6-en-1-yn-3-ol is firstly hydrated in aqueous sulfuric acid to compound and subsequently hydrogenated by using a palladium catalyst to generate hydroxylinalool. Hydroxylinalool and ethyl viniyl ether are converted by the use of a metal or acid catalyst with elimination of ethanol and subsequent Claisen rearrangement.^[1]^ **B)** The production of the lily of the vally fragrance laurinal starts with the educt citronellal**.** Citronellal reacts in the presence of dimethylamine to produce an enamine. This is added to aqueous sulphuric acid at -10°C in a further step. The reaction is then heated to room temperature where hydration takes place. In a final step, the hydrated product is neutralized and washed to obtain the hydrated aldehyde laurinal.^[2]^ **C)** The hydration of (+/-)-β-citronellen to dihydromyrcenol takes place in ionic liquids with acid as a catalyst, but with high activity/selectivity tradeoffs^[3]^ **D)** The two-step synthesis route to α-terpineol **4** starts with terpinolene, which reacts in the presence of m-chloroperbencoicacid (mCPBA) in anhydrous CH_2_Cl_2_ to form terpinolene oxide. Terpinolene oxide reacts with LiAlH and AlCl_2_ in THF to give the hydrated product **4** and the by-product terpinen-4-ol.^[4]^


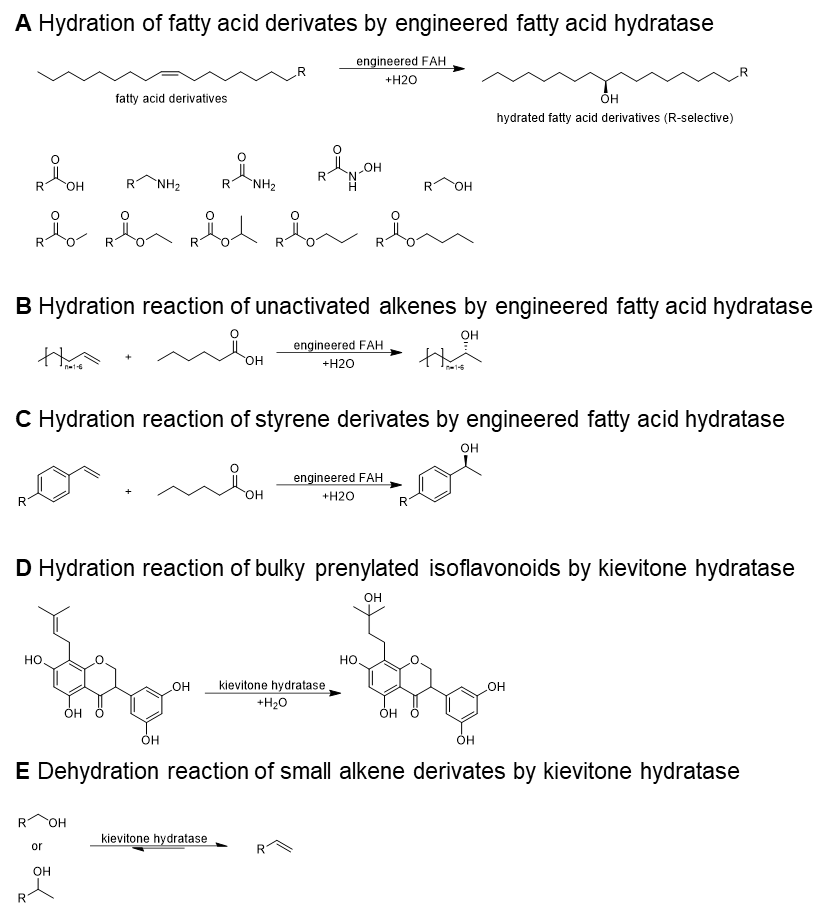


Figure S2: Regio- and stereoselective addition of water to C-C double bonds catalyzed by different hydratases. Fatty acid hydratases (FAHs) add water cofactor dependent to unactivated C-C double bonds. By means of enzyme engineering, the substrate scope of these enzymes could be extended so that fatty acid derivatives **(A)** ^[5]^, short alkenes **(B)** ^[6]^ and styrene derivatives **(C)** ^[7]^ could be converted. Another hydratase that hydrates non-activated C-C double bonds of prenyl units **(D)** ^[8]^ and dehydrates short alkene derivatives **(E)** ^[9]^ are kieviton hydratases.

Figure S3: Natural reaction of Carotenoid-1,2-hydratases with their natural substrate lycopene **1** to the single hydrated product 1-HO-lycopene and the double hydrated product 1,1-(OH)_2_-lycopene.^[10–12]^


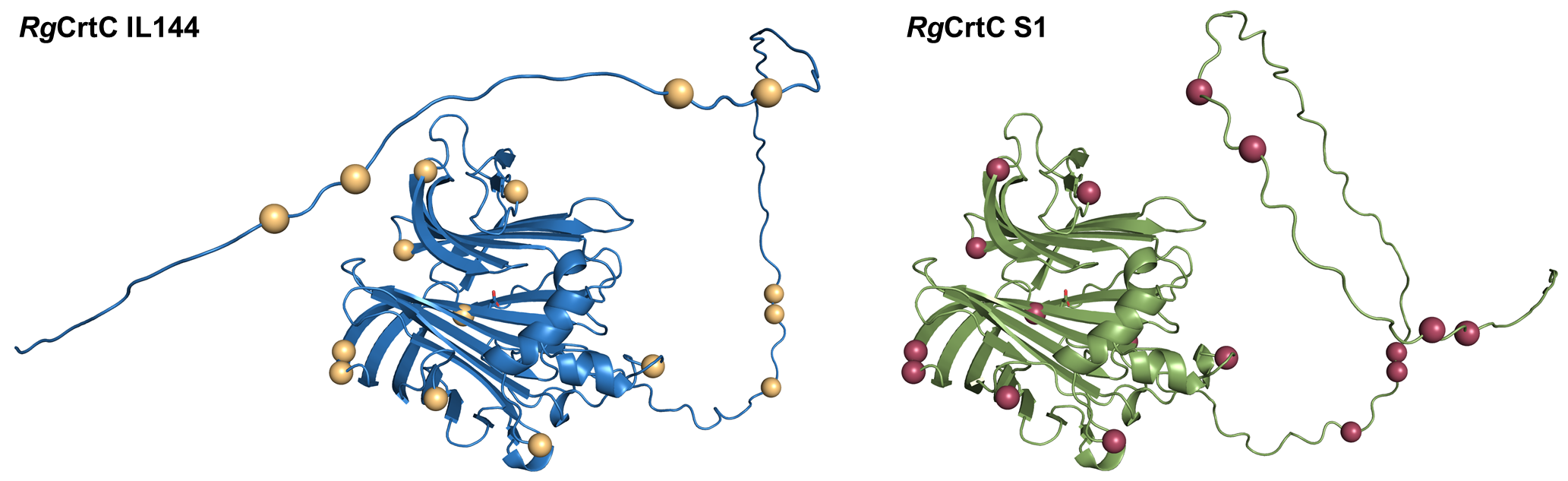


|  | **Change in amino acid** | |  |
| --- | --- | --- | --- |
| **Position** | ***Rg*CrtC IL144** | ***Rg*CrtC S1** |  |
| **15** | D | V | **Membrane anchor** |
| **18** | L | V |  |
| **30** | E | G |  |
| **34** | P | A |  |
| **66** | P | A |  |
| **67** | V | L |  |
| **71** | R | G |  |
| **88** | A | V |  |
| **145** | N | K | **Enzyme core** |
| **207** | I | V |  |
| **258** | N | R |  |
| **259** | A | V |  |
| **267** | V | L |  |
| **293** | G | S |  |
| **340** | D | G |  |
| **352** | F | L |  |
| **406** | S | A |  |

Figure S4: Changes in amino acids of *Rg*CrtC IL144 and *Rg*CrtC S1. Alpha fold ^[13]^ models of carotenoid-1,2-hydratases of *Rg*CrtC IL144 (blue) and *Rg*CrtC S1 (green). Spheres (IL144 orange and S1 red) represent the position of the changed amino acids in each enzyme on the membrane anchor and the enzyme core.

**Expression system investigation:**

When we compared our expression setup system of pDHE1650/*E.coli* ITB94 (low copy plasmid, tightly regulated expression) with the literature established pET-22b(+)/*E.coli* BL21(DE3) (high copy plasmid, leaky expression), we determined a remarkable 50-fold increase in product **3** formation (35% *vs.* 0.6%; Table 1 entry 2 and 3). This discrepancy in activity might be attributed to the low copy maintenance and tight regulation of the expression system, which could influence enzyme folding and resulting activity.^[14,15]^Furthermore, we found that the activity of the expressed enzyme in LB medium was 1.4‑fold (pDHE1650/*E.coli* ITB94) or 3 fold (pET-22b(+)/*E.coli* BL21(DE3)) inferior to the enzymes expressed in TB medium, despite similar expression levels (Figure S6). These findings highlight the critical importance of selecting the appropriate expression system to yield active enzyme.


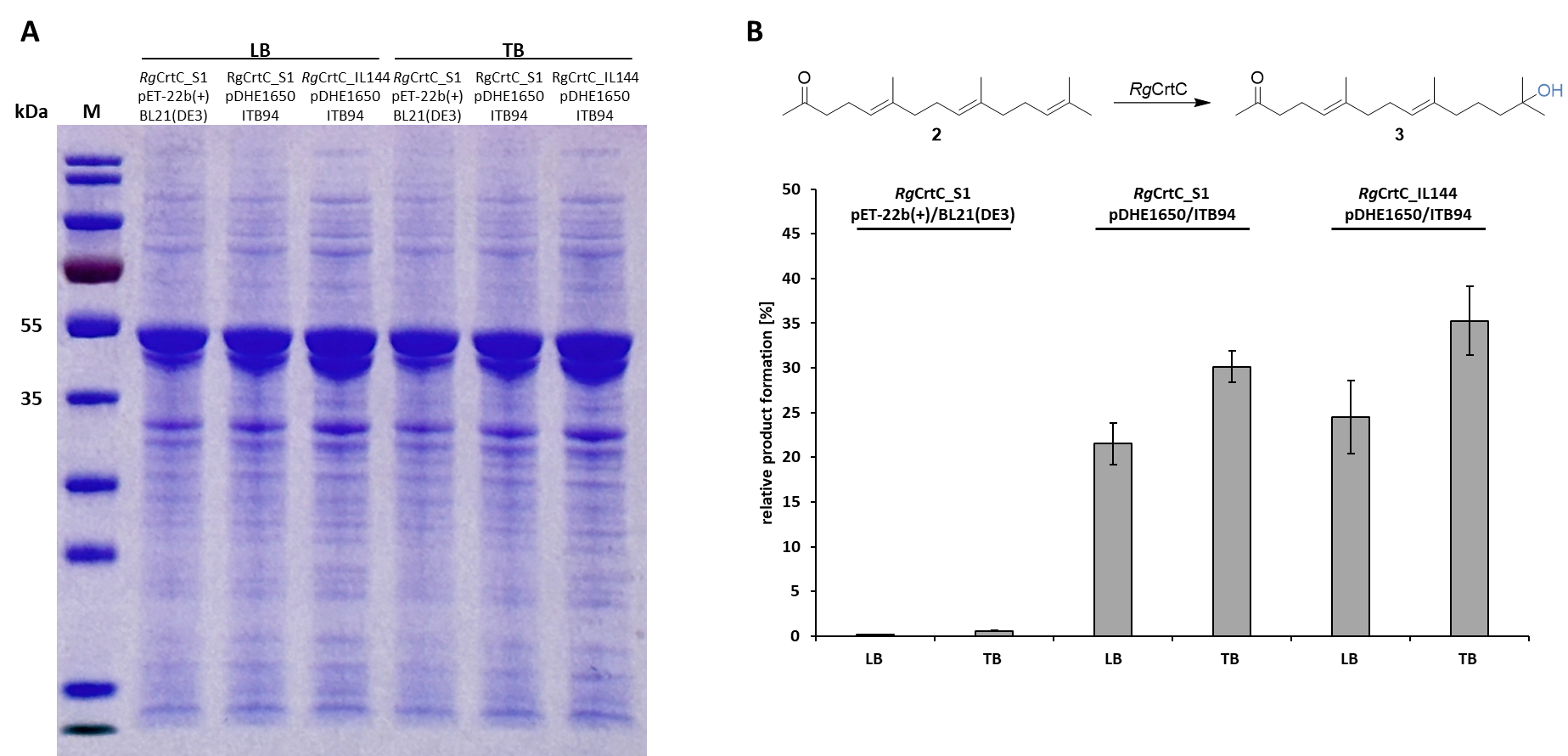


Figure S5: Expression and activity study of *Rg*CrtCs with different expression systems. **A)** The successful expression of *Rg*CrtC S1 in pET-22b(+)/*E.coli* BL21(DE3) and pDHE/*E.coli* ITB94, as well as the expression of *Rg*CrtC IL144 in the pDHE/*E.coli* ITB94 system in LB- and TB-media was controlled by SDS-PAGE. The expected molecular weight of the hydratases is 47kDa. All expression systems showed good overexpression. Uncropped SDS-PAGE is shown in figure S39. **B)** The activity of the hydratases was tested in the WC system with standard conditions (100 mg_cww_/mL in KPi buffer, 50 mM pH = 7.0, 1 mM substrate, 24 h and 30 °C) with substrate **2** in triplicates. The relative product formation of **3** was analyzed.

**Reaction parameter optimization:**

We found that the optimal temperature and pH for converting **2** as a model substrate were 50 mM KPi pH 7.0 at 30 °C, consistent with literature data.^[11]^ Despite exploring different buffers, pH values, and temperatures, we did not observe any improvement in the aforementioned setup (100 mg_cww_/mL in 50 mM potassium phosphate buffer (KPi buffer), pH = 7.0) (Figure S7 and S8). Importantly, we discovered that *Rg*CrtC IL144 is operational within a pH range of pH 5-8 without any loss of activity (Figure S7). In addition, our studies revealed that there is minimal difference in substrate conversion using wet or lyophilized whole cells (Figure S9). This simplifies the use, handling and storage of the hydratase, making it interesting for industrial applications. Furthermore, storage ability at room temperature has been demonstrated for at least two years with only a slight decrease in activity (Figure S9). Our examination of cofactor dependence indicated that adding NADH, NADPH and FAD individually or in combination to the reaction to semi purified enzyme from membrane fractions did not result in increased activity (Figure S10). We also found that 2HPCD, which was also employed *in vivo* biocatalysis of other membrane-bound enzymes^[16]^, at concentrations of 0.25-10 mM (Figure S11), enhanced product formation by 1.2-and 1.4-fold, respectively. The best setup (100 mg_cww_/mL in 50 mM potassium phosphate buffer (KPi buffer), pH = 7.0) involved adding 2-HPCD in equimolar quantities to the substrate.


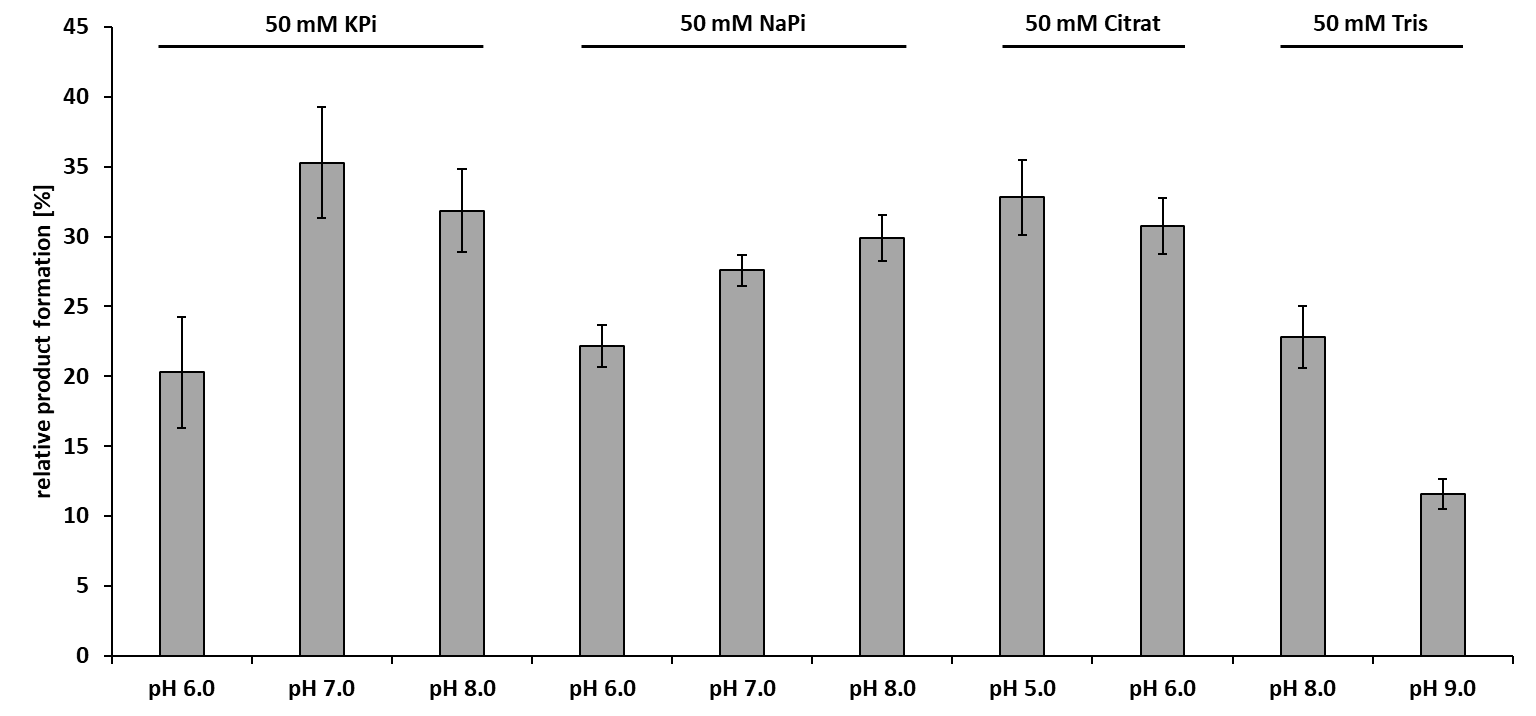


Figure S6: Reaction parameter optimization of *Rg*CrtC IL144 WC-System (100 mg_cww_/mL in different buffers, 1 mM substrate, 24 h and 30 °C) in different buffers and pH-values. The reactions were tested with **2** as a model substrate in triplicates. The relative product formation of **3** was analyzed.


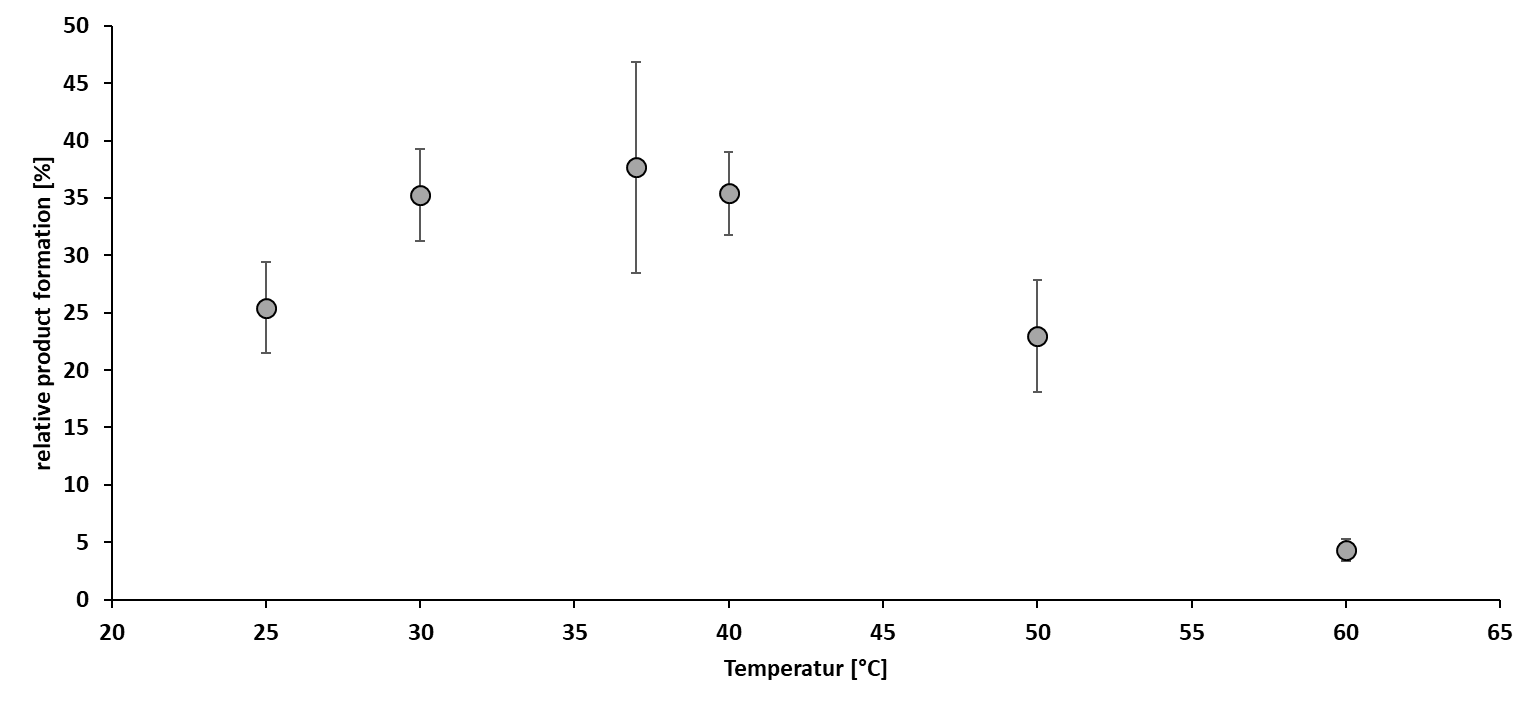


Figure S7: Reaction parameter optimization of *Rg*CrtC IL144 WC-System (100 mg_cww_/mL in KPi buffer, 50 mM pH = 7.0, 1 mM substrate, 24 h) at different temperatures. The reactions were tested with **2** as a model substrate in triplicates. The relative product formation of **3** was analyzed.


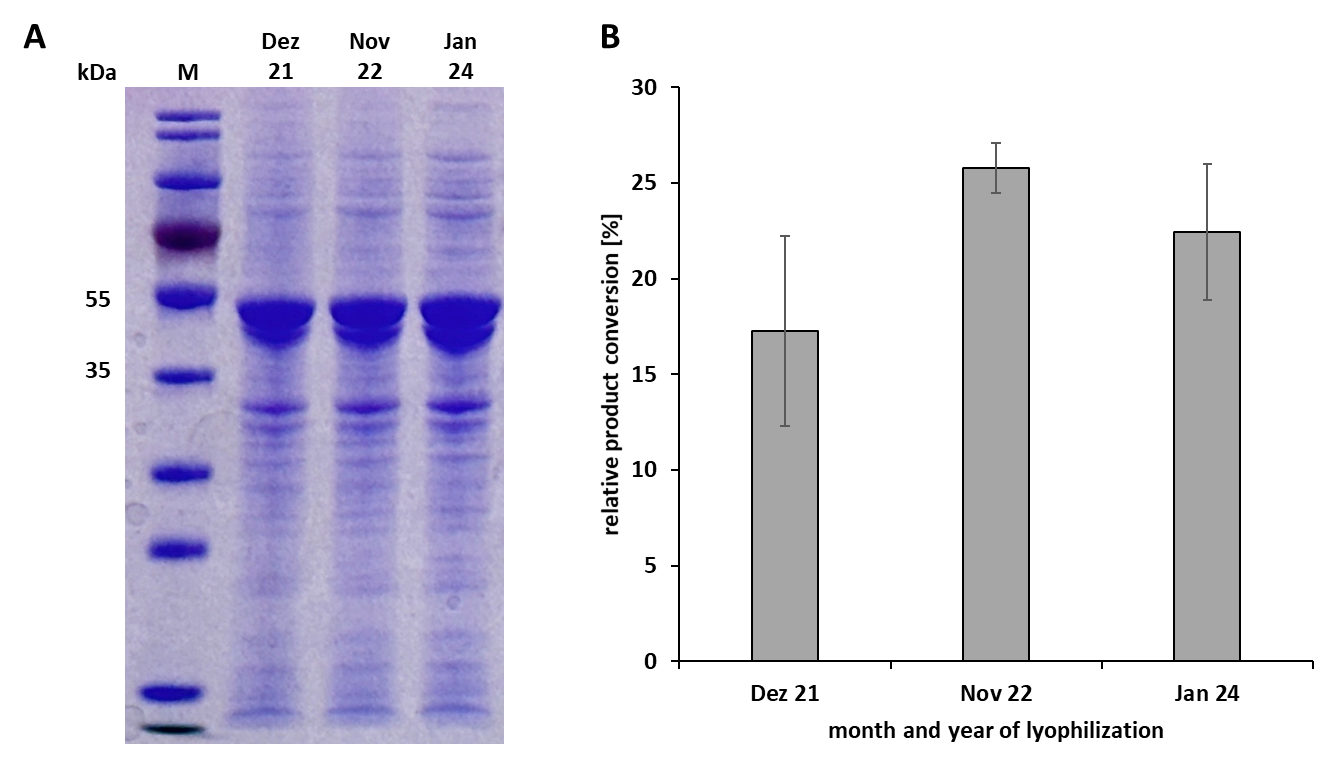


Figure S8: Storability of lyophilized whole cells and activity assay. **A)** Expression levels of the different lyophilized cell batches were compared by SDS-PAGE. Uncropped SDS-PAGE is shown in figure S39. **B)** Activity of the different lyophilized cell batches was investigated by using the standard protocol (100 mg_cww_/mL in KPi buffer, 50 mM pH = 7.0, 1 mM substrate, 24 h and 30 °C) and the substrate **2** in triplicates. The relative product formation of **3** was analyzed.


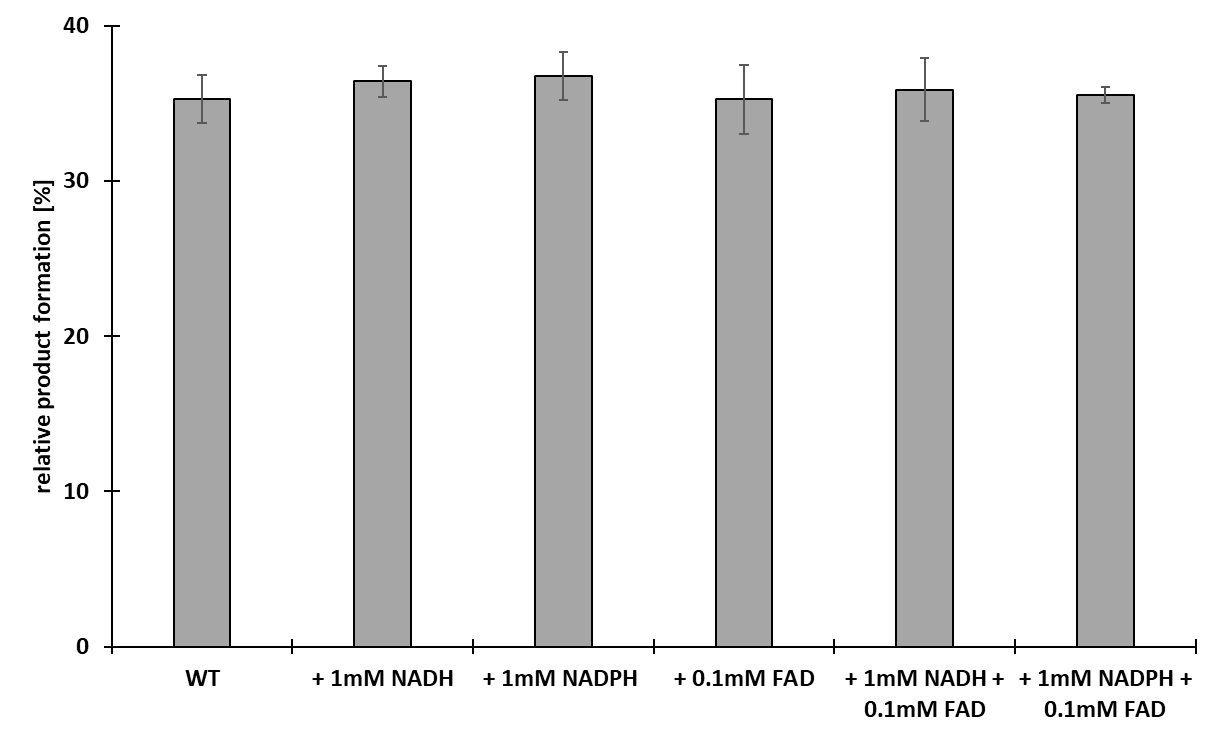


Figure S9: Cofactor dependence assay of *Rg*CrtC IL144 with semi-purified enzyme solution. The cofactor dependency of the hydratases was tested with solubilized enzyme with standard conditions (100 mg_cww_/mL in KPi buffer, 50 mM pH = 7.0, 1 mM substrate, 24 h and 30 °C) by adding either NADH, NADPH or FAD or a mix of them with substrate **2** in triplicates. The relative product formation of **3** was analyzed.


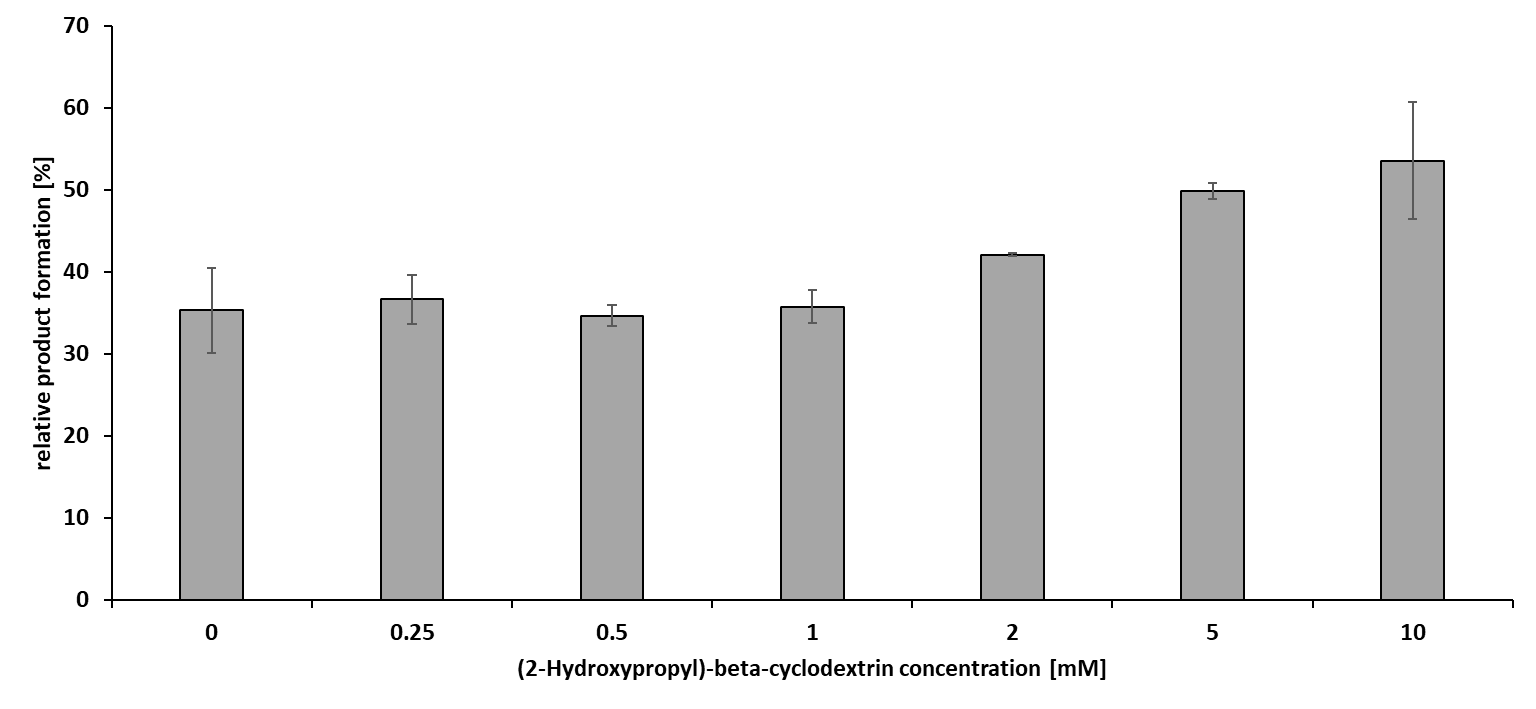


Figure S10: Influence of 2-HPCD on the activity of *Rg*CrtC IL144 in the WC-system. The 2-HPCD influence on the hydration reaction was tested in the WC system with standard conditions (100 mg_cww_/mL in KPi buffer, 50 mM pH = 7.0, 1 mM substrate, 24 h and 30 °C) by adding different concentrations of 2-HPCD to the reaction with substrate **2** in triplicates. The relative product formation of **3** was analyzed.


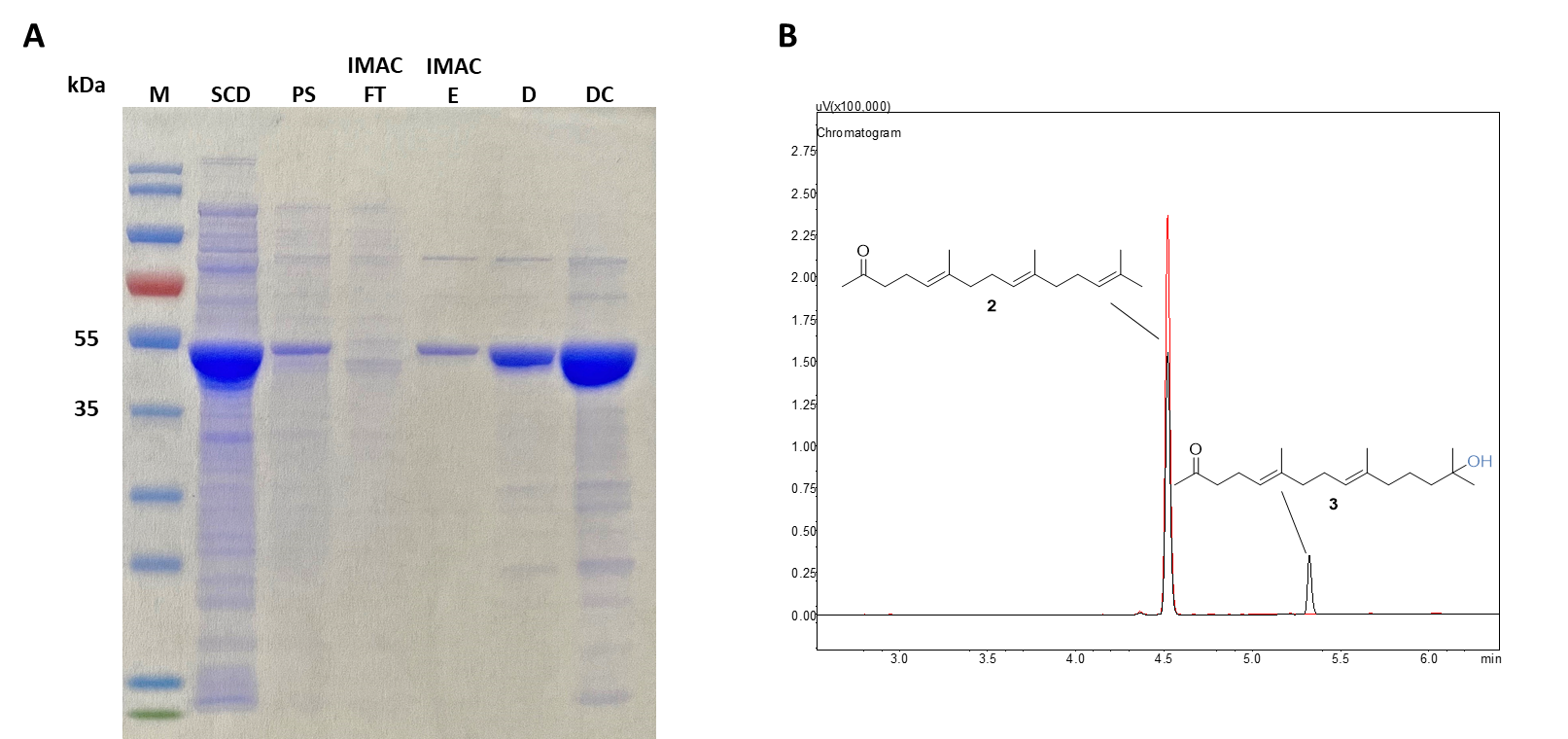


Figure S11: Purification and activity assay of *Rg*CrtC IL144. **A)** The enzyme was purified via immobilized metal ion affinity chromatography (IMAC). The following samples were analyzed by SDS-PAGE: SDS: supernatant after cell disruption, PS: pellet supernatant after solubilization, IMAC FT: IMAC flow-through, IMAC E: IMAC elution, D: purified enzyme after desalting and DC: desalted and concentrated purified enzyme. Uncropped SDS-PAGE is shown in figure S40. **B)** Activity assay of the purified enzyme (black) and buffer control (red) were performed with substrate **2** in triplicates. Product **3** was analyzed.


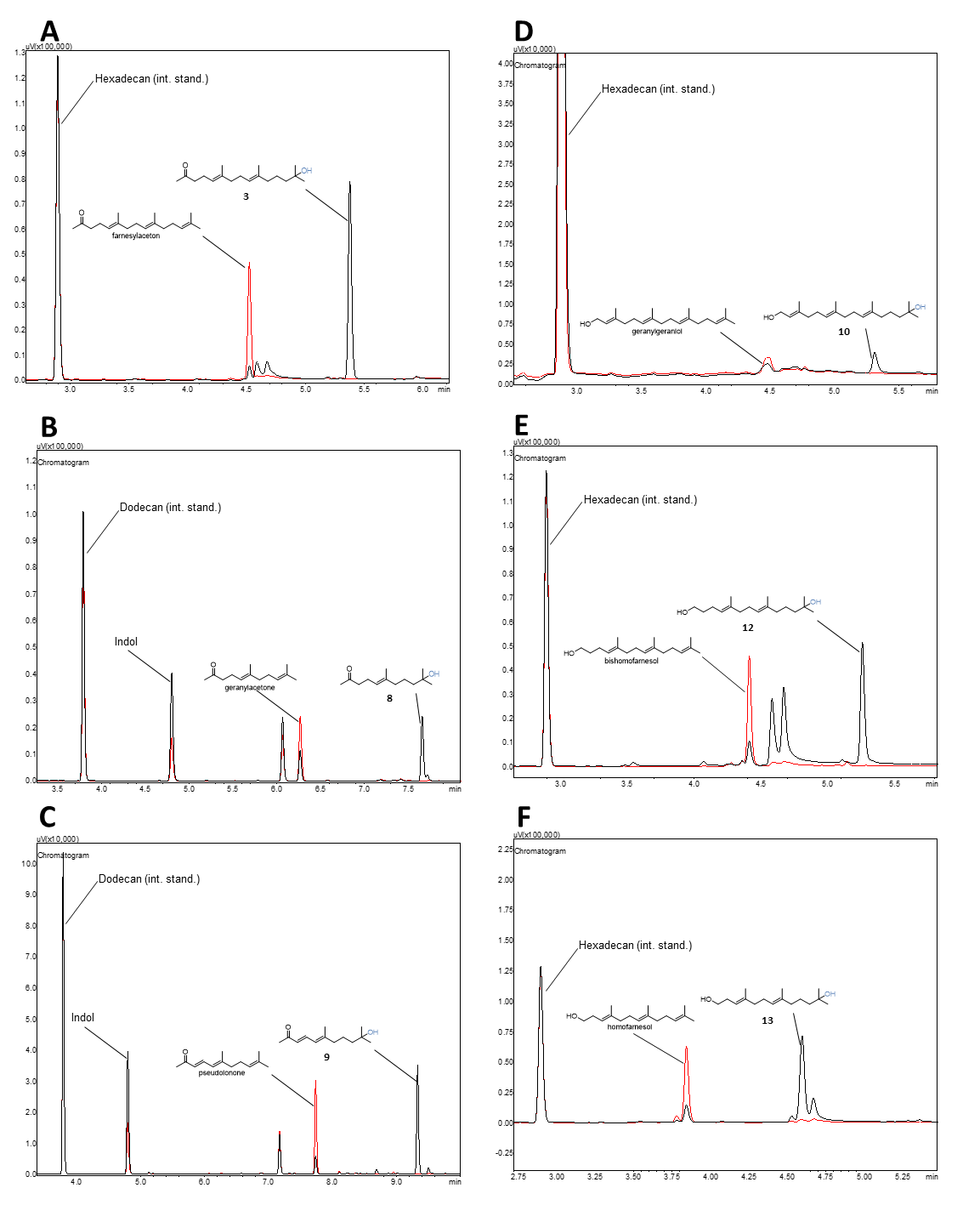


Figure S12: GC/FID chromatograms of wc-biotransformations with different substrates. Reactions took place with standard reaction conditions (100 mg_cww_/mL in KPi buffer, 50 mM pH = 7.0, 1 mM substrate, 24 h and 30 °C). Enzyme catalyzed reactions (black) and empty vector controls (red) are shown and were measured in triplicates. A) Reaction with substrate **farnesylacetone** and product **3** and internal standard hexadecane are shown. B) Reaction with substrate **geranylacetone** and product **8** and internal standard dodecane are shown. C) Reaction with substrate **pseudoionone** and product **9** and internal standard dodecane are shown. D) Reaction with substrate **geranylgeraniol** and product **10** and internal standard hexadecane are shown. E) Reaction with substrate **bishomofarnesol** and product **12** and internal standard hexadecane are shown. F) Reaction with substrate **homofarnesol** and product **13** and internal standard hexadecane are shown.


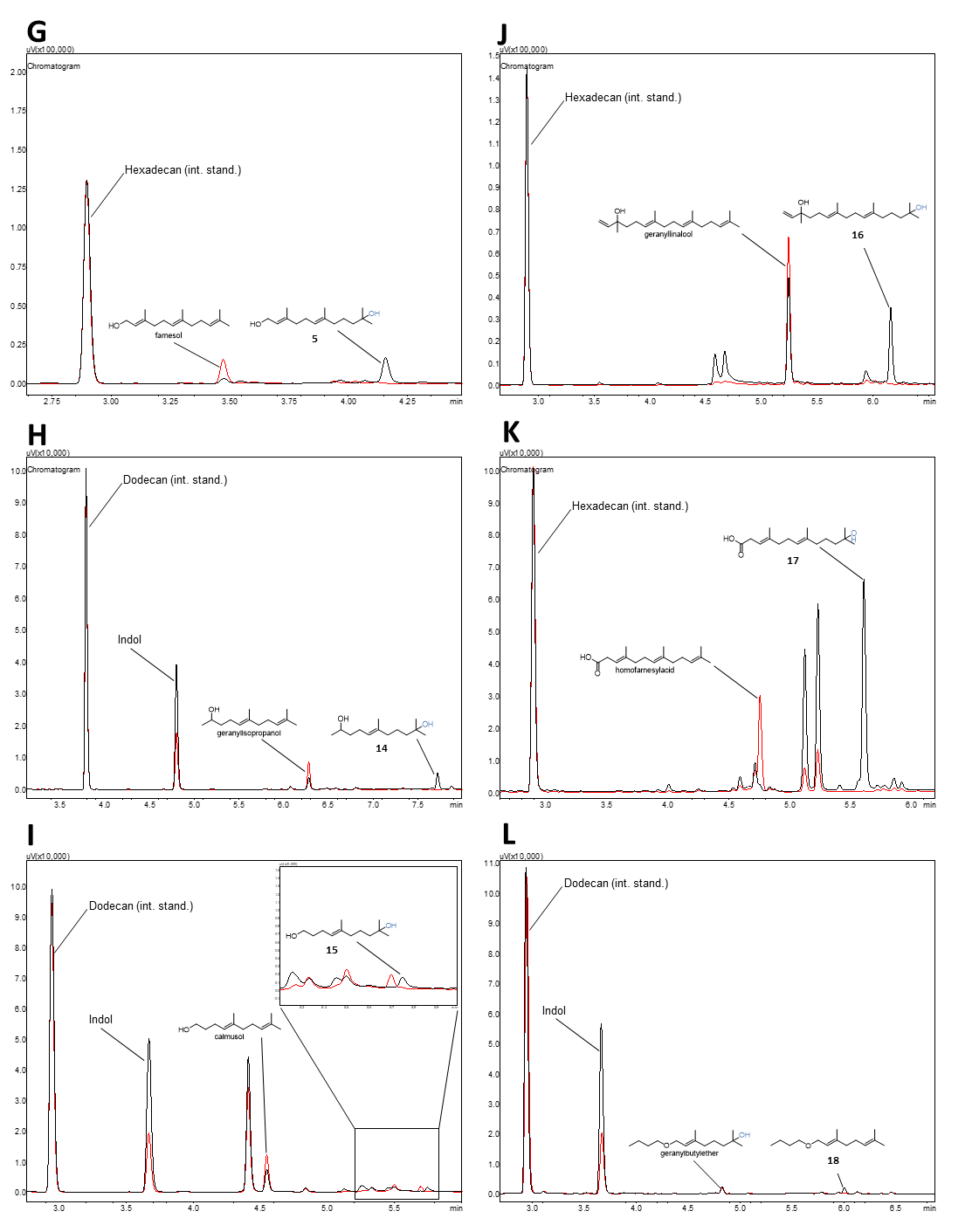


Figure S13: GC/FID chromatograms of wc-biotransformations with different substrates. Reactions took place with standard reaction conditions (100 mg_cww_/mL in KPi buffer, 50 mM pH = 7.0, 1 mM substrate, 24 h and 30 °C). Enzyme catalyzed reactions (black) and empty vector controls (red) are shown and were measured in triplicates. G) Reaction with substrate **farnesol** and product **5** and internal standard hexadecane are shown. H) Reaction with substrate **geranylisopropanol** and product **14** and internal standard dodecane are shown. I) Reaction with substrate **calmusol** and product **15** and internal standard dodecane are shown. J) Reaction with substrate **geranyllinalool** and product **16** and internal standard hexadecane are shown. K) Reaction with substrate **homofarnesylic acid** and product **17** and internal standard hexadecane are shown. L) Reaction with substrate **geranyl butyl ether** and product **18** and internal standard dodecane are shown.


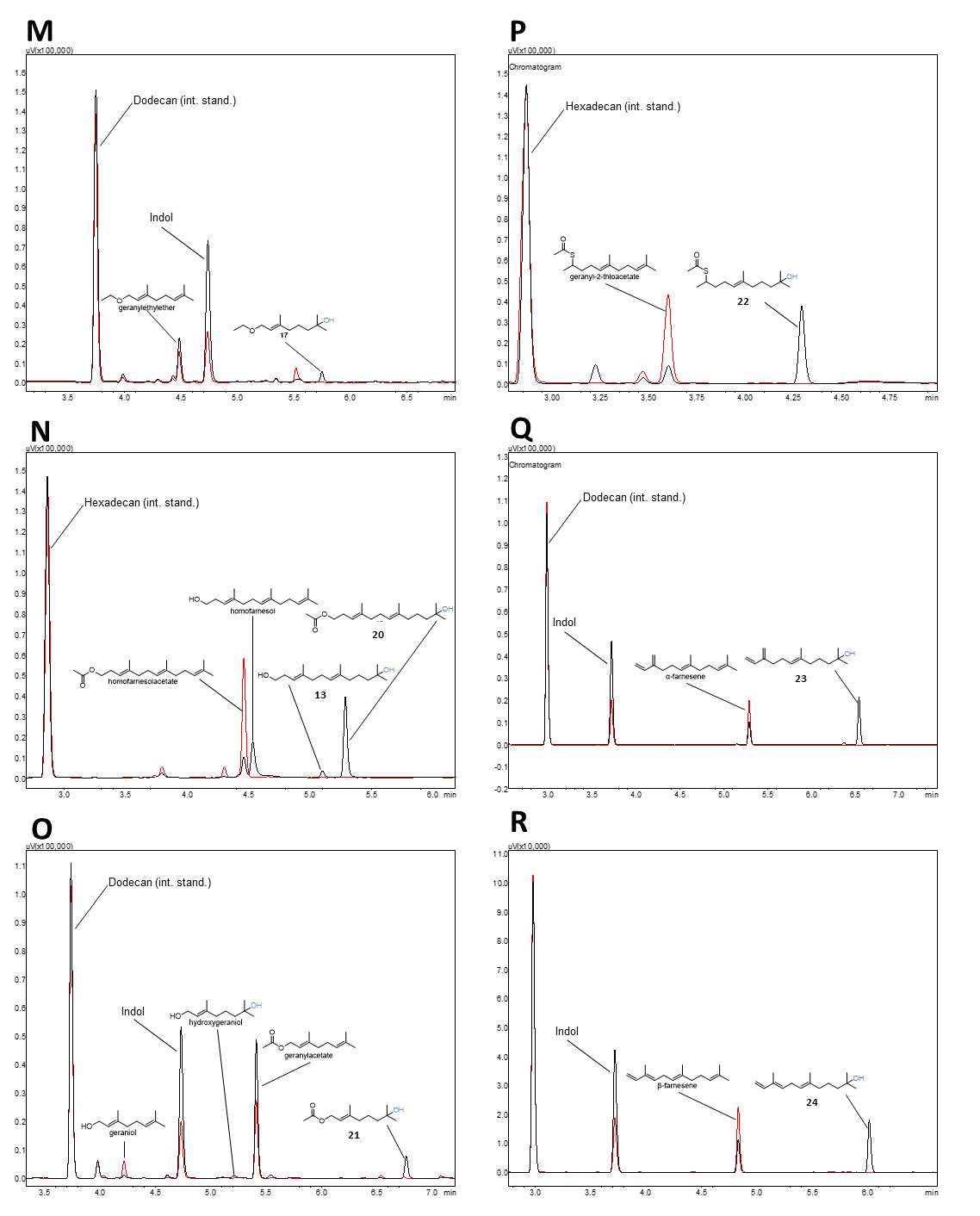


Figure S14: GC/FID chromatograms of wc-biotransformations with different substrates. Reactions took place with standard reaction conditions (100 mg_cww_/mL in KPi buffer, 50 mM pH = 7.0, 1 mM substrate, 24 h and 30 °C). Enzyme catalyzed reactions (black) and empty vector controls (red) are shown and were measured in triplicates. M) Reaction with substrate **geranyl ethyl ether** and product **17** and internal standard dodecane are shown. N) Reaction with substrate **homofarnesyl acetate** and product **20** and internal standard hexadecane are shown. O) Reaction with substrate **geranyl acetate** and product **21** and internal standard dodecane are shown. P) Reaction with substrate **geranylthio acetate** and product **22** and internal standard hexadecane are shown. Q) Reaction with substrate **α-farnesene** and product **23** and internal standard dodecane are shown. R) Reaction with substrate **β-farnesene** and product **24** and internal standard dodecane are shown.


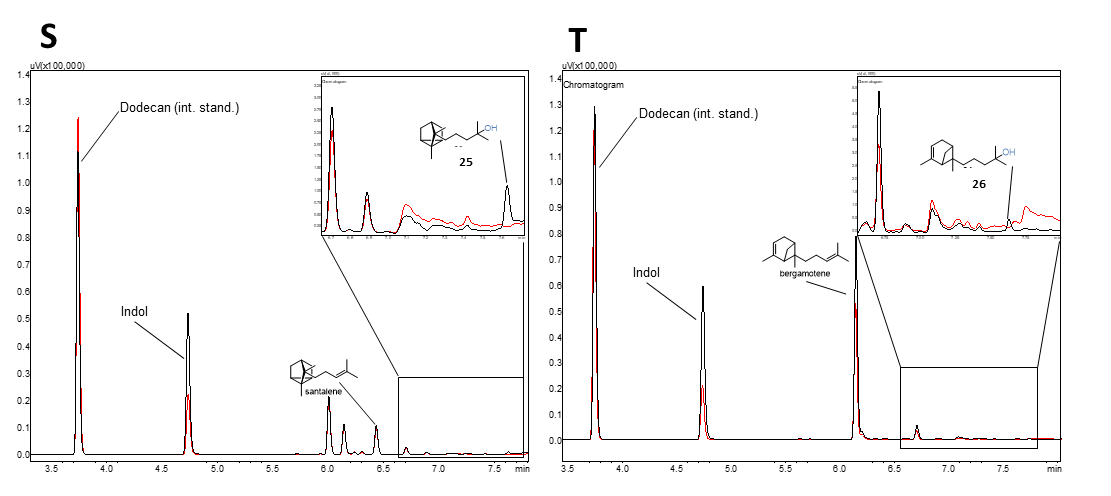


Figure S15: GC/FID chromatograms of wc-biotransformations with different substrates. Reactions took place with standard reaction conditions (100 mg_cww_/mL in KPi buffer, 50 mM pH = 7.0, 1 mM substrate, 24 h and 30 °C). Enzyme catalyzed reactions (black) and empty vector controls (red) are shown and were measured in triplicates. S) Reaction with substrate **santalene** and product **25** and internal standard dodecane are shown. T) Reaction with substrate **bergamotene** and product **26** and internal standard dodecane are shown.


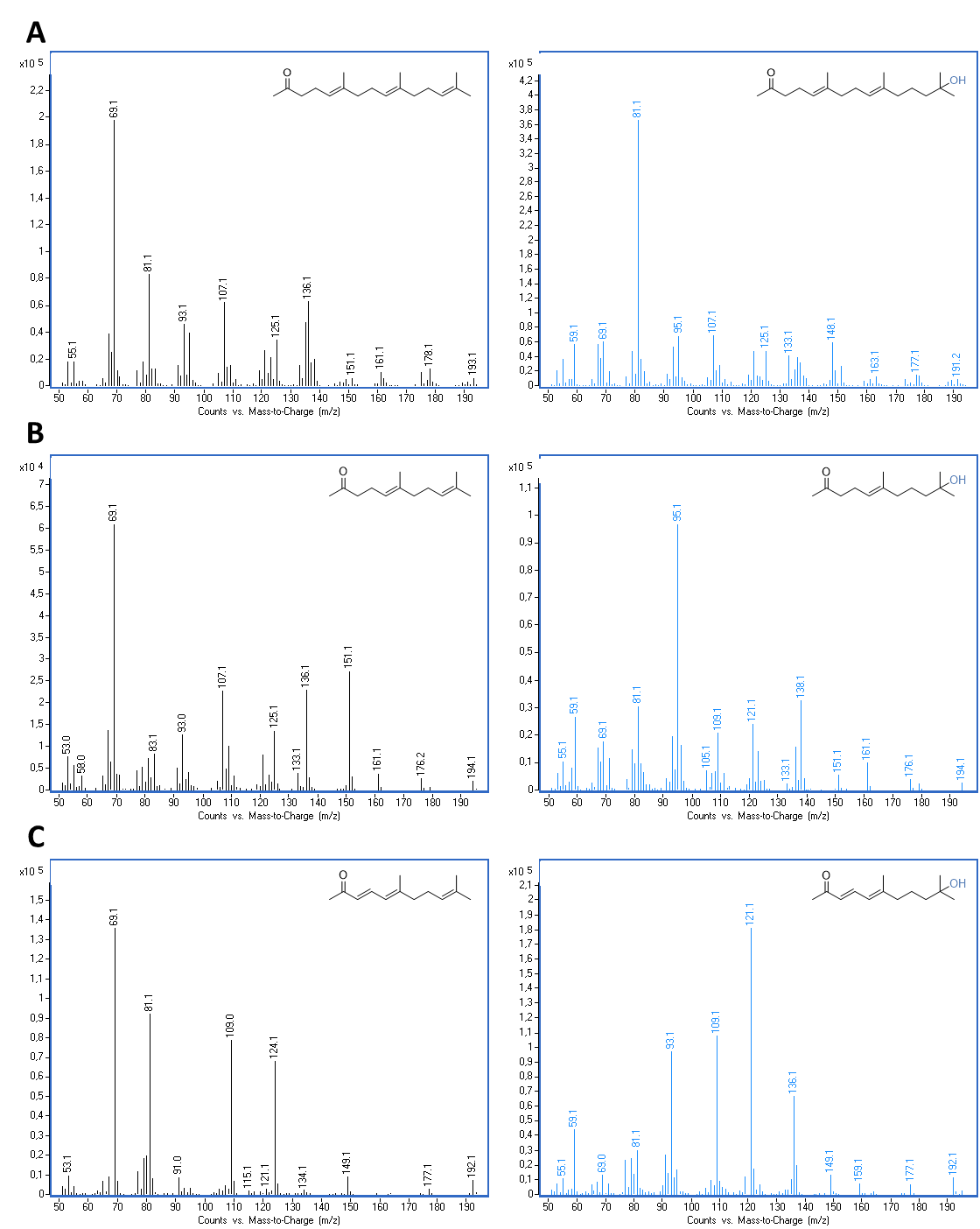


Figure S16: GC/MS fragmentation pattern of different substrates and products used in wc-biotransofrmations. Reactions took place with standard reaction conditions (100 mg_cww_/mL in KPi buffer, 50 mM pH = 7.0, 1 mM substrate, 24 h and 30 °C). Substrate fragmentation pattern (black) and product fragmentation pattern (blue) are depicted. A) Fragmentation pattern of substrate **farnesylacetone** and product **3**. B) Fragmentation pattern of substrate **geranylacetone** and product **8**. C) Fragmentation pattern of substrate **pseudoionone** and product **9**.


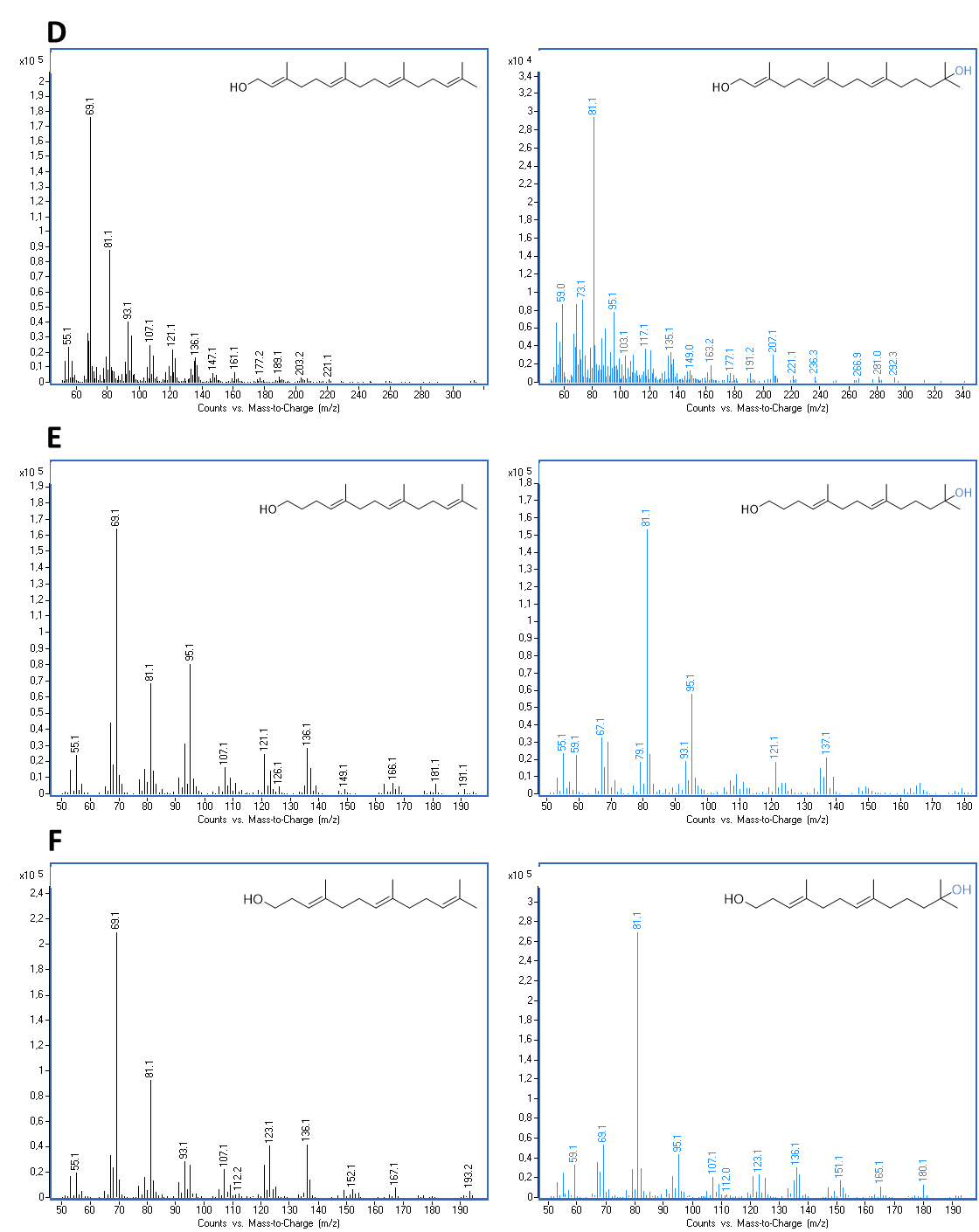


Figure S17: GC/MS fragmentation pattern of different substrates and products used in wc-biotransofrmations. Reactions took place with standard reaction conditions (100 mg_cww_/mL in KPi buffer, 50 mM pH = 7.0, 1 mM substrate, 24 h and 30 °C). Substrate fragmentation pattern (black) and product fragmentation pattern (blue) are depicted. D) Fragmentation pattern of substrate **geranylgeraniol** and product **10**. E) Fragmentation pattern of substrate **bishomofarnesol** and product **12**. F) Fragmentation pattern of substrate **homofarnesol** and product **13**.


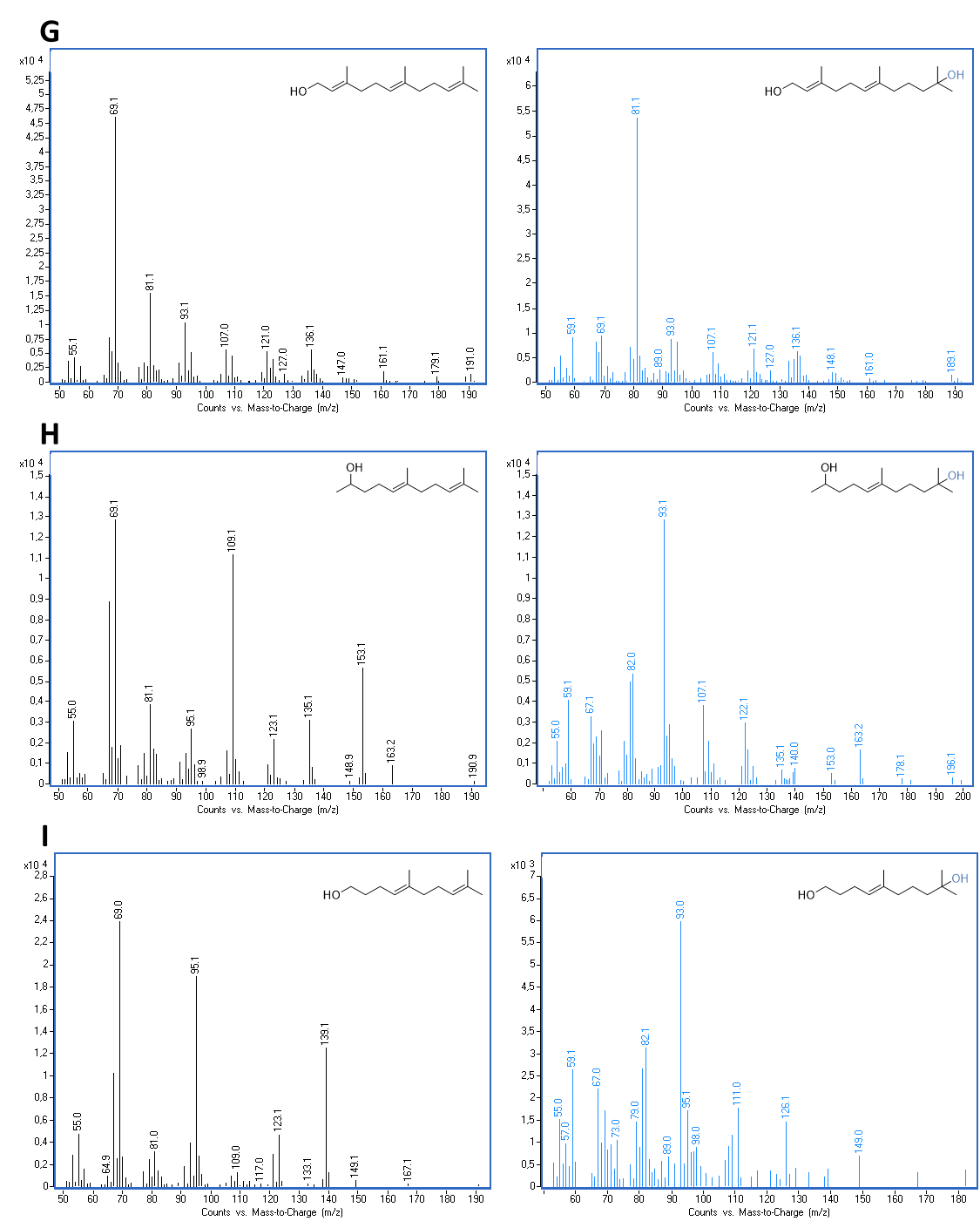


Figure S18: GC/MS fragmentation pattern of different substrates and products used in wc-biotransofrmations. Reactions took place with standard reaction conditions (100 mg_cww_/mL in KPi buffer, 50 mM pH = 7.0, 1 mM substrate, 24 h and 30 °C). Substrate fragmentation pattern (black) and product fragmentation pattern (blue) are depicted. G) Fragmentation pattern of substrate **farnesol** and product **5**. H) Fragmentation pattern of substrate **geranylisopropanol** and product **14**. I) Fragmentation pattern of substrate **calmusol** and product **15**.


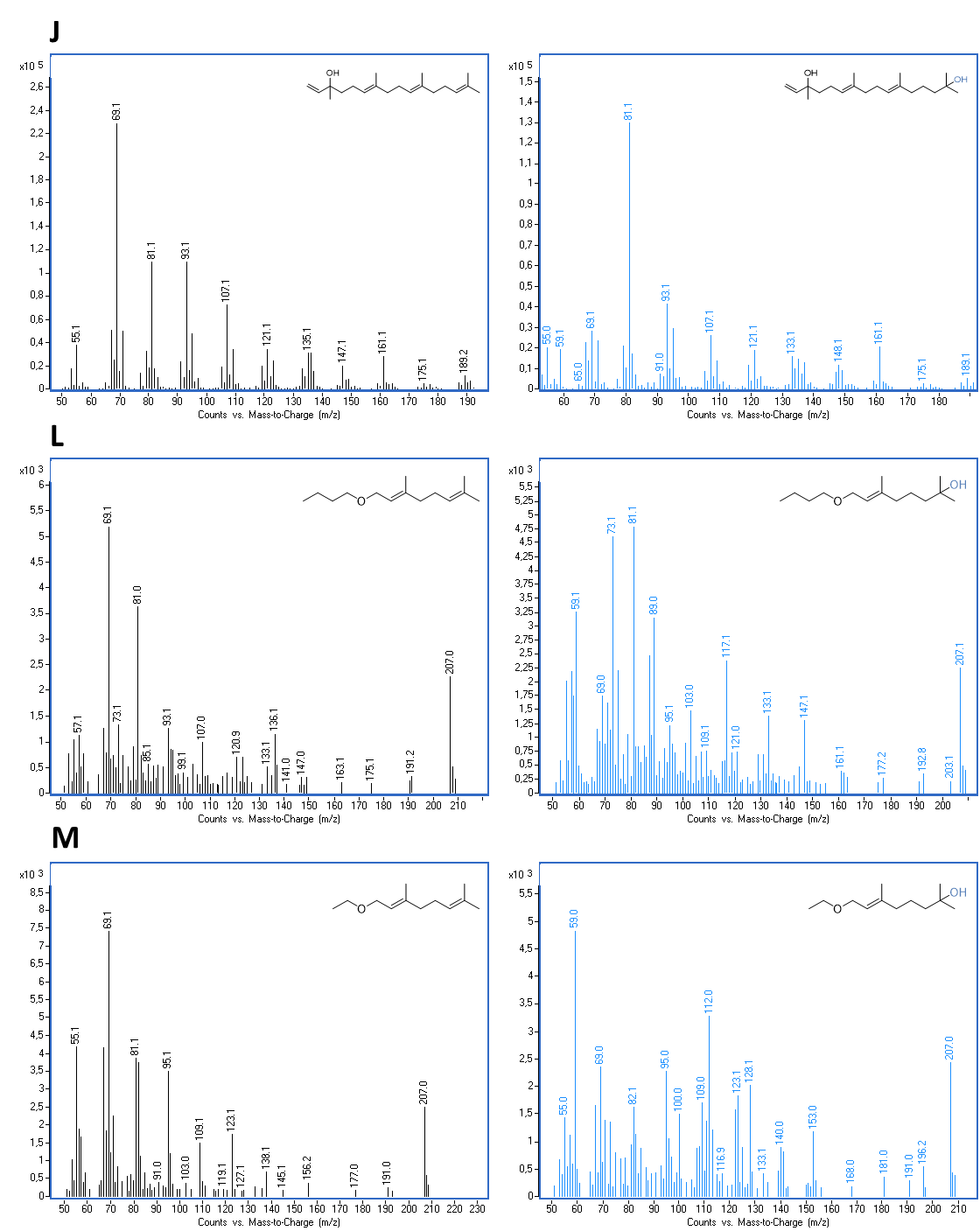


Figure S19: GC/MS fragmentation pattern of different substrates and products used in wc-biotransofrmations. Reactions took place with standard reaction conditions (100 mg_cww_/mL in KPi buffer, 50 mM pH = 7.0, 1 mM substrate, 24 h and 30 °C). Substrate fragmentation pattern (black) and product fragmentation pattern (blue) are depicted. J) Fragmentation pattern of substrate **geranyllinalool** and product **16**. K) Fragmentation pattern of substrate **geranyl butyl ether** and product **18**. L) Fragmentation pattern of substrate **geranyl ethyl ether** and product **19**.


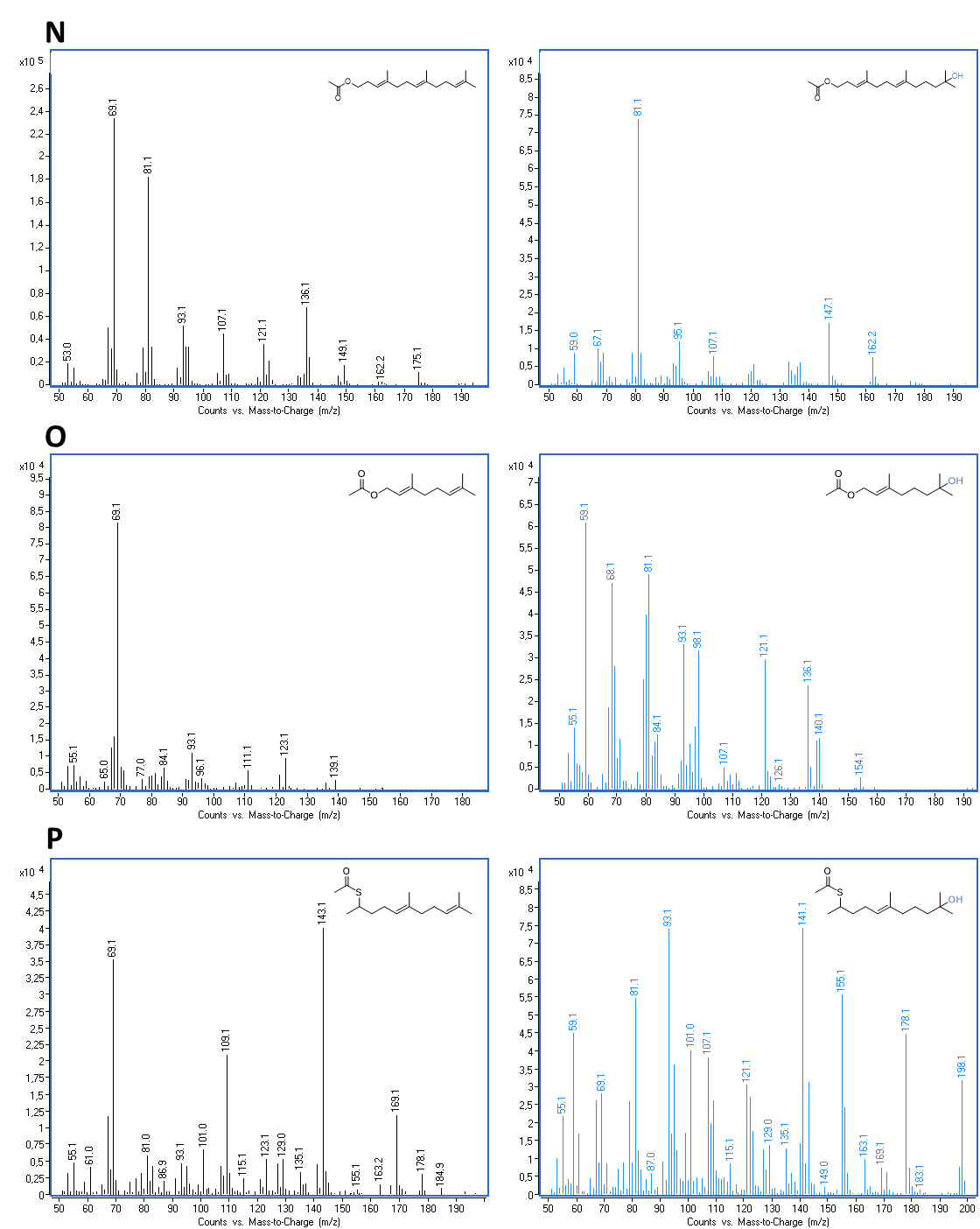


Figure S20: GC/MS fragmentation pattern of different substrates and products used in wc-biotransofrmations. Reactions took place with standard reaction conditions (100 mg_cww_/mL in KPi buffer, 50 mM pH = 7.0, 1 mM substrate, 24 h and 30 °C). Substrate fragmentation pattern (black) and product fragmentation pattern (blue) are depicted. M) Fragmentation pattern of substrate **homofarnesyl acetate** and product **20**. N) Fragmentation pattern of substrate **geranyl acetate** and product **21**. O) Fragmentation pattern of substrate **geranylthio acetate** and product **22**.


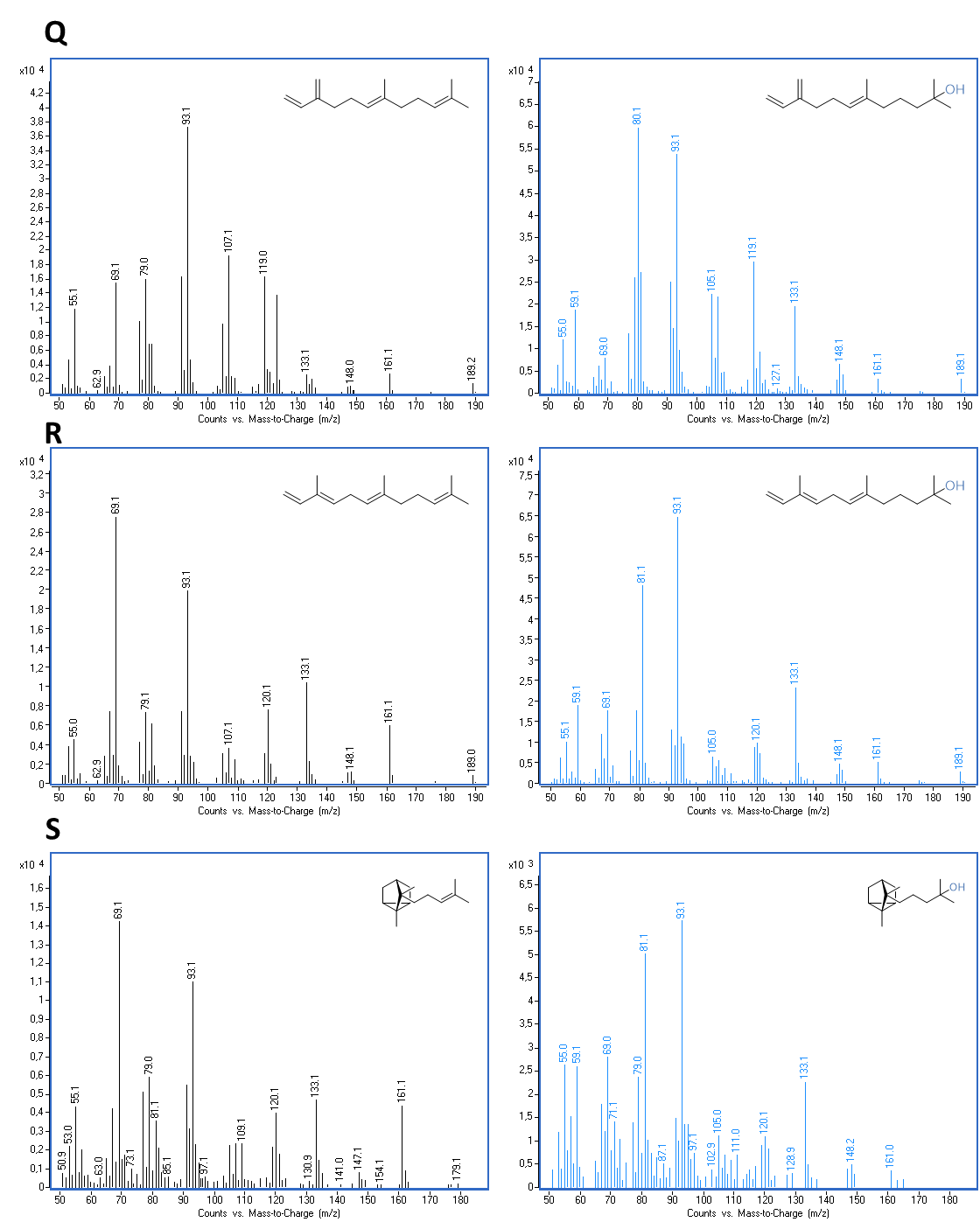


Figure S21: GC/MS fragmentation pattern of different substrates and products used in wc-biotransofrmations. Reactions took place with standard reaction conditions (100 mg_cww_/mL in KPi buffer, 50 mM pH = 7.0, 1 mM substrate, 24 h and 30 °C). Substrate fragmentation pattern (black) and product fragmentation pattern (blue) are depicted. P) Fragmentation pattern of substrate **α-farnesene** and product **23**. Q) Fragmentation pattern of substrate **β-farnesene** and product **24**. R) Fragmentation pattern of substrate **santalene** and product **25**.


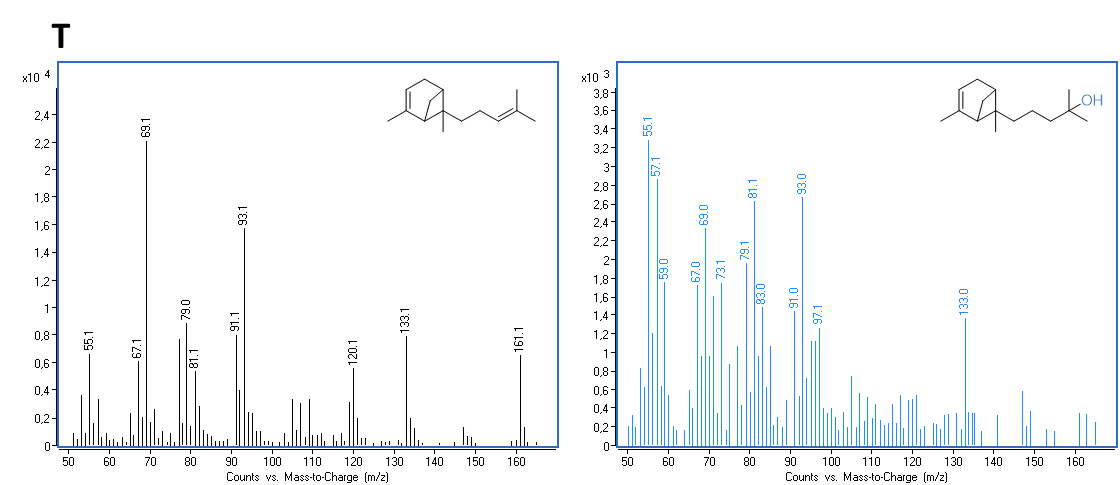


Figure S22: GC/MS fragmentation pattern of different substrates and products used in wc-biotransofrmations. Reactions took place with standard reaction conditions (100 mg_cww_/mL in KPi buffer, 50 mM pH = 7.0, 1 mM substrate, 24 h and 30 °C). Substrate fragmentation pattern (black) and product fragmentation pattern (blue) are depicted. S) Fragmentation pattern of substrate **bergamotene** and product **26**.

Figure S23: Tested substrates which were not converted under standard reaction conditions, in biotransforamtions with *Rg*CrtC IL144 WT and homology-based variants. Aldehydes: calmusal, citral (*R*)‑citronellal, (*S*)-citronellal, Ketones: 6-methyl-5-hepten-2-one. Alcohols: geraniol, (+)-isopulegol, (‑)‑isopulegol. Vinylalcohols: linalool. Non functionalized: myrcene, (+)-β-citronellen, (-)-β-citronellen, (*R*)‑limonen, (*S*)-limonen, (+)-α-pinene, (-)-α-pinene, terpinolene, sabinene.


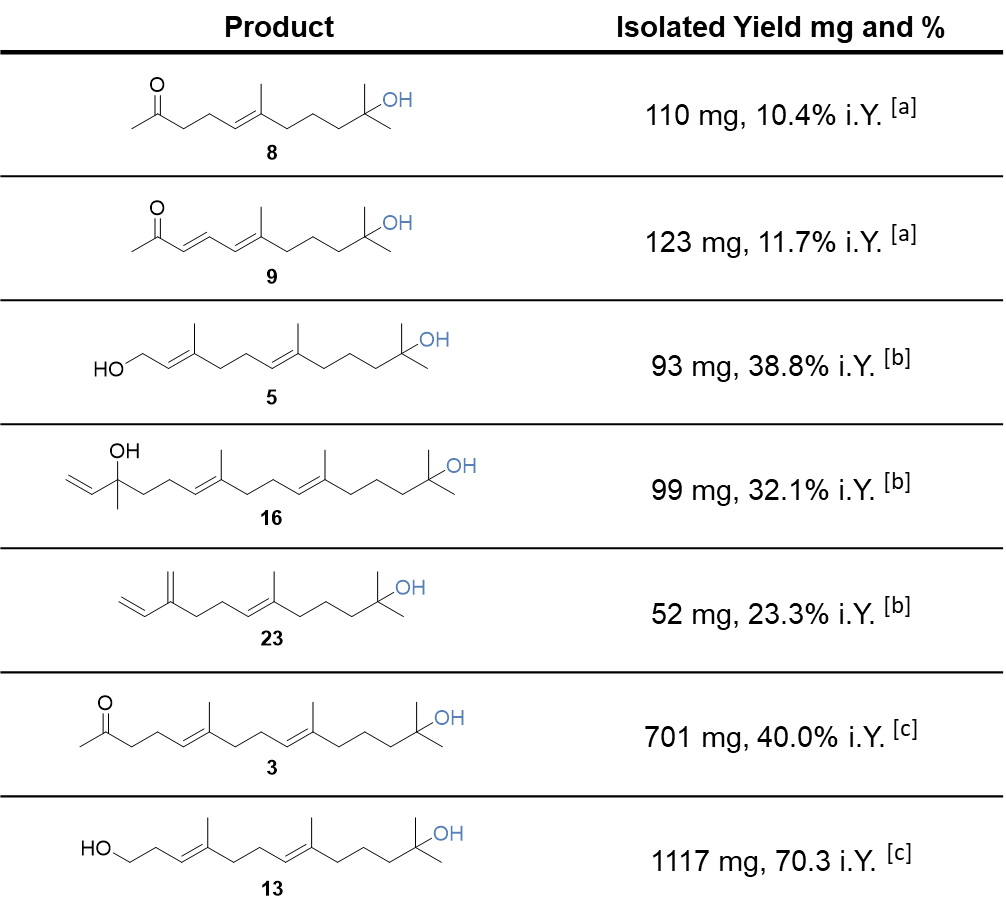


Figure S24: Isolated yields of products from preparative scale-up reactions. Reactions were conducted in 500 mL with 10 mM ^[a]^, 100 mL with 10 mM ^[b]^ or 125 mL with 50 mM ^[c]^ substrate loading. All products were isolated as yellowish oils (NMRs in section 4).5
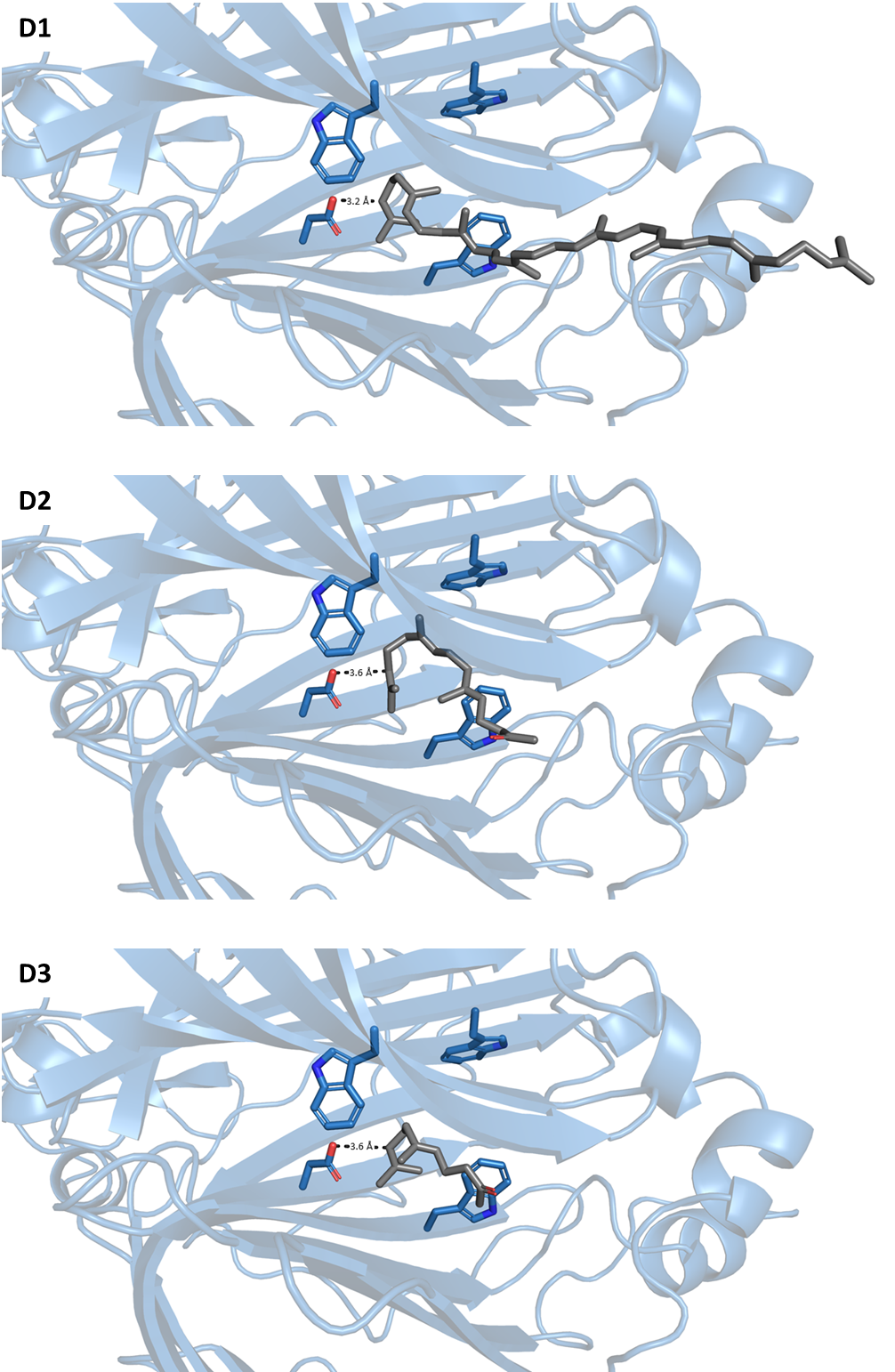


Figure S25: Docking study of the substrate scope with the alpha-fold model of *Rg*CrtC IL144. Substrates (grey, sticks) and essential catalytic amino acids D268 (blue/red, sticks), W110, W283 and W285 (blue/dark blue, sticks) are shown in the graphic. D1) Docking of the natural substrate lycopene **1**. Distance from D268 to the C2-postions of the substrate is 3.2 Å. D2) Docking of substrate farnesylaceton. Distance from D268 to the C2-postions of the substrate is 3.6 Å. D3) Docking of substrate geranylacetone. Distance from D268 to the C2-postions of the substrate is 3.6 Å.


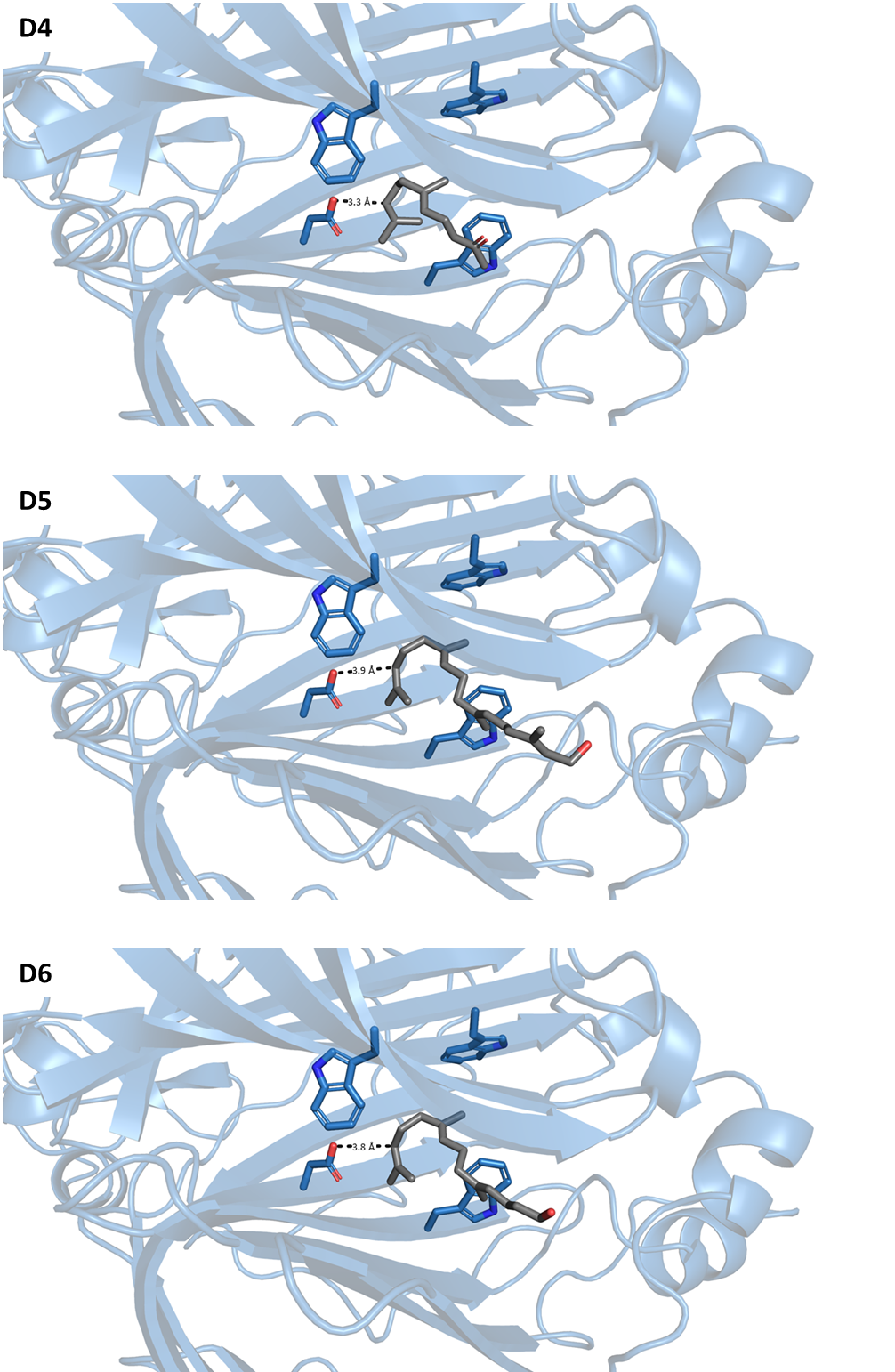


Figure S26: Docking study of the substrate scope with the alpha-fold model of *Rg*CrtC IL144. Substrates (grey, sticks) and essential catalytic amino acids D268 (blue/red, sticks), W110, W283 and W285 (blue/dark blue, sticks) are shown in the graphic. D4) Docking of substrate pseudoionone. Distance from D268 to the C2-postions of the substrate is 3.3 Å. D5) Docking of substrate geranylgeraniol. Distance from D268 to the C2-postions of the substrate is 3.9 Å. D6) Docking of substrate bishomofarnesol. Distance from D268 to the C2-postions of the substrate is 3.8 Å.


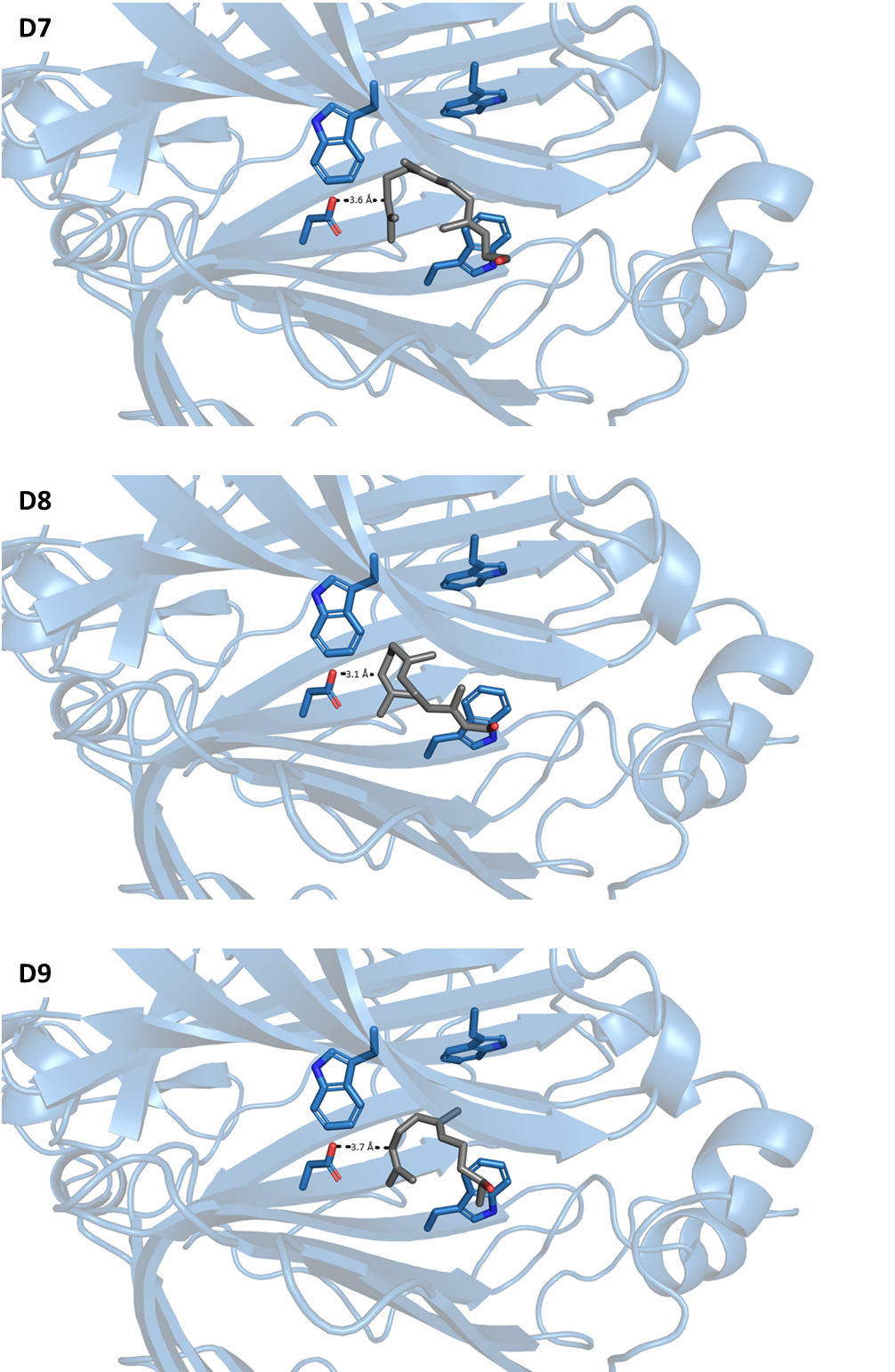


Figure S27: Docking study of the substrate scope with the alpha-fold model of *Rg*CrtC IL144. Substrates (grey, sticks) and essential catalytic amino acids D268 (blue/red, sticks), W110, W283 and W285 (blue/dark blue, sticks) are shown in the graphic. D7) Docking of substrate homofarnesol. Distance from D268 to the C2-postions of the substrate is 3.6 Å. D8) Docking of substrate farnesol. Distance from D268 to the C2-postions of the substrate is 3.1 Å. D9) Docking of substrate geranylisopropanol. Distance from D268 to the C2-postions of the substrate is 3.7 Å.


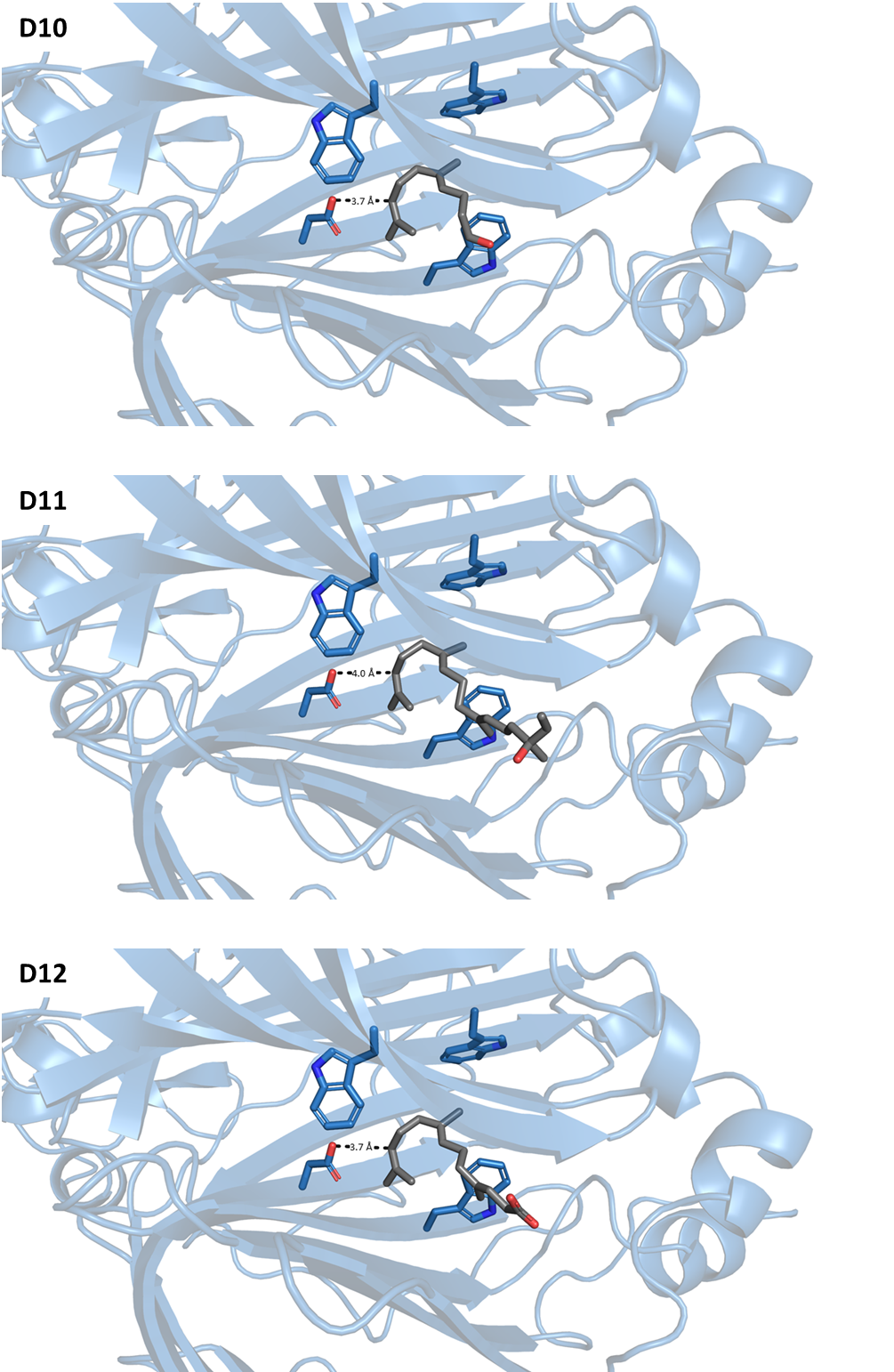


Figure S28: Docking study of the substrate scope with the alpha-fold model of *Rg*CrtC IL144. Substrates (grey, sticks) and essential catalytic amino acids D268 (blue/red, sticks), W110, W283 and W285 (blue/dark blue, sticks) are shown in the graphic. D10) Docking of substrate calmusol. Distance from D268 to the C2-postions of the substrate is 3.7 Å. D11) Docking of substrate geranyllinalool. Distance from D268 to the C2-postions of the substrate is 4.0 Å. D12) Docking of substrate homofanesylic acid. Distance from D268 to the C2-postions of the substrate is 3.7 Å.


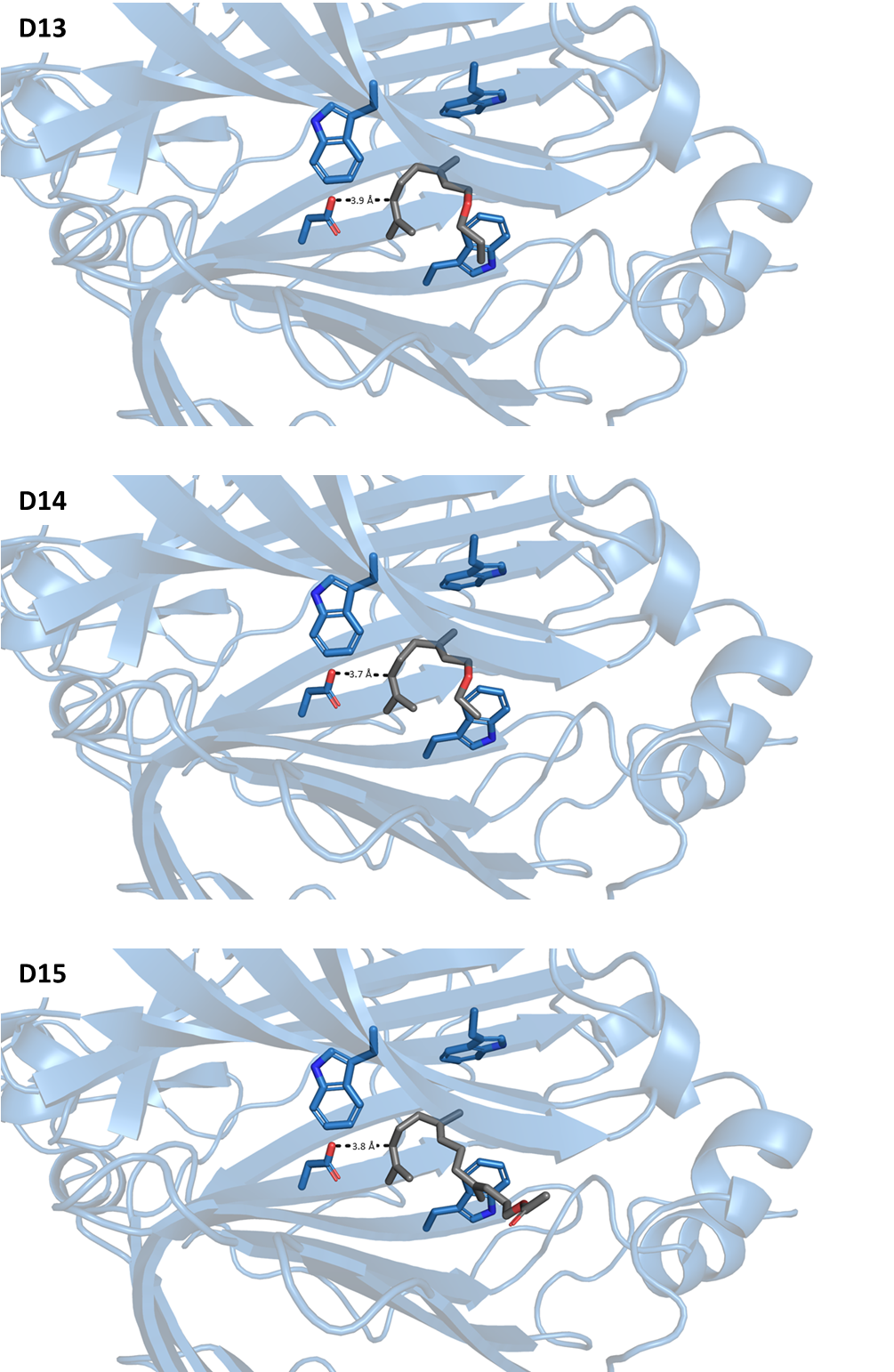


Figure S29: Docking study of the substrate scope with the alpha-fold model of *Rg*CrtC IL144. Substrates (grey, sticks) and essential catalytic amino acids D268 (blue/red, sticks), W110, W283 and W285 (blue/dark blue, sticks) are shown in the graphic. D13) Docking of substrate geranyl butyl ether. Distance from D268 to the C2-postions of the substrate is 3.9 Å. D14) Docking of substrate geranyl ethyl ether. Distance from D268 to the C2-postions of the substrate is 3.7 Å. D15) Docking of substrate homofarnesyl acetate. Distance from D268 to the C2-postions of the substrate is 3.8 Å.


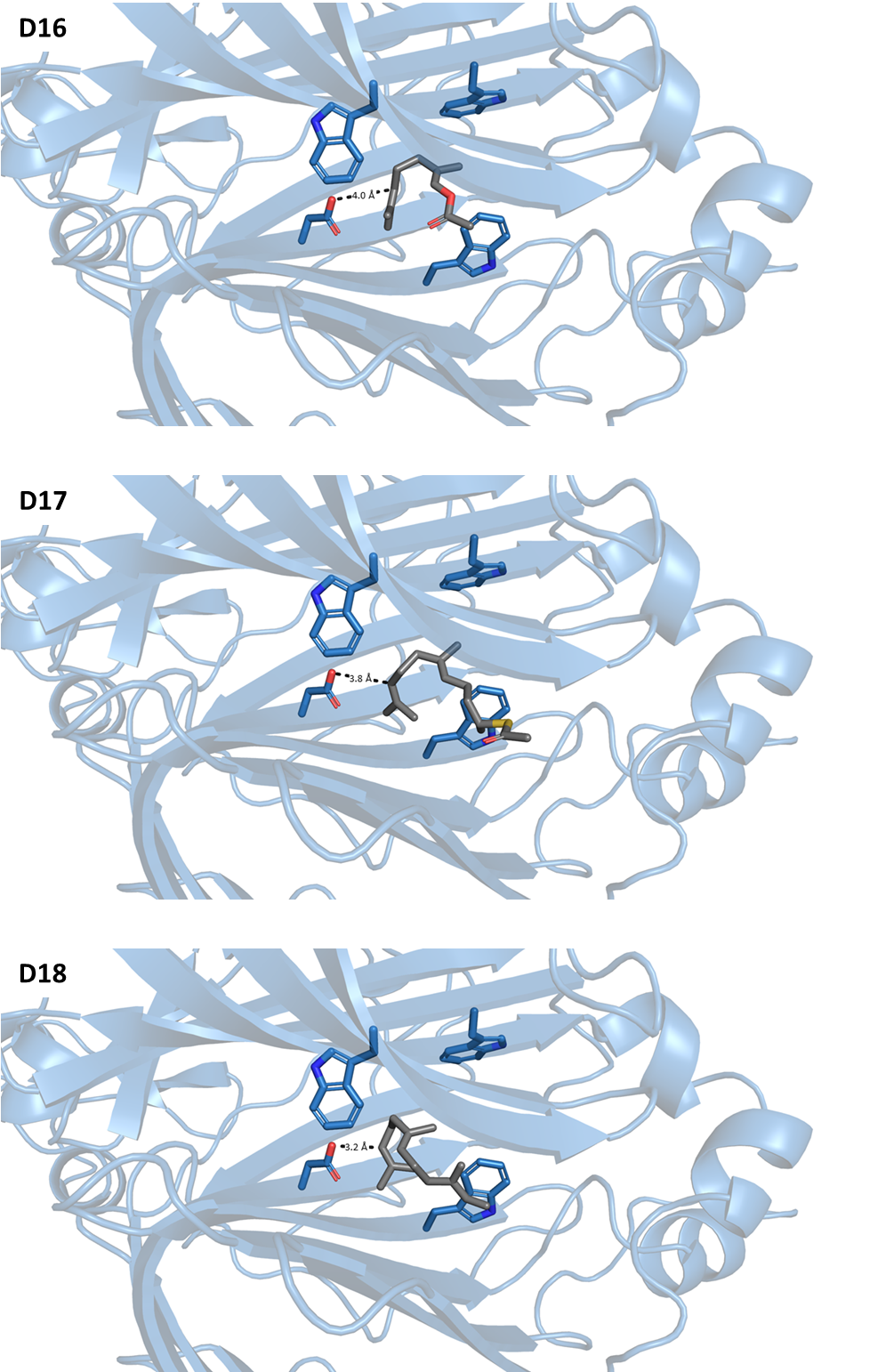


Figure S30: Docking study of the substrate scope with the alpha-fold model of *Rg*CrtC IL144. Substrates (grey, sticks) and essential catalytic amino acids D268 (blue/red, sticks), W110, W283 and W285 (blue/dark blue, sticks) are shown in the graphic. D16) Docking of substrate geranyl acetate. Distance from D268 to the C2-postions of the substrate is 4.0 Å. D17) Docking of substrate geranylthio acetate. Distance from D268 to the C2-postions of the substrate is 3.8 Å. D18) Docking of substrate α-farnesene. Distance from D268 to the C2-postions of the substrate is 3.2 Å.


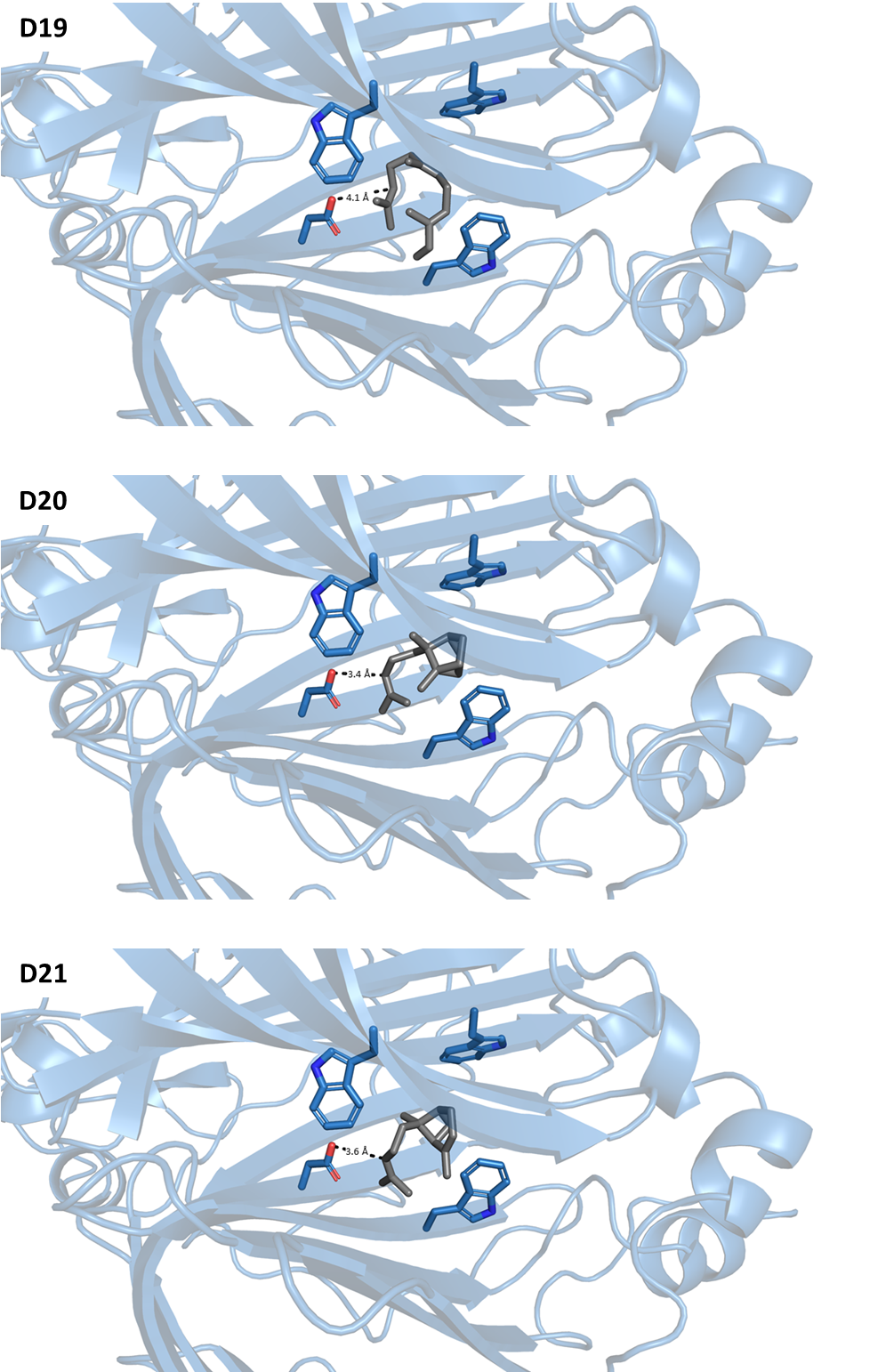


Figure S31: Docking study of the substrate scope with the alpha-fold model of *Rg*CrtC IL144. Substrates (grey, sticks) and essential catalytic amino acids D268 (blue/red, sticks), W110, W283 and W285 (blue/dark blue, sticks) are shown in the graphic. D19) Docking of substrate β-farnesene. Distance from D268 to the C2-postions of the substrate is 4.1 Å. D20) Docking of substrate santalene. Distance from D268 to the C2-postions of the substrate is 3.4 Å. D21) Docking of substrate bergamotene. Distance from D268 to the C2-postions of the substrate is 3.6 Å.


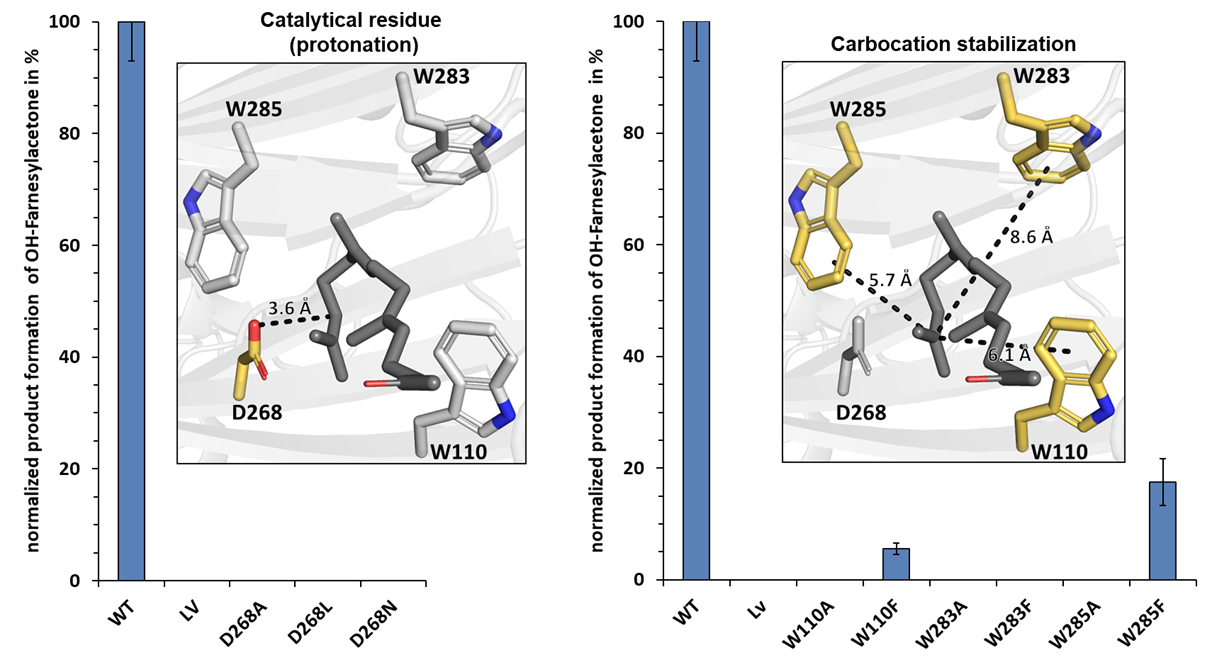


Figure S 32: Investigations of key catalytic residues were performed for the catalytic residue D268 (protonation) and the putative carbocation-stabilizing residues W110, W283 and W285 with the model substrate 8 under standard reaction conditions by amino acid substitution.


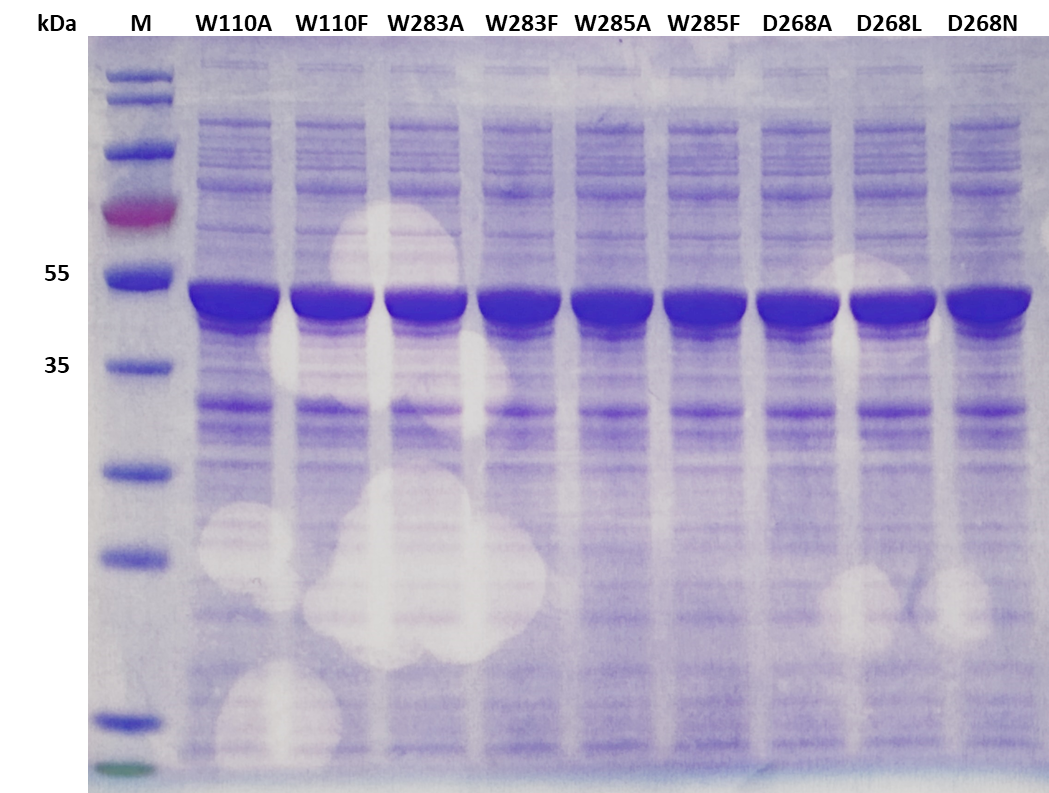


Figure S33: Investigation of crucial activity site amino acids. The expected molecular weight of the hydratase variants is 47kDa. All knock out variants showed good overexpression. Uncropped SDS-PAGE is shown in figure S41.

| **Position in *Rg*CrtC_IL144** | | | | | **Sequence frequency in MSA** |
| --- | --- | --- | --- | --- | --- |
| **131** | **132** | **133** | **134** | **135** |  |
| G | S | V | F | S | 63,41 % |
| G | S | L | F | S | 3,30 % |
| G | T | V | F | S | 2,98 % |
| G | **N** | V | F | S | 1,21 % |
| G | N | P | F | S | 0,97 % |
| G | **C** | V | F | S | 0,97 % |
| G | S | C | F | S | 0,64 % |
| G/A | L | P | F | S | 0,52 % |
| S | S | **F** | **V** | L/D/S | 0,28 % |
| G | F | P | F | G | 0,24 % |
| G | F | A | F | S | 0,20 % |
| G | A | V | F | S | 0,20 % |
| G | S | P | F | S | 0,16 % |
| G | **M** | **P** | **M** | S | 0,12 % |
| G | V | P | M | S | 0,12 % |
| G | **C** | **P** | **M** | S | 0,08 % |
| S | F | L | F | S | 0,08 % |
| N | F | I | F | S | 0,08 % |
| G | S | I | F | S | 0,08 % |
| G | Y | V | F | S | 0,04 % |
| G | **N** | **Y** | **C** | S | 0,04 % |
| G | S | S | F | S | 0,04 % |
| G | G | V | F | S | 0,04 % |
| G | S | A | F | S | 0,04 % |

Figure S34: Homology-based mutagenesis positions to generate variants for the conversion of monoterpenes. Frequency of sequences occurring in the MSA and corresponding positions in RgCrtC_IL144. The top row contains the wild-type sequence of RgCrtC_IL144. The amino acids that have been substituted for the homology-based approach in RgCrtC_IL144 are highlighted in light blue.


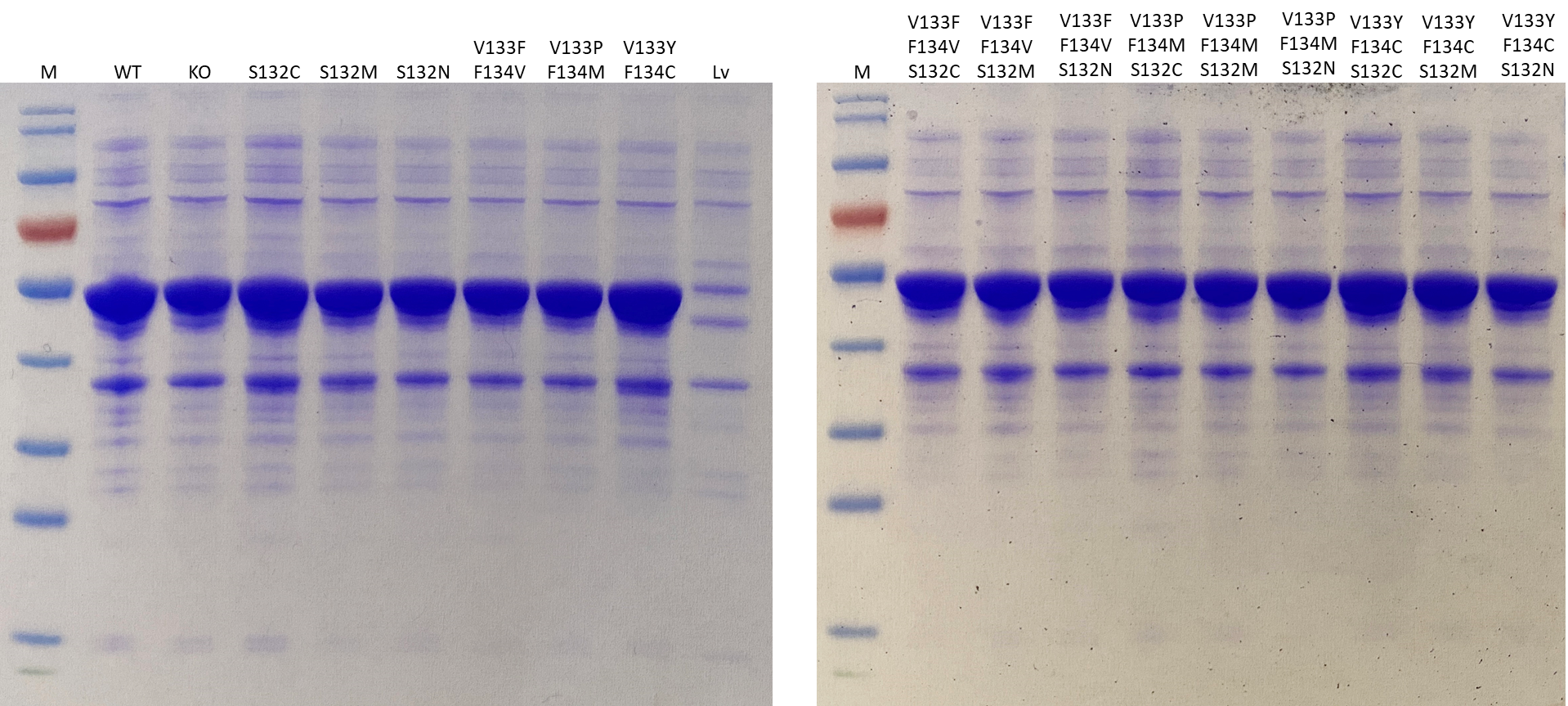


Figure S35: SDS-PAGEs of overexpressed Wild type *Rg*CrtC IL144, KO (D268A), empty Vector and the 15 homology-based mutagenesis variants. The expected molecular weight of the hydratases is 47kDa. All variants showed good overexpression. Uncropped SDS-PAGEs are shown in figure S42 and S43.


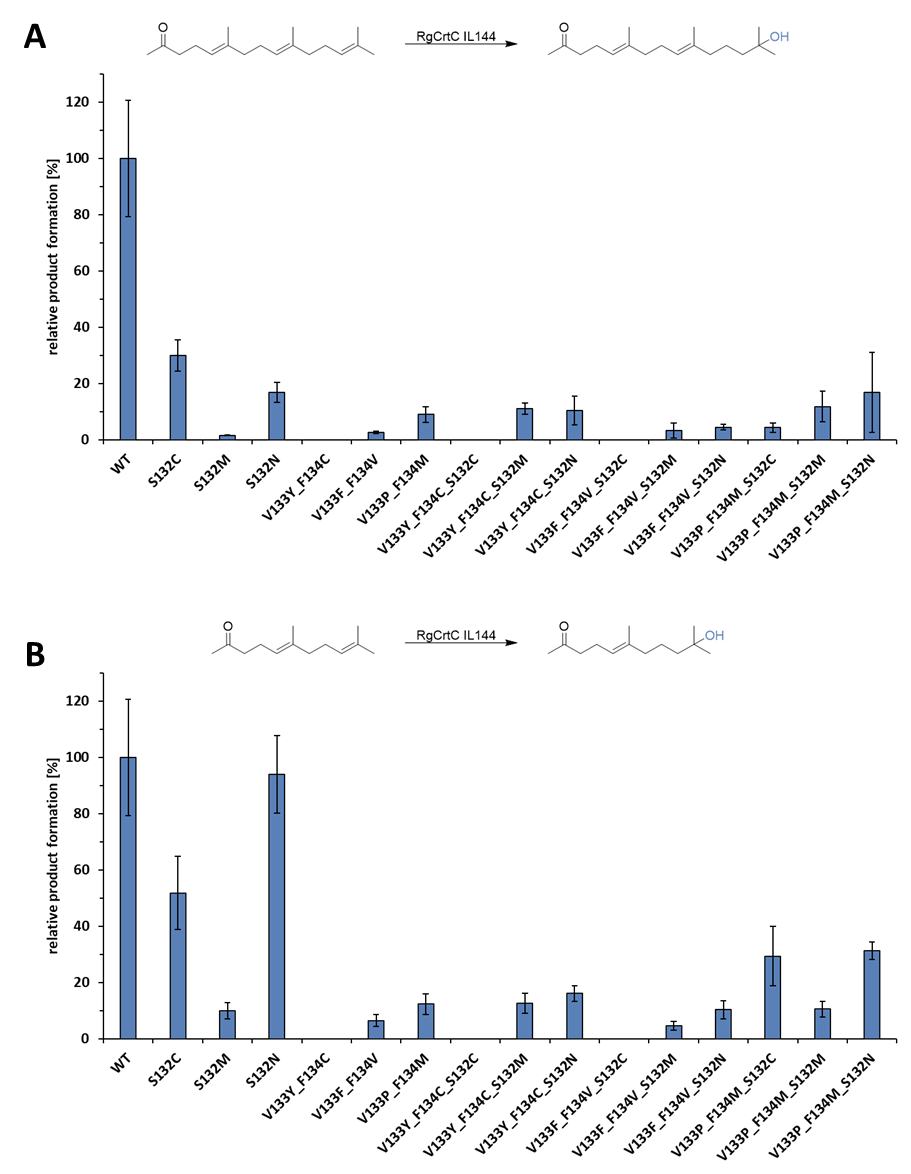


Figure S36: Biotransformations with homology-based mutagenesis variants to increase hydration activity of *Rg*CrtC IL144. Reactions were conducted in 96 DWPs in KPi buffer, 50 mM pH = 7.0, 1 mM substrate for 5 h at 30 °C. The reactions were tested with A) **2** and B) **6** as substrates in triplicates. The relative product formation of **3** and **8** was analyzed.


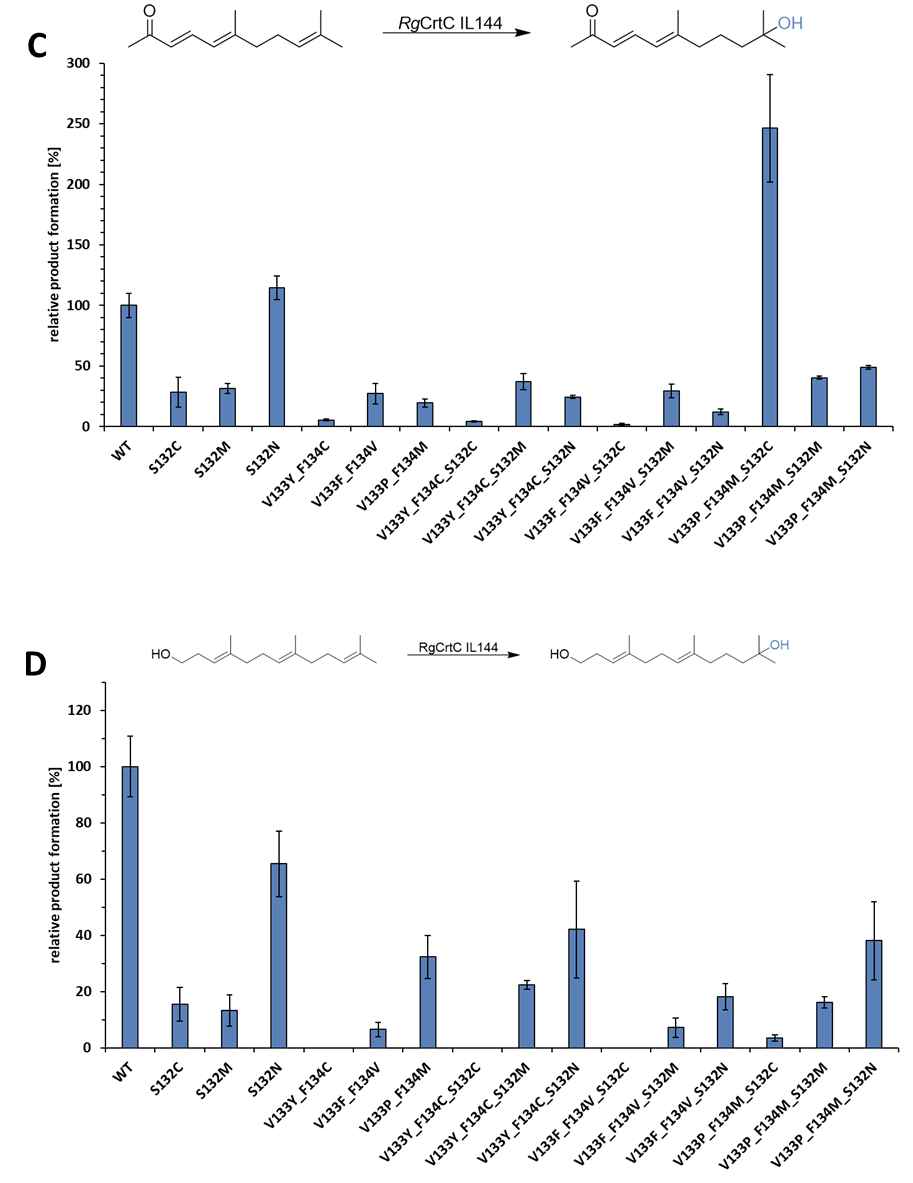


Figure S37: Biotransformations with homology-based mutagenesis variants to increase hydration activity of *Rg*CrtC IL144. Reactions were conducted in 96 DWPs in KPi buffer, 50 mM pH = 7.0, 1 mM substrate for 5 h at 30 °C. The reactions were tested with C) **7** and D) **11** as substrates in triplicates. The relative product formation of **9** and **13** was analyzed.


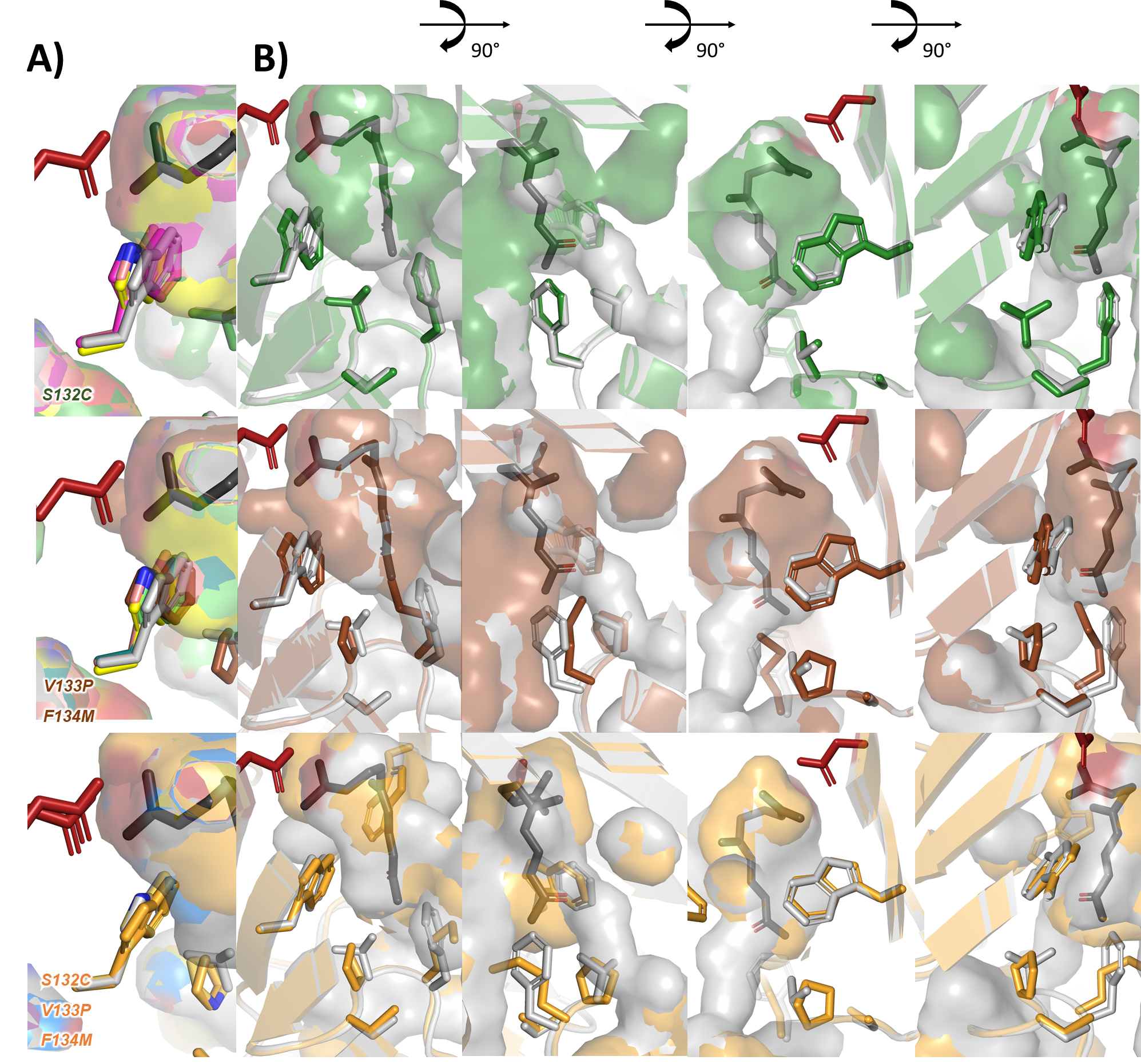


Figure S38: A) Structural overlay of generated alpha fold models (n=5) of the variants S132C (green), V133P_F134M (brown) and S132C_V133P_F134M (orange) and their comparison with the wildtype (grey).
B) Stereo view of structural overlay.


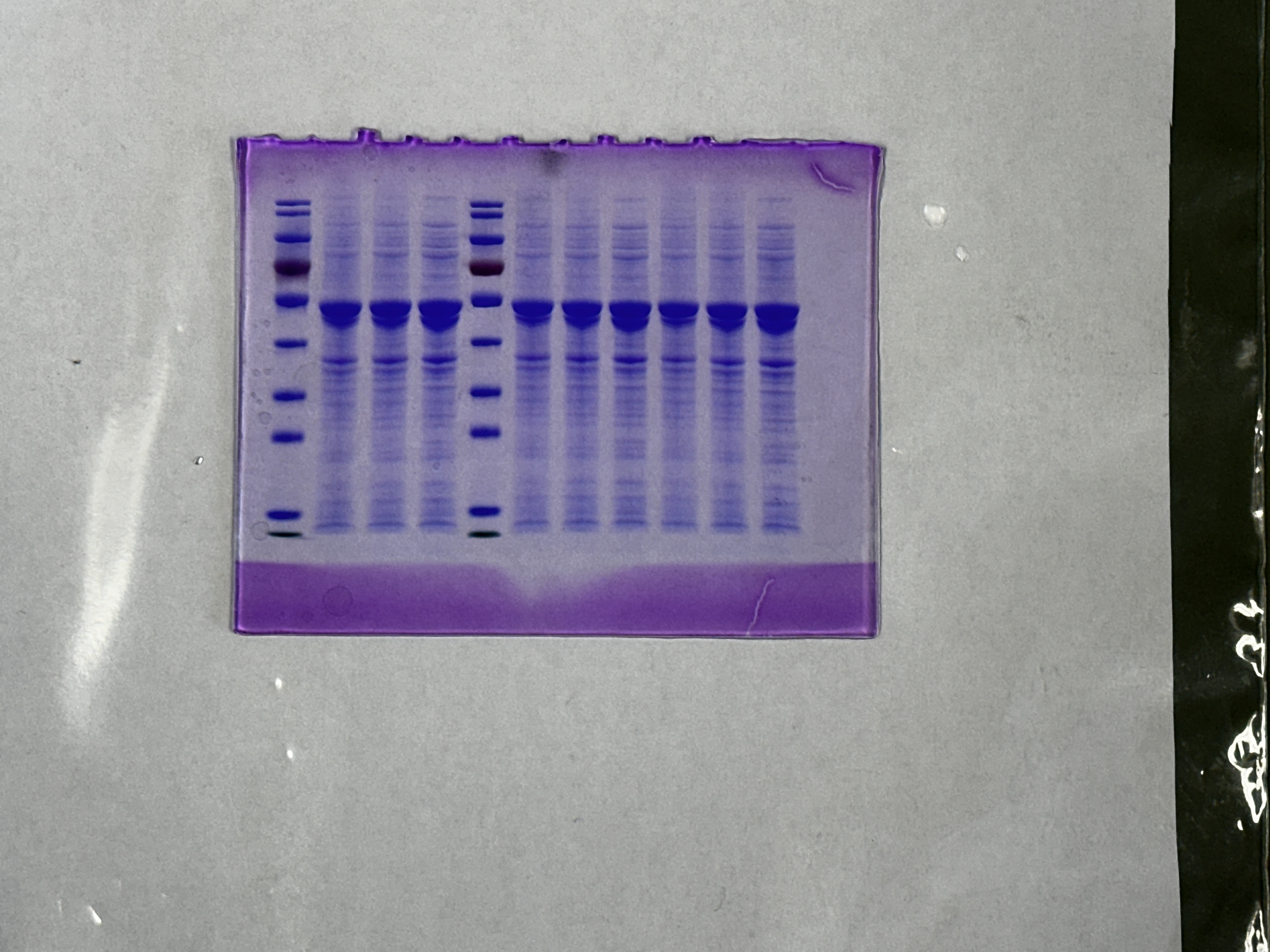


Figure S39: Uncropped SDS-PAGE for Figure S5A and Figure S8A.


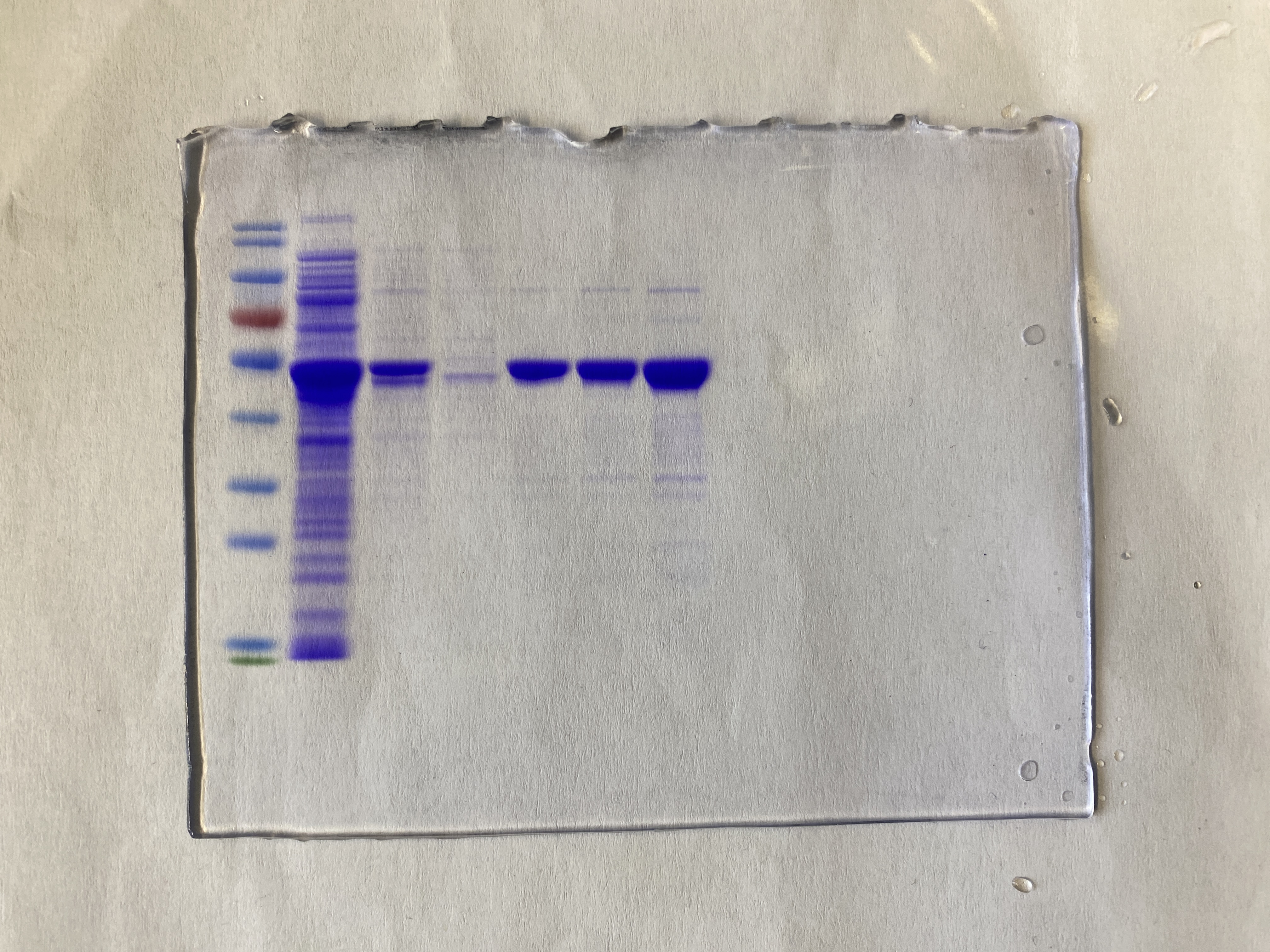


Figure S40: Uncropped SDS-PAGE for Figure S11A.


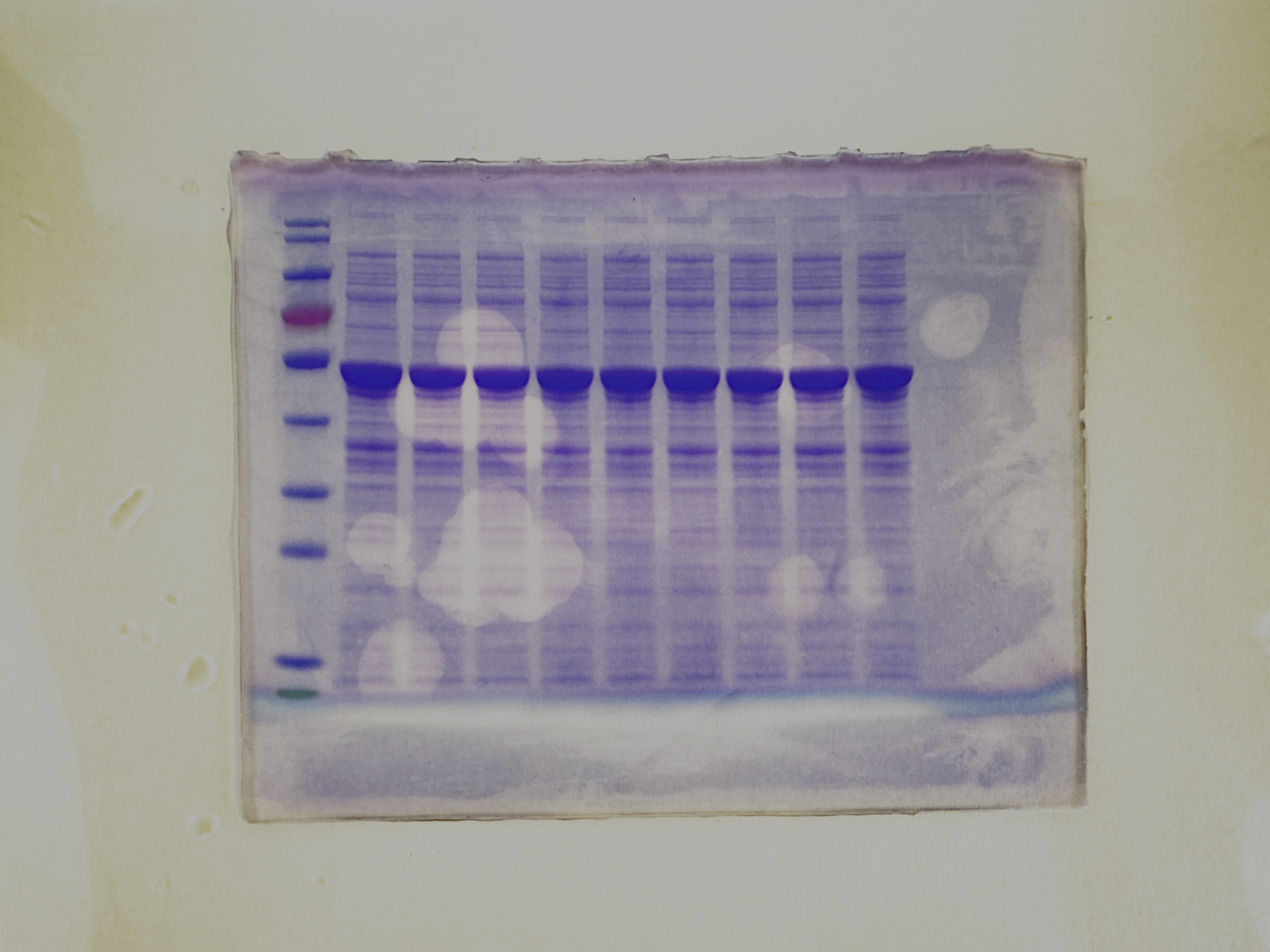


Figure S41: Uncropped SDS-PAGE for Figure S33.


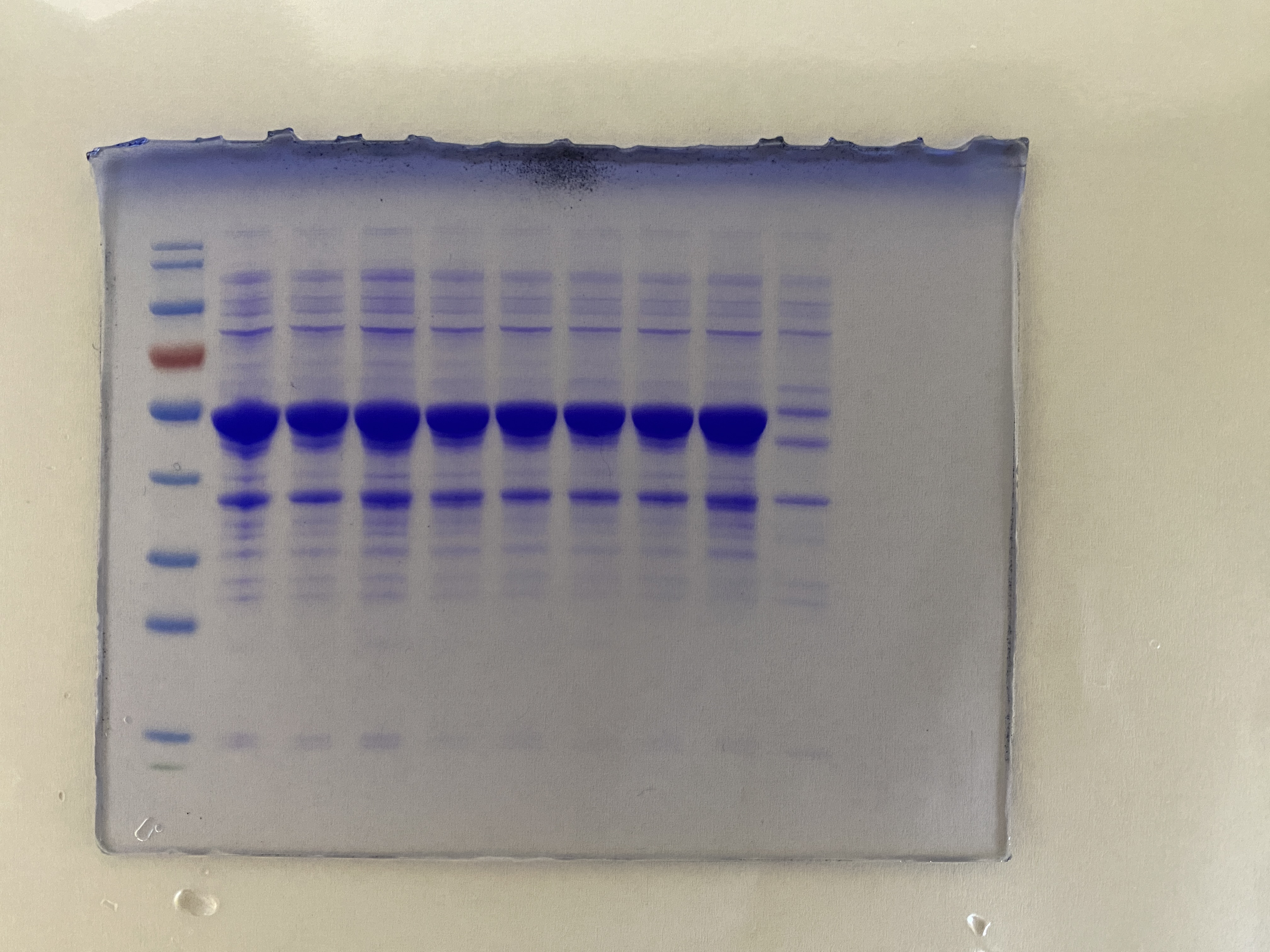


Figure S42: Uncropped SDS-PAGE for Figure S35 (left).


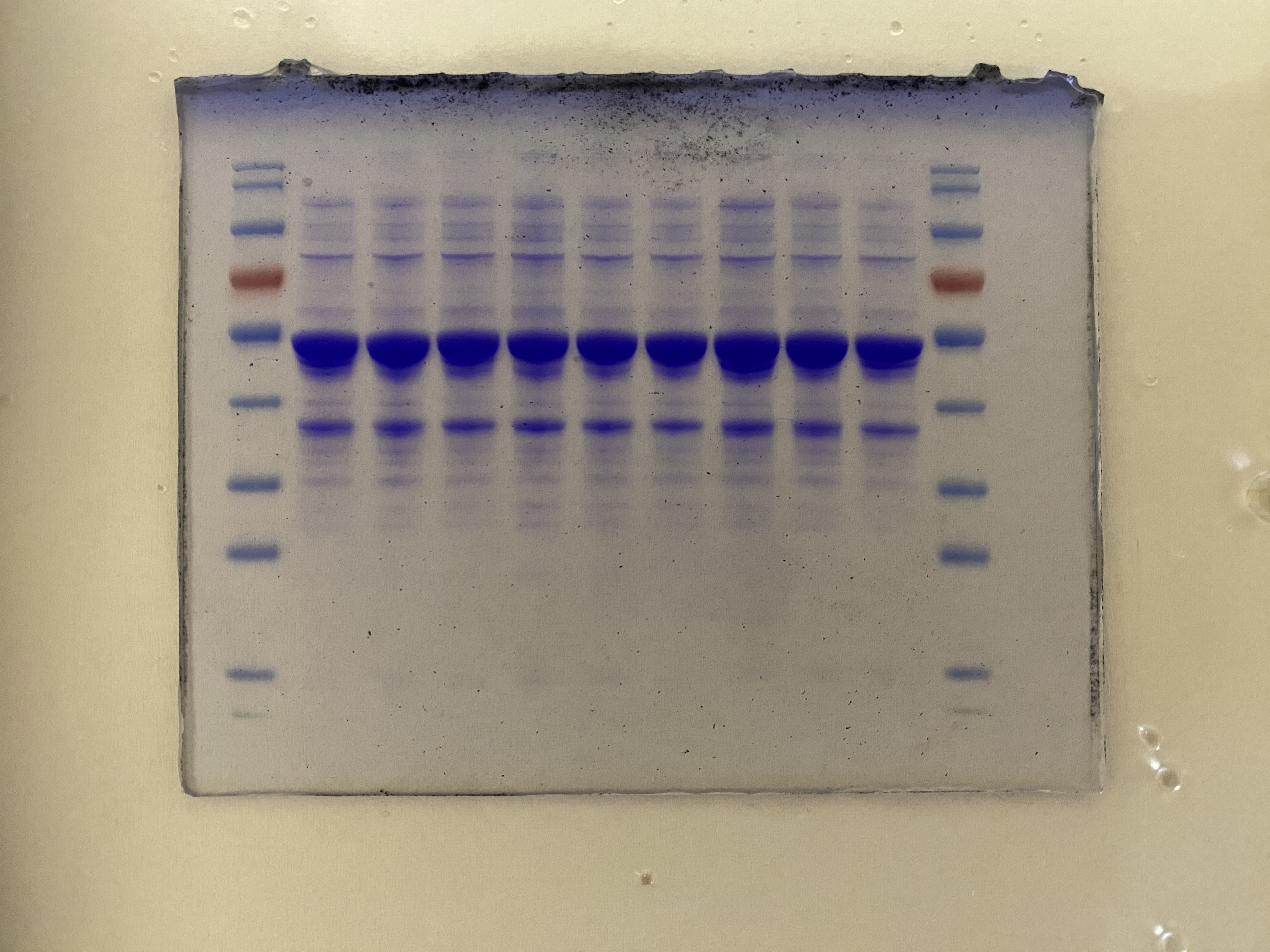


Figure S43: Uncropped SDS-PAGE for Figure S35 (right).

# Materials and methods

## Materials

### Chemicals

All chemicals and solvents, as not described otherwise, were purchased from commercial suppliers (Sigma Aldrich, Abcr, Alfa Aesar, Fisher Scientific, Isobionics, Acros Organics, Ambinter, BOC Science and aablocks) and used without additional purification.
The substrates geranylisopropanol**,** calmusol and geranylthioacetate for biocatalytic purposes were chemically synthesized and analyzed by 1H-NMR, 13C-NMR. Geranylisopropanol was synthesized by reduction of geranylacetone**.**  Calmusol was synthesized by the reduction of calmusal from BOC Sciences (762-26-5). Geranylthioacetate was synthesized in former work in three steps by reduction of geranylacetone to geranylisopropanol, with subsequent thioesterification to geranylthioacetate.

### *E.coli strains*

Three different *E. coli* strains were used for this work. *E.coli* XL1-blue was used for molecular biological work such as cloning and mutagenesis. *E.coli* BL21 (DE3) or *E.coli* ITB94 were used for heterologous gene expression. Strain ITB94 is a derivative of the commercially available TG1 strain, with L-rhamnose isomerase and two non-specific ADHs (ΔyahK and ΔyjgB) knocked out.

### Molecular biological kits

The molecular biological kits for DNA-purification (Zymoclean DNA Clean & Concentrator Kit), Agarose gel-extraction (Zymoclean Gel DNA Recovery Kit) and plasmid isolation (Zyppy™Plasmid Miniprep Kit) were purchased from ZymoResearch (Irvine, US).

### Buffer and media

The lists of all buffer and media ingredients is provided in Table S1and Table S2.

### Primer

The list of all primers is provided in Table S3, Table S4 and Table S5.

## General analytics

### Nuclear Magnetic Resonance

^1^H- und ^13^C-NMR spectra were recorded on a Bruker Avance 500 Spectrometer at 500,15 MHz for ^1^H and 125 MHz for ^13^C. The chemical shifts δ are referred to tetramethylsilane (TMS) in ppm set to 0. All substances were dissolved in CDCl3 and recorded at room temperature.

### Gas chromatography

GC analyses were performed using a Shimadzu GC-2010 Plus equipped with a flame ionization detector (FID) and a Zebron ZB-5 capillary column (Phenomenex, 30 m x 250 µm x 0.25 µm) or a DB-WAX column (Agilent Technologies, 30 m x 250 µm x 0.25 µm) with hydrogen as carrier gas with constant velocity (linear velocity: 33 cm/s). Injections of 1 µL were performed in split mode with 10:1.
GC/MS analyses were performed using an Agilent 7890A equipped with an Agilent 5975C detector and a DB-WAX capillary column (Agilent Technologies, 30 m x 250 µm x 0.25 µm) with helium as carrier gas with constant velocity (linear velocity 33.1 cm/s). Injections of 1 µL were performed in split mode with 10:1.
Temperature programs used for each substrate and GC are listed in Table S6 and Table S7.

### HPLC/MS

HPLC/MS was performed using an Agilent technologies 1260 Infinity. This system is equipped with a 1260 HiP Degaser, binary pump SL (G1312B), 1260 HiP ALS, 1260 TCC, 1260 DAD VL and a 6130 quadrupole LC/MS. The molecules were separated trough a Prontosil 120-3-C30 (Bischoff Chromatography, 100 cm x 4.6 mm, 3 µm). A premixture of MeOH:MTBE (A:B) + 0.1 % formic acid was used as mobile phase with a flow of 1.2 mL/min. The column was heated to 30 °C. DAD Signals were set to 445, 470 and 502. Masses were analyzed in positive scan mode in a range of 400 to 600 m/z and in sim mode at 555 and 573. The injection volume was set to 10 µL. Solvent gradients are listed in Table S8.

### Preparative HPLC

Preparative HPLC was performed using an Agilent technologies 1260 Infinity. This system is equipped with a 1200 Series Degaser, 1260 Quat Pump, 1260 ALS, 1260 TCC, 1260 DAD HS and a 1260 FC-AS. The molecules were separated trough a C18-column (Discovery®C18, 10 cm x 21.2 mm, 5 µm). ddH_2_0 (A) and acetonitrile (B) were used as mobile phase with a flow of 4 mL/min. The column was not heated. DAD Signals were set to 294, 210, 260 nm with a reference wavelength of 360 nm and also the spectrum from 190 to 600 nm with 2 nm steps was recorded. The injection volume of geranylacetone was 100 µL and for pseudoionone 150 µL per run. The fractions were time-based collected. Solvent gradients are listed in Table S9.

## Enzymatic preparative scale reactions

### Preparative biotransformation of hydroxyfarnesylacetone 3

The preparative reaction with **2** was carried out with the procedure described in section 2.5.13 Preparative scale reactions. 701 mg of **3** was obtained as yellow oil with an isolated yield of 40.0%.

**^1^H-NMR (CDCl_3_, 500 MHz)** δ= 5.08 (q, ^3^J_H,H_= 6.5Hz, 2H), 2.46 (t, ^3^J_H,H_= 7.3Hz, 2H), 2.27 (q, ^3^J_H,H_= 7.3Hz, 2H), 2.13 (s, 3H), 2.06 (m, 2H), 1.98 (m, 4H), 1.61 (s, 3H), 1.59 (s, 3H), 1.26 (t, ^3^J_H,H_= 7.1Hz, 1H), 1.21 (s, 6H) ppm.
**^13^C-NMR (CDCl_3_, 125 MHz)** δ= 208.95, 136.33, 136.98, 124.25, 122.59, 70.98, 43.78, 43.49, 40.01, 39.63, 29.92, 29.26 (2C), 26.44, 22.65, 22.49, 15.99, 15.89 ppm.

### Preparative biotransformation of hydroxygeranylacetone 8

The preparative reaction with **6** was carried out with the procedure described in section 2.5.13 Preparative scale reactions. 110 mg of **8** was obtained as yellow oil with an isolated yield of 10.4%.

**^1^H-NMR (CDCl_3_, 500 MHz)** δ= 5.08 (m, 1H), 2.46 (t, ^3^J_H,H_= 7.5Hz, 2H), 2.27 (m, 2H), 2.14 (s, 3H), 1.97 (t, ^3^J_H,H_= 6.8Hz, 2H), 1.61 (s, 3H), 1.42 (m, 4H), 1.21 (s, 6H) ppm.
**^13^C-NMR (CDCl_3_, 125 MHz)** δ= 209.04, 136.31, 122.71, 71.04, 43.78, 43.37, 39.91, 29.95, 29.23 (2C), 22.52, 22.44, 15.86 ppm.
^1^H-NMR and ^13^C-NMR are in accordance with literature.^[17]^

### Preparative biotransformation of hydroxypseudoionone 9

The preparative reaction with **7** was carried out with the procedure described in section 2.5.13 Preparative scale reactions. 123 mg of **9** was obtained as yellow oil with an isolated yield of 11.7%.

**^1^H-NMR (CDCl_3_, 500 MHz)** δ= 7.43 (m, 1H), 6.08 (d, ^3^J_H,H_= 15.3Hz, 1H), 6.02 (d, ^3^J_H,H_= 11.4Hz, 1H), 2.28 (s, 3H), 2.17 (t, ^3^J_H,H_= 7.61Hz, 2H), 1.91 (s, 3H), 1.56 (m, 2H), 1.44 (m, 2H) 1.22 (s, 6H) ppm.
**^13^C-NMR (CDCl_3_, 125 MHz)** δ= 198.92, 151.11, 139.58, 128.51, 123.84, 70.87, 43.28, 40.72, 29.32 (2C), 27.52, 22.35, 17.39 ppm.

### Preparative biotransformation of hydroxyhomofarnesol 13

The preparative reaction with **11** was carried out with the procedure described in section 2.5.13 Preparative scale reactions. 1117 mg of **13** was obtained as yellow oil with an isolated yield of 70.3%.

**^1^H-NMR (CDCl_3_, 500 MHz)** δ= 5.12 (dt, ^3^J_H,H_=32.4, 7.3Hz, 2H), 3.62 (t, ^3^J_H,H_= 6.4Hz, 2H), 2.29 (q, ^3^J_H,H_= 6.7Hz, 2H), 2.21 (m, 2H), 2.05 (m, 2H), 1.97 (t, ^3^J_H,H_= 7.1Hz, 2H), 1.64 (s, 3H), 1.59 (s, 3H), 1.43 (m, 4H), 1.21 (s, 6H) ppm.
**^13^C-NMR (CDCl_3_, 125 MHz)** δ= 138.64, 135.18, 124.16, 120.12, 71.00, 62.35, 43.39, 39.94, 39.75, 31.43, 28.21 (2C), 26.24, 22.61, 16.15, 15.91 ppm.

### Preparative biotransformation of hydroxyfarnesol 5

The preparative reaction with **farnesol** was carried out with the procedure described in section 2.5.13 Preparative scale reactions. 93 mg of **5** was obtained as yellow oil with an isolated yield of 38.8%.

**^1^H-NMR (CDCl_3_, 500 MHz)** δ= 5.40 (t, ^3^J_H,H_=6.7Hz, 1H), 5.09 (t, ^3^J_H,H_=6.7Hz, 1H), 4.16 (d, ^3^J_H,H_=6.8Hz, 2H), 2.13 (m, 2H), 2.06 (m, 2H), 1.98 (m, 2H), 1.67 (s, 3H), 1.59 (s, 3H), 1.42 (m, 3H), 1.21 (s, 6H) ppm.
**^13^C-NMR (CDCl_3_, 125 MHz)** δ= 139.15, 135.07, 124.21, 123.74, 71.19, 59.40, 43.18, 39.83, 29.12 (2C), 25.95, 22.48, 16.16, 15.85 ppm.
^1^H-NMR and ^13^C-NMR are in accordance with literature.^[18]^

### Preparative biotransformation of hydroxygeranyllinalool 16

The preparative reaction with **geranyllinalool** was carried out with the procedure described in section 2.5.13 Preparative scale reactions. 99 mg of **16** was obtained as yellow oil with an isolated yield of 32.1%.

**^1^H-NMR (CDCl_3_, 500 MHz)** δ= 5.40 (dd, ^3^J_H,H_=17.3, 10.7Hz, 1H), 5.21 (dd, ^3^J_H,H_=17.4, 1.2Hz, 1H), 5.10 (m, 3H), 2.03 (m, 8H), 1.59 (m, 8H), 1.43 (m, 4H), 1.28 (s, 3H), 1.21 (s, 6H) ppm.
**^13^C-NMR (CDCl_3_, 125 MHz)** δ= 145.06, 135.47, 134.97, 124.32, 124.28, 111.69, 73.52, 71.05, 43.47, 42.10, 39.99, 39.65, 29.25 (2C), 27.85, 26.37, 22.76, 22.65, 16.00, 15.89 ppm.

### Preparative biotransformation of hydroxy-α-farnesene 23

The preparative reaction with **α-farnesene** was carried out with the procedure described in section 2.5.13 Preparative scale reactions. 52 mg of **23** was obtained as yellow oil with an isolated yield of 23.3%.

**^1^H-NMR (CDCl_3_, 500 MHz)** δ= 6.37 (dd, ^3^J_H,H_=17.4, 10.8Hz, 1H), 5.46 (t, ^3^J_H,H_=7.4Hz, 1H), 5.13 (m, 2H), 5.07 (t, ^3^J_H,H_= 6.0Hz, 1H), 4.93 (d, ^3^J_H,H_= 10.7Hz, 1H), 2.84 (t, ^3^J_H,H_= 7.4Hz, 2H), 1.94 (m, 4H), 1.76 (s, 3H), 1.34 (m, 4H), 1.21 (s, 6H) ppm.
**^13^C-NMR (CDCl_3_, 125 MHz)** δ= 141.51, 135.72, 133.76, 131.78, 122.22, 110.59, 71.03, 43.49, 39.97, 29.25(2C), 27.20, 22.61, 15.99, 11.68 ppm.

## Chemical synthesis

### Synthesis of geranylisopropanol

For the synthesis of geranylisopropanol the substrate geranylacetone was reduced. For the reaction geranylaceton (0.50 mL, 2.34 mmol, 1.00 eq.) was dissolved in 10 mL of ethanol. Sodium borohydride (0.088 g, 2.34 mmol, 1.00 eq.) was then added and the reaction mixture was stirred for 1 h at room temperature. After one hour the reaction was quenched by adding 2 mL of 0.5 N HCl and stirred again for 30 min at room temperature. Afterwards 50 mL of distilled water was added and the aqueous phase was extracted three times with dichlormethane. The organic phases were combined and dried over CaCl_2_ and geranylisoporpanol was obtained as a clear oil (0.44 g, 2.04 mmol, 87 %).

**^1^H-NMR (CDCl3, 500 MHz)** δ= 5.15 (t, ^3^J_H,H_ = 6.8 Hz, 1H), 5.08 (t, ^3^J_H,H_ = 6.7 Hz, 1H), 3.81 (sept, ^3^J_H,H_ = 17.43 Hz, 1H), 2.08 (m, 4H), 1.99 (t, ^3^J_H,H_ = 7.3 Hz, 2H), 1.68 (s, 3H), 1.62 (s, 3H), 1.6 (s, 3H), 1.50 (quart, ^3^J_H,H_ = 7.7 Hz, 2H) 1.19 (d, ^3^J_H,H_ = 2.9 Hz, 3H) ppm.  **^13^C-NMR (CDCl3, 125 MHz)** δ= 135.37, 131.42, 124.37, 124.29, 71.09, 43.63, 39.72, 29.24, 26.69, 25.69, 23.04, 17.69, 15.97 ppm.
^1^H-NMR and ^13^C-NMR are in accordance with literature.^[19]^

### Synthesis of Calmusol

The reaction for the synthesis of calmusol was conducted in the same way as described in **(1) Synthesis of geranylisopropanol**, starting with the substrate calmusal. The product calmusol was obtained as a clear oil (0.24 g, 1.04 mmol, 43%).

**^1^H-NMR (CDCl3, 500 MHz)** δ= 5.13 (m,2H), 3.65 (t, ^3^J_H,H_ = 6.6 Hz, 2H), 2.05 (m, 4H), 1.70 (m, 2H), 1.69 (s, 3H), 1.64 (m,1H), 1,61(s, 3H), 1.58 (m, 2H) 1.25 (m, 2H) ppm.

**^13^C-NMR (CDCl3, 125 MHz)** δ= 135.95, 131.68, 124.58, 124.23, 62.81, 32.98, 31.92, 26.56, 25.72, 24.16, 23.40, 17.64 ppm.
^1^H-NMR and ^13^C-NMR are in accordance with literature.^[20]^

### Synthesis of geranylthioacetate 38

For the synthesis of geranylthioacetate the substrate geranylisopropanol was thio-acetylated. At the beginning to a solution of PPh_3_ (0,62 g, 2.3 mmol, 2.00 eq.) in THF (30 mL), DIAD (0,48 g, 2.3 mmol, 2.00 eq.) was added at -20 °C. The solution was stirred and warmed to 0 °C for 1 h. Then a 5m mL mixture of thioacetic acid (0,176 g, 2.3 mmol, 2.00 eq.) and geranylisopropanol (0.225 g, 1.15 mmol, 1.00 eq.) in THF was added dropwise for 20 min at -20 °C. Afterwards the reaction was warmed to 0 °C for 0.5h and left at 0 °C for 1 h and stirred overnight at room temperature. The mixture was then poured into 10 mL ddH_2_0 and extracted three times with diethylether. The organic phases were combined and purified via column chromatography with hexan/ethyl acetate (95:5). Geranylthio acetet was obtained as a yellow oil (95.41 mg, 0,375 mmol, 32.6 %).

**^1^H-NMR (CDCl_3_, 500 MHz)** δ= 5.09 (m, 2H), 3.54 (m, 1H), 5.13 (m, 2H), 2.30 (s, 3H), 2.03 (m, 6H), 1.68 (s, 3H), 1.58 (m, 8H), 1.30 (d, ^3^J_H,H_= 6.9Hz, 3H)ppm.
**^13^C-NMR (CDCl_3_, 125.77 MHz)** δ= 135.90, 131.38, 124.27, 123.24, 39.69, 39.32, 36.35, 30.80, 36.65, 25.70, 25.50, 23.38, 21.41, 17.70, 15.99 ppm.
^1^H-NMR and ^13^C-NMR are in accordance with literature.^[21]^

## General methods

### Cloning of *Rg*CrtC gens and Funclib variants

Sequences for Carotenoid-1,2-hydratase from *Rubrivivax gelantinosus* strain IL144/S1 (*Rg*CrtC_IL144 and *Rg*CrtC_S1; 4.1) were codon optimized for *E.coli*, ordered at TWIST Bioscience (California, USA) and cloned by Gibson Assembly ^[22]^ into pDHE1650 or pET22b(+) plasmid (Figure S36-Figure S38). The plasmid pDHE1650_*Rg*CrtC_IL144 was already cloned in a previous work at our institute.^[23]^ The plasmid constructs were then transformed via Heat shock into *E.coli* XL-1 blue and subsequently isolated and sequenced. The correct plasmid-construct was transformed for enzyme expression into *E.coli* ITB94 or *E.coli* BL21 (DE3) by heat shock.

### Expression-study of *Rg*CrtCs

Expression-study took place in lysogeny-broth (LB) or terrific-broth (TB). For both setups one single colony of the different plasmid/strain was picked from LB-agar plate (ampicillin, c_end_=150 µg/mL) to inoculate a 5 mL overnight LB culture (ampicillin, c_end_=150 µg/mL). After 16 h of incubation at 37 °C and 180 rpm, 1% (v/v) overnight culture was used to inoculate 50 mL of the corresponding media in a 250 mL shaking flask. Expression in TB-media was conducted after a protocol established recently in our institute.^[23]^

LB-media (ampicillin, c_end_=150 µg/mL) expression was incubated at 37 °C and 180 rpm shaking until OD_600_ of 0.6-0.8 was reached. The Expression was started by induction with 0.1 mM IPTG (pET22b(+)/BL21) or 0.5 g/L L-rhamnose (pDHE1650/ ITB94) for 20 h, 25 °C and 180 rpm shaking. The cells were harvested at 4000 x g, 4 °C for 20 min and freshly used for biotransformations.

Expression in TB-media (with ampicillin, c_end_=150 µg/mL) took place in autoinduction-manner by directly adding 0.1 mM IPTG (pET22b(+)/BL21) or 0.5 g/L L-rhamnose (pDHE1650/ ITB94) before inoculating with the overnight culture. The cultures were incubated for 24 h at 30 °C and 180 rpm shaking. The cells were harvested at 4000 x g, 4 °C for 20 min and freshly used for biotransformations.

After the expression study, further experiments were exclusively conducted with pDHE::wt*Rg*CrtC_IL144/ITB94.

### Expression for WC-biotransformations and purification

Overnight cultures in 5 mL LB-media (ampicillin, c_end_=150 µg/mL) were inoculated with one single colony from LB-agar plate or with a glycerol stock stored at -80 °C. After 16 h of incubation at 37 °C and 180 rpm, the main cultures were inoculated with 1 % (*v/v*)of the overnight culture.
For WT-biotransformations and purification the expression took place in 500 mL TB-autoinduction media in a 2L shaking flask for 24h at 30 °C and 180 rpm shaking. The cells were harvested at 4000 x g, 4 °C for 20 min and freshly or lyophilized used in biotransformations.
Expression for single-point mutation variants was conducted in 50 mL TB-autoinduction media in a 250 mL shaking flask for 24h at 30 °C and 180 rpm shaking. The cells were harvested at 4000 x g, 4 °C for 20 min and freshly used in biotransformations.

### Expression for 96-deep well plate (DWP) screening (homology-based mutagenesis)

Overnight culture in 96 DWPs with 1 mL/well LB-media were inoculated with a glycerol stocks stored at -80 °C and incubated for 16 h at 37 °C and 800 rpm in the plate shaker. The 96 DWP main culture with TB-autoinduction media was inoculated with 1 % overnight culture and incubated for 24 h at 30 °C and 800 rpm in the plate shaker. Each 96 DWP contained 3x wt_*Rg*CrtC_IL144, 3x *Rg*CrtC_IL144_D268A(KO), 3x pDHE1650_empty, 3x wells just with media and triplicates of each homology-based mutagenesis variant. After 24 h the plates were harvested at 4000 x g, 4 °C for 20 min and freshly used for biotransformations.

### Semi-purification of *Rg*CrtC

Freshly harvested cells were homogenized in 5 mL/g_cww_ cold KPi buffer (50mM KPi pH=7.0). Cells were disrupted at 4 °C with 800 bar by an Emulsiflex-C5 (Avestin Inc.) and subsequently centrifuged at 45.000 x g, 4 °C for 1 h. The pellet was washed by resuspending in cold KPi buffer (50mM KPi pH=7.0) and subsequent centrifuged at 45.000 x g, 4 °C for 1 h. The washing procedure was done three times. After the last washing step, the pellet was resuspended in solubilization buffer (50mM KPi pH=7.0 + 1% w/v Chaps) for 24 h at 4 °C and 40 rpm inverting. After solubilization the suspension was centrifuged at 45.000 x g, 4 °C for 1 h and the supernatant was directly used in biotransformations for cofactor dependency investigations.

### Purification of *Rg*CrtC

Freshly harvested cells were homogenized in 5 mL/g_cww_ cold lysis buffer (20mM KPi pH=7.4, 20mM Imidazol, 500 mM NaCl). Cells were disrupted at 4 °C with 800 bar by an Emulsiflex-C5 (Avestin Inc.) and subsequently centrifuged at 45.000 x g, 4 °C for 1 h. The pellet was resuspended in 5mL/g_cww_ cold solubilization buffer (20mM KPi pH=7.4, 20mM Imidazol, 500 mM NaCl, 1% w/v Chaps) for 24 h at 4 °C and 40 rpm inverting. The solubilized solution was again centrifuged at 45.000 x g, 4 °C for 1 h and the supernatant was filtered through 0.2 µm. The enzyme solution was then purified at 4 °C with an Äkta™ pure (GE-healthcare) equipped with a HisTrap™ HP 5 ml column (cytiva) and a UV/Vis-detector to monitor protein absorbance at 280 nm during the run. The flow rate was set to 5 mL/min and the column was equilibrated with 3 cvs of Purification buffer A. The solubilized supernatant was loaded and then washed with 10 cvs of Purification buffer A. After washing the protein was eluted with 100 % Purification buffer B for 5 cvs. The Protein containing fractions were pooled and desalted at 4 °C with an Äkta™ pure (GE-healthcare) equipped with a HiPrep™ 26/10 53.1 mL desalting column (cytiva). The flow rate was set to 9 mL/min and the column was equilibrated with 2 cvs of Desalting buffer. The pooled fractions were loaded onto the column and eluted with 100 % Desalting buffer for 20 cvs. The protein containing fractions were pooled and concentrated with VIVASPIN 20 (Membrane 10.000 MWCO PES, Sartorius) at 4.000 x g and 4°C. After concentration the protein concentration was measured via Pierce™ Rapid Gold BCA Protein Assay Kit (Thermo Scientific). The purified enzyme was aliquoted, frozen and stored at -80°C.

### Mutagenesis of *Rg*CrtC IL144

Site-directed mutagenesis of wt*Rg*CrtC_IL144 variants was conducted by using QuikChange-PCR™. After Dpn1-digest (1 µL Dpn1 to 25 µL PCR-product) for 4 h at 37 °C Afterwards the plasmid was transformed into competent *E.coli* XL-1 blue and subsequently isolated and sequenced. The correct plasmid-construct was transformed for enzyme expression into *E.coli* ITB94.

### Biotransformations for lycopene wild type reaction

Biotransformations for lycopene conversion were conducted by using lysed cells. Freshly harvested cells were homogenized in 3 mL/g_cww_ cold 50 mM KPi-buffer pH=8.0. Cells were disrupted at 4 °C with 800 bar by an Emulsiflex-C5 (Avestin Inc.) and directly used for biotransformations. Biotransformations were performed in a 2 mL glass vial (Carl Roth) by incubating 495µL of the lysed cell suspension with 5µL substrate stock (250 mM lycopene in acetone; c_end_=2.5 mM) for 20 h at 30°C and 300 rpm. These biotransformations took place in technical triplicates with empty vector controls and buffer controls. Reaction were stopped by extracting with 1mL MTBE by vortexing, shaking and inverting After extraction the samples were centrifuged at 4000 x g for 10 min at room temperature for phase separation. From the organic phase, 300 µL was transferred to GC vials with inlet and the samples were analyzed by HPLC/MS.

### Biotransformations for expression study, reaction parameter optimization and active-site residues

For investigating the different expression setups (plasmid/strains) fresh cells were resuspended in 50 mM KPi-buffer pH=7.0 to obtain a cell suspension with a concentration of 100 mg_cww_/mL. Biotransformations were performed in a 2 mL glass vial (Carl Roth) by incubating 495µL of the cell suspension with 5µL substrate stock (100mM farnesylacetone in DMSO; c_end_=1 mM) for 24 h at 30°C and 300 rpm. These biotransformations took place in technical triplicates with empty vector controls and buffer controls.
Reaction were stopped by extracting with 1mL cyclohexane/ethyl acetate (1:1) + 0.5 mM hexadecane internal standard by vortexing, shaking and inverting After extraction the samples were centrifuged at 4000 x g for 10 min at room temperature for phase separation. From the organic phase, 300 µL was transferred to GC vials with inlet and the samples were analyzed by GC/FID. The concentration of the product was determined using the internal standard and the respective effective carbon number.^[24,25]^
Investigation of the reaction parameters took place in different setups. For buffer experiments fresh cells were resuspended in 50 mM KPi-, NaPi-, Citrat- and Tris-buffer in all given pH values (Table S1) to obtain a cell suspension with 100mg_cww_/mL. Biotransformations and extraction were performed as described at the beginning of this chapter.
Temperature parameters were investigated by resuspending fresh cells in 50 mM KPi pH=7.0 ending up with a cell concentration of 100 mgcww/mL. Biotransformations and extraction were performed as described at the beginning of this chapter. Only the temperature parameter during biotransformation was adjusted to 25, 30, 37, 40, 50 and 60 °C.
For (2-Hydroxypropyl)-β-cyclodextrin (2HPCD) concentration experiments cells were resuspended to a cell concentration of 100 mgcww/mL in 50 mM KPi-buffer pH=7.0 containing 0, 0.25, 0.5, 1, 2, 5 or 10 mM of 2HPCD. Biotransformations and extraction were performed as described at the beginning of this chapter.
For investigating the difference between fresh and lyophilized cells, lyophilized cells were resuspended in 50 mM KPi-buffer pH=7.0 to a cell concentration of 35 mg_cdw_/mL, which equals 100 mgc_ww_/mL considering the shrinking-factor. Biotransformations and extraction were performed as described at the beginning of this chapter.
Investigation of the site-directed mutagenesis variants was done by resuspending fresh cells in 50 mM KPi-buffer pH=7.0 to a cell concentration of 100 mgcww/mL. Biotransformations and extraction were performed as described at the beginning of this chapter.

### Biotransformations with semi purified *Rg*CrtC_IL144

The influence of the addition of cofactors was investigated by using solubilized enzyme solution. To the solubilized enzyme (from 100 mgcww/mL) in 50 mM KPi pH = 7.0, individually 2 mM FAD (cend = 0.1 mM), 100 mM NADH (cend = 1 mM), 100 mM NADPH (cend = 1 mM) or a combination of FAD+NADH or FAD+NADPH were added. Biotransformations were incubated in 2 mL glass vials (Carl Roth®, Karlsruhe, DE) with 495 µL of the cell suspension and 5 µL substrate stock (100 mM farnesyl acetone in DMSO; cend = 1 mM) for 24 h at 30 °C and 300 rpm in a shaking incubator. These biotransformations were prepared in triplicates with additional buffer controls. Reaction were stopped by extracting with 1mL cyclohexane/ethyl acetate (1:1) + 0.5 mM hexadecane internal standard by vortexing, shaking and inverting After extraction the samples were centrifuged at 4000 x g for 10 min at room temperature for phase separation. From the organic phase, 300 µL was transferred to GC vials with inlet and the samples were analyzed by GC/FID. The concentration of the product was determined using the internal standard and the respective effective carbon number.

### Biotransformations with purified *Rg*CrtC_IL144

Frozen purified enzyme was unfrozen and carefully mixed with NaPi-Buffer 50 mM pH 7.0 to get an end concentration of 135 µM enzyme in 495 µL. Biotransformations were performed in a 2 mL glass vial (Carl Roth) by incubating 495µL of enzyme solution with 5µL substrate stock (100mM farnesylacetone in DMSO; c_end_=1 mM) for 24 h at 30°C and 300 rpm. These biotransformations took place in technical triplicates with buffer controls.
Reaction were stopped by extracting with 1mL cyclohexane/ethyl acetate (1:1) by vortexing, shaking and inverting After extraction the samples were centrifuged at 4000 x g for 10 min at room temperature for phase separation. From the organic phase, 300 µL was transferred to GC vials with inlet and the samples were analyzed by GC/FID. The concentration of the product was determined using the internal standard and the respective effective carbon number.

### Biotransformations for substrate-screening

For investigating the substrate scope of wt_*Rg*CrtC_IL144 fresh cells were resuspended in WC-buffer to a cell concentration of 100 mg_cww_/mL. Biotransformations were performed in 2 mL glass vials (Carl Roth) by incubating 495µL of the cell suspension with 5µL of the respective substrate stock (100mM substrate in DMSO; cend=1 mM) for 24 h at 30°C and 300 rpm. These biotransformations were performed in triplicate with empty vector controls and buffer controls.
Reaction were stopped by extracting with 1mL cyclohexane/ethyl acetate (1:1) + 0.5 mM hexadecane or dodecane depending on the substrate as internal standard by vortexing, shaking and inverting. After extraction the samples were centrifuged at 4000 x g for 10 min at room temperature for phase separation. From the organic phase, 300 µL was transferred to GC vials with inlet and the samples were analyzed by GC/FID and GC/MS. The concentration of the product was determined using the internal standard and the respective effective carbon number.
For homofarnesylic acid and the corresponding hydrated product a derivatization step before GC measurement was necessary. Therefore, the extraction took place in the same manner as for the other substrates. After phase separation 300 µL organic phase was transferred into a 1.5 mL reaction tube (Eppendorf) and the solvent was evaporated for 30 min in a Concentraator 5301 (Eppendorf). After evaporating the solvent, 300 µL of cyclohexane/ethyl acetate/BSTFA (1:1:2) was used to solubilize the residual product in the reaction tube by vortexing. The solubilized mixture was transferred to GC vials with inlet and incubated for 1 h at 70 °C before analyzing via GC/FID.

### Cascade reaction of aldol condensation and subsequent hydration

One pot cascade reaction of aldol condensation coupled with hydration reaction was conducted with lyophilized cells solubilized in KPi-buffer pH=8.0 to obtain a cells solution with a concentration of 10 mg_cdw_/mL, which equals 35 mg_cww_/mL considering the shrinking-factor. Biotransformations were performed in a 2 mL glass vial (Carl Roth) by incubating 495µL of the cell suspension with 5µL substrate stock (500mM citral in acetone; c_end_=5 mM) for 24 h at 30°C and 300 rpm. As controls all reactions were done in Citrate-buffer pH=6.0 and with pseudoionone as a substrate as well as DMSO as cosolvent. These biotransformations took place in technical triplicates with empty vector controls and buffer controls. Reaction were stopped by extracting with 1mL cyclohexane/ethyl acetate (1:1) by vortexing, shaking and inverting After extraction the samples were centrifuged at 4000 x g for 10 min at room temperature for phase separation. From the organic phase, 300 µL was transferred to GC vials with inlet and the samples were analyzed by GC/FID and GC/MS.

### Biotransformations for 96 DWPs (Homology-based mutagenesis)

Investigation of the homology-based variants with substrate **2,6,7** and **11** was conducted by resuspending the fresh cell pellets in 495 µL WC-buffer. Afterwards the cell solution was transferred into a 96 DWP with glass inlets (Hirschmann) and 5µL of the respective substrate stock (100mM substrate in DMSO; cend=1 mM) was added and incubated for 5 h at 30 °C and 300 rpm.
For the investigation of the shorter substrates (≤ C_12_) fresh cell pellets were resuspended 495 µL WC-buffer. Afterwards the cell solution was transferred into a 96 DWP with glass inlets (Hirschmann) and 5µL of the respective substrate stock (100mM substrate in DMSO; cend=1 mM) was added and incubated for 24 h at 30 °C and 300 rpm.
Reactions were stopped by extracting with 500 µL cyclohexane/ethyl acetate (1:1) by shaking and inverting. After extraction the samples were centrifuged at 4000 x g for 10 min at room temperature for phase separation. From the organic phase, 200 µL was transferred to GC vials with inlet and the samples were analyzed by GC/FID or GC/MS.

### Preparative scale reactions

In order to determine the generated products, biotransformations were performed on a 100 mL (Substrates farnesol, Geranyllinalool and α-farnesene), 125 mL (Substrates farnesylacetone and homofarnesol) and 500 mL (substrate geranylacetone and pseudoionone) scale in 100 mL, 250 mL or 500 mL Schott flasks. For this purpose, a cell suspension was prepared with 40 mg_cww_/mL in WC-buffer. Subsequently 10 mM (farnesol, Geranyllinalool, α-farnesene, geranylacetone and pseudoionone) or 50 mM (farnesylacetone and homofarnesol) pure substrate was added to the cell suspension. The bottle was then sealed and shaken at 30 °C and 220 rpm for 5 d. The products **3**, **5**, **8**, **9**, **13**, **16** and **23** were then extracted from the aqueous phase by extracting three times with cyclohexane/ethyl acetate (1:1) (in total 1 L solvent).

The organic phase was concentrated *in* *vacuo* and the crude product was stored until further purification at 4 °C. Products of geranylacetone and pseudoionone were solved in ddH2O/acetonitrile (70:30) and purified via preparative HPLC (Table S9). Products **3**, **5**, **13**, **16** and **23** were solved in DCM and purified by column chromatography with silica gel 60M 0.04-0.063 mm (Table S10). The determination of the product structures was done by NMR and the yields are given in Figure S25.

### Cavity analysis and YASARA^®^ docking

The enzyme *Rg*CrtC IL144 (Uniprot: I0HUL0) was modeled using AlphaFold 2^[13]^. The best-scored relaxed model out of five was chosen for further investigations. The N-terminal part of the AlphaFold model exhibited low pLDDT values and contained an unfolded segment. To ensure accurate computational analyses, the first 101 N-terminal residues were removed.

The enzyme’s active site and substrate tunnel were investigated using the Catalophore^TM^ technology^[26]^, which represents the physico-chemical properties of the enzyme cavities by using a 3D-point cloud. We focused our analyses on the shape of the cavity. An elongated cavity extending from the protein surface to residue D268 was identified as substrate binding pocket due to its compatibility with the natural substrate’s shape. Residues adjacent to the cavity were selected for mutagenesis and conservation analysis.

Docking studies were performed using YASARA^[27]^, incorporating Autodock and VINA algorithms for the calculation of ligand-receptor interactions. Test runs with both algorithms showed that VINA^[28]^ with default settings gives promising results and was thus utilized further. As ligands all converted substrates along with the natural substrate lycopene and the receptor were prepared using YASARA. For the docking runs, residue D268 was changed to its protonated, acidic state to mimic the catalytically active form of the hydratase. The docking cell was adjusted in size based on the ligand, and multiple runs with different setting were conducted for all ligands. The cell was centered around residue D268 accept for lycopene, for which care was taken that the complete binding channel was covered. Each run calculated 25 docking poses, and those with a distance of less than 4 Å between the ligand’s double bond of the terminal prenyl unit and D268’s carboxyl group were further refined with the YASARA’s rescoring tool.

### Generating FuncLib

For generating the FuncLib library with the FuncLib algorithm^[29]^ (https://ablift.weizmann.ac.il/step/fl_terms/) 8 amino acid residues around the active- site/cavity were chosen (W110, I127, F129, V133, F134, N158, Y363 and M384) and D268 was excludes as it is the possible catalytical residue. The generated library was built with the alpha fold model of *Rg*CrtC_Il144. The following parameters were set: min ID 35, max targets 4000, coverage 75 and E value of 0.0001. Parameters for energy calculations and clustering were set as followed: minimal number of mutations per design = 3, maximal number of mutations per design = 5, minimal PSSM threshold = -1, ΔΔG = 6 and difference between clustered variants = 2.

### Multiple sequence alignment and weblogo

A blastp search was performed on the NCBI database of non-redundant protein sequences using the sequence of *Rg*CrtC IL144 as the query.^[30,31]^ After filtering the results to include only sequences with at least 20% sequence identity to the query enzyme and at least 75% query and subject coverage, 171 sequences remained. These sequences were aligned using the multiple sequence alignment function of clustalw2.^[32]^ For the residues close to the substrate binding site, the amino acid distribution among the homologous sequences has been extracted and analyzed.

Weblogo of conserved amino acid residues was generated by using WebLogo3.^[33]^

Table S1: List of buffers used in this work.

| **Buffer** | **Ingredients** |
| --- | --- |
| 10x phosphate buffer (for TB-Media) | 0.17 M KH_2_PO_4_, 0.72 M K_2_HPO_4_ |
| KPi-buffers (reaction parameter optimization) | 6.7 mM KH_2_PO_4_, 43.3 mM K_2_HPO_4_, pH=6.0 30.75 mM KH_2_PO_4_, 19.25 mM K_2_HPO_4_, pH=7.0 47 mM KH_2_PO_4_, 3 mM K_2_HPO_4_, pH=8.0 |
| NaPi-buffers (reaction parameter optimization) | 44 mM NaH_2_PO_4_, 6.0 mM Na_2_HPO_4_, pH=6.0 21.15 mM NaH_2_PO_4_, 28.85 mM Na_2_HPO_4_, pH=7.0 3.4 mM NaH_2_PO_4_, 46.6 mM Na_2_HPO_4_, pH=8.0 |
| Citrat-buffer (reaction parameter optimization) | 50 mM Citricacid, pH=5.0 (adjusted with NaOH) 50 mM Citricacid, pH=6.0 (adjusted with NaOH) |
| Tris-buffer (reaction parameter optimization) | 50 mM Tris, pH=8.0 (adjusted with HCl) 50 mM Tris, pH=9.0 (adjusted with HCl) |
| Lysis buffer | 4.5 mM NaH_2_PO_4_, 15.5 mM Na_2_HPO_4_, pH=7.4, 20mM Imidazol, 500 mM NaCl |
| Purification buffer A (wash buffer) | 4.5 mM NaH_2_PO_4_, 15.5 mM Na_2_HPO_4_, pH=7.4, 20 mM Imidazol, 500 mM NaCl, 1% w/v Chaps |
| Purification buffer B (elution buffer) | 4.5 mM NaH_2_PO_4_, 15.5 mM Na_2_HPO_4_, pH=7.4, 500 mM Imidazol, 500 mM NaCl, 1% w/v Chaps |
| Desalting buffer | 11.3 mM NaH_2_PO_4_, 38.7 mM Na_2_HPO_4_, pH=7.4, 300 mM NaCl, 1% w/v Chaps, 5% v/v Glycerol |
| WC-Buffer | 28.5 mM NaH_2_PO_4_, 21.5 mM Na_2_HPO_4_, pH=7.0,  1 eq.^a^ (2-Hydroxypropyl)-β-cyclodextrin |

^a^ to substrate concentration

Table S2: List of media used in this work.

| **Media** | **Ingredients** | **Steril additions to autoclaved media** |
| --- | --- | --- |
| Lysogeny broth (LB-media) | 10 g/L tryptone, 5 g/L yeast extract, 10 g/L NaCl | + ampicillin, c_end_=150 µg/mL |
| Terrific broth (TB-media) | 12 g/L tryptone, 24 g/L yeast extract, 5 g/L glycerol | + ampicillin, c_end_=150 µg/mL  + 10% 10x phosphate buffer |
| TB-autoinduction media | 12 g/L tryptone, 24 g/L yeast extract, 5 g/L glycerol | + ampicillin, c_end_=150 µg/mL  + 10% 10x phosphate buffer  + 0.5 g/L rhamnose |

Table S3: List of primer for Gibson assembly used in this work

| **Name** | **Sequence (5´🡪 3´) forward/reverse** |
| --- | --- |
| pDHE_*Rg*CrtC_IL144 Fragment | GTGCCGCGCGGCAGCCATATGCGTGCAGCA/ CGCCAAAACAGCCGGTACCCTTAGCTACGACGAGGCATACG |
| pDHE_*Rg*CrtC_IL144 Backbone | GTATGCCTCGTCGTAGCTAAGGGTACCGGCTG/ CGCTTTCTGCTGCACGCATATGGCTGCCGC |
| pDHE_*Rg*CrtC_S1 Fragment | GTGCCGCGCGGCAGCCATATGCGCGCAGCG/ CGCCAAAACAGCCGGTACC CTTAGGCACGACGTGGCATACG |
| pDHE_*Rg*CrtC_S1 Backbone | GTATGCCACGTCGTGCCTAAGGGTACCGGCTG/ CACTTTCCGCTGCGCGCATATGGCTGCCG |
| pET22_*Rg*CrtC_S1 Fragment | CTTTAAGAAGGAGATATACATATGCGCGCAGCGG/ GTGGTGGTGGTGCTCGAGGGCACGACG |
| pET22_RgCrtC_S1 Backbone | CGTATGCCACGTCGTGCCCTCGAGCACC/ CCACTTTCCGCTGCGCGCATATGTATATCTCCTTCTTAAAGTTAAACAAAATTATTTCTAGAGGG |

Table S4: List of Primer for single point mutations used in this work.

| **Name** | **Sequence (5´🡪 3´) forward/reverse** |
| --- | --- |
| pDHE_*Rg*CrtC_IL144_D268A | **GTCATGCCTATGTTGCTAGCAATGAAGGTG/ CACCTTCATTGCTAGCAACATAGGCATGAC** |
| pDHE_*Rg*CrtC_IL144_D268L | CATGCCTATGTTCTGAGCAATGAAGGTG/ CACCTTCATTGCTCAGAACATAGGCATG |
| pDHE_*Rg*CrtC_IL144_D268N | CATGCCTATGTTAACAGCAATGAAGGTG/ CACCTTCATTGCTGTTAACATAGGCATG |
| pDHE_*Rg*CrtC_IL144_W110A | CCGGGTGGTTATCTGGCGTGGTATGTTGATGCAG/ CTGCATCAACATACCACGCCAGATAACCACCCGG |
| pDHE_*Rg*CrtC_IL144_W110F | CCGGGTGGTTATCTGTTCTGGTATGTTGATGCAG/ CTGCATCAACATACCAGAACAGATAACCACCCGG |
| pDHE_*Rg*CrtC_IL144_W283A | GTCCGTTTCGTGAAGCGGATTGGAGCCGTGC/ GCACGGCTCCAATCCGCTTCACGAAACGGAC |
| pDHE_*Rg*CrtC_IL144_W283F | CGTCCGTTTCGTGAATTCGATTGGAGCCGTGC/ GCACGGCTCCAATCGAATTCACGAAACGGACG |
| pDHE_*Rg*CrtC_IL144_W285A | CGTGAATGGGATGCGAGCCGTGCAACCATG/ CATGGTTGCACGGCTCGCATCCCATTCACG |
| pDHE_*Rg*CrtC_IL144_W285F | CGTGAATGGGATTTCAGCCGTGCAACCATGG/ CCATGGTTGCACGGCTGAAATCCCATTCACG |

Table S5:List of primers for homology-based single point mutagenesis used in this work.

| **Name** | **Sequence (5´🡪 3´) forward/reverse** |
| --- | --- |
| pDHE_*Rg*CrtC_IL144_S132C | GCATTTGTTGGTTGCGTTTTCAGCCCG/ CGGGCTGAAAACGCAACCAACAAATGC |
| pDHE_*Rg*CrtC_IL144_S132M | GCATTTGTTGGTATGGTTTTCAGCCCG/ CGGGCTGAAAACCATACCAACAAATGC |
| pDHE_*Rg*CrtC_IL144_S132N | GCATTTGTTGGTAACGTTTTCAGCCCG/ CGGGCTGAAAACGTTACCAACAAATGC |
| pDHE_*Rg*CrtC_IL144_V133Y_F134C | CATTTGTTGGTAGCTATTGCAGCCCGTATTATG/ CATAATACGGGCTGCAATAGCTACCAACAAATG |
| pDHE_*Rg*CrtC_IL144_V133F_F134V | CATTTGTTGGTAGCTTTGTGAGCCCGTATTATG/ CATAATACGGGCTCACAAAGCTACCAACAAATG |
| pDHE_*Rg*CrtC_IL144_V133P_F134M | CATTTGTTGGTAGCCCGATGAGCCCGTATTATG/ CATAATACGGGCTCATCGGGCTACCAACAAATG |
| pDHE_*Rg*CrtC_IL144_V133Y_F134C_S132C | GCATTTGTTGGTTGCTATTGCAGCCCG/ CGGGCTGCAATAGCAACCAACAAATGC |
| pDHE_*Rg*CrtC_IL144_V133Y_F134C_S132M | GCATTTGTTGGTATGTATTGCAGCCCG/ CGGGCTGCAATACATACCAACAAATGC |
| pDHE_*Rg*CrtC_IL144_V133Y_F134C_S132N | GCATTTGTTGGTAACTATTGCAGCCCG/ CGGGCTGCAATAGTTACCAACAAATGC |
| pDHE_*Rg*CrtC_IL144_V133F_F134V_S132C | GCATTTGTTGGTTGCTTTGTGAGCCCG/ CGGGCTCACAAAGCAACCAACAAATGC |
| pDHE_*Rg*CrtC_IL144_V133F_F134V_S132M | GCATTTGTTGGTATGTTTGTGAGCCCG/ CGGGCTCACAAACATACCAACAAATGC |
| pDHE_*Rg*CrtC_IL144_V133F_F134V_S132N | GCATTTGTTGGTAACTTTGTGAGCCCG/ CGGGCTCACAAAGTTACCAACAAATGC |
| pDHE_*Rg*CrtC_IL144_V133P_F134M_S132C | GCATTTGTTGGTTGCCCGATGAGCCCG/ CGGGCTCATCGGGCAACCAACAAATGC |
| pDHE_*Rg*CrtC_IL144_V133P_F134M_S132M | GCATTTGTTGGTATGCCGATGAGCCCG/ CGGGCTCATCGGCATACCAACAAATGC |
| pDHE_*Rg*CrtC_IL144_V133P_F134M_S132N | GCATTTGTTGGTAACCCGATGAGCCCG/ CGGGCTCATCGGGTTACCAACAAATGC |
| pDHE_*Rg*CrtC_IL144_S132C | GCATTTGTTGGTTGCGTTTTCAGCCCG/ CGGGCTGAAAACGCAACCAACAAATGC |
| pDHE_*Rg*CrtC_IL144_S132M | GCATTTGTTGGTATGGTTTTCAGCCCG/ CGGGCTGAAAACCATACCAACAAATGC |
| pDHE_*Rg*CrtC_IL144_S132N | GCATTTGTTGGTAACGTTTTCAGCCCG/ CGGGCTGAAAACGTTACCAACAAATGC |

Table S6: GC/FID temperature programs used in this work.

| **Substrates** | **Internel Standard** | **Inlet Temp. [°C]** | **column** | **Split** | **Start Temp. [°C]** | **End Temp. [°C]** | **Rate [°C/min]** | **Hold [min]** |
| --- | --- | --- | --- | --- | --- | --- | --- | --- |
| farnesylacetone  bishomofarnesol  homofarnesol  farnesol  geranyllinalool  homofarnesylacid  homofarnesylacetate  geranylthioacetate | Hexadecan | 260 | ZB-5 | 10 | 200 | 300 | 10 | 1 |
| calmusol  geranylbutylether  α-/β-farnesene | Dodecan | 260 | ZB-5 | 10 | 140 | 300 | 10 | 1 |
| geranylaceton  pseudoionone  geranylisopropanol  geranylacetate  geranylethylether  santalene  bergamoten | Dodecan | 260 | ZB-5 | 10 | 120 | 300 | 10 | 1 |
| Cascade reaction | - | 260 | ZB-5 | 10 | 70 | 300 | 10 | 1 |
| calmusal | - | 260 | ZB-5 | 10 | 120 | 300 | 10 | 1 |
| geraniol  (+)-/(-)-isopulegol | - | 260 | ZB-5 | 10 | 110 | 300 | 10 | 1 |
| 6-methyl-5-hepten-2-one  citral  (*R*)-/(*S*)-citronellal | - | 260 | ZB-5 | 10 | 100 | 300 | 10 | 1 |
| linalool  myrcen  (+)-/(-)-β-citronellen | - | 260 | ZB-5 | 10 | 90 | 300 | 10 | 1 |
| (*R*)-/(*S*)-limonen  (+)-/(-)-α-pinene  terpinolene  sabinen | - | 240 | DB-Wax | 10 | 130 | 230 | 10 | 1 |

Table S7: GC/MS temperature programs used in this work.

| **Substrates** | **Internel Standard** | **Inlet Temp. [°C]** | **Column** | **Split** | **Start Temp. [°C]** | **End Temp. [°C]** | **Rate [°C/min]** | **Hold [min]** | **Scan-mode [m/z]** |
| --- | --- | --- | --- | --- | --- | --- | --- | --- | --- |
| farnesylacetone  bishomofarneso  homofarnesol  farnesol  geranyllinalool  homofarnesylacetate  geranylthioacetate | Hexadecan | 300 | DB-Wax | 10 | 50 | 230 | 10 | 10 | 50-350 |
| calmusol  geranylbutylether  α-/β-farnesene  geranylacetone  pseudoionone  geranylisopropanol  geranylacetate  geranylethylether  santalene  bergamoten | Dodecan | 300 | DB-Wax | 10 | 50 | 230 | 10 | 10 | 50-350 |
| Cascade-reaction | - | 300 | DB-Wax | 10 | 50 | 230 | 10 | 10 | 50-350 |

Table S8: HPLC program for lycopene **1** used in this work.

| **Substrates** | **Time [min]** | **Solvent mixture**  **A/B [%]** | **Flow [mL/min]** | **Max. Pressure  Limit [bar]** |
| --- | --- | --- | --- | --- |
| lycopene | 0.00  28.00 | 60.0 / 40.0  60.0 / 40.0 | 1.200 | 250.00 |

Table S9: Preparative HPLC program for geranylacetone and pseudoionone used in this work.

| **Substrates** | **Time [min]** | **Solvent mixture**  **A/B [%]** | **Flow [mL/min]** | **Max. Pressure  Limit [bar]** |
| --- | --- | --- | --- | --- |
| geranylacetone  pseudoionone | 0.00  3.00  23.00  30.00  30.01  40.00 | 70.0 / 30.0  70.0 / 30.0  10.0 / 90.0  10.0 / 90.0  70.0 / 30.0  70.0 / 30.0 | 4.000 | 250.00 |

Table S10: Solvent mixture for preparative column chromatography used in this work.


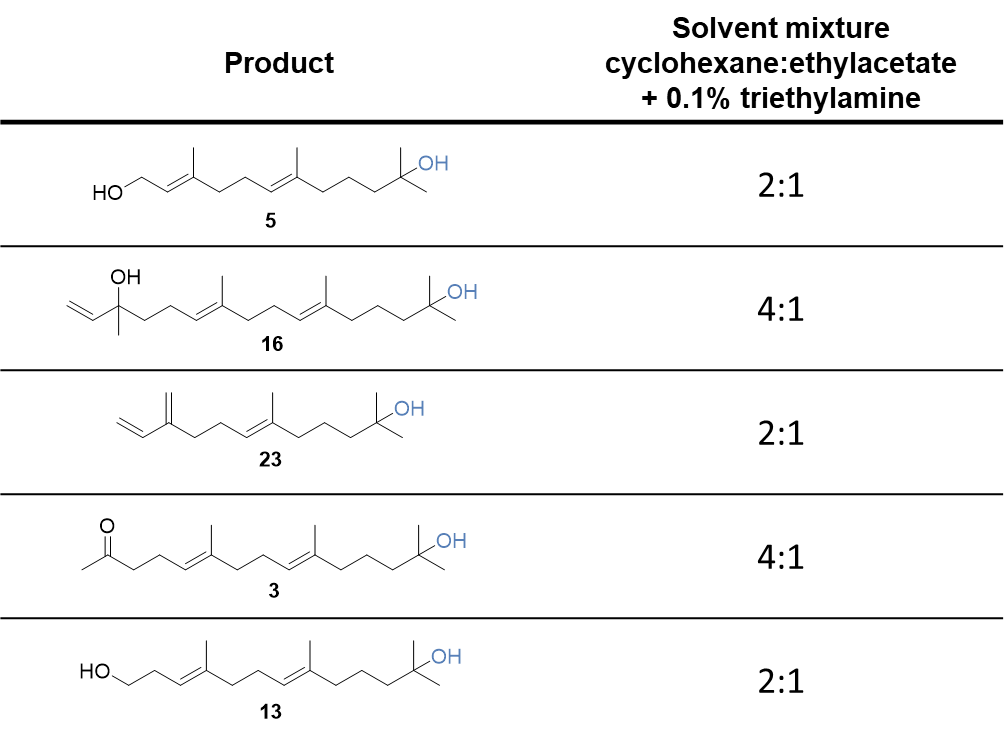


# NMR spectra

**Hydroxyfarnesylaceton 3:**

**^1^H-NMR**


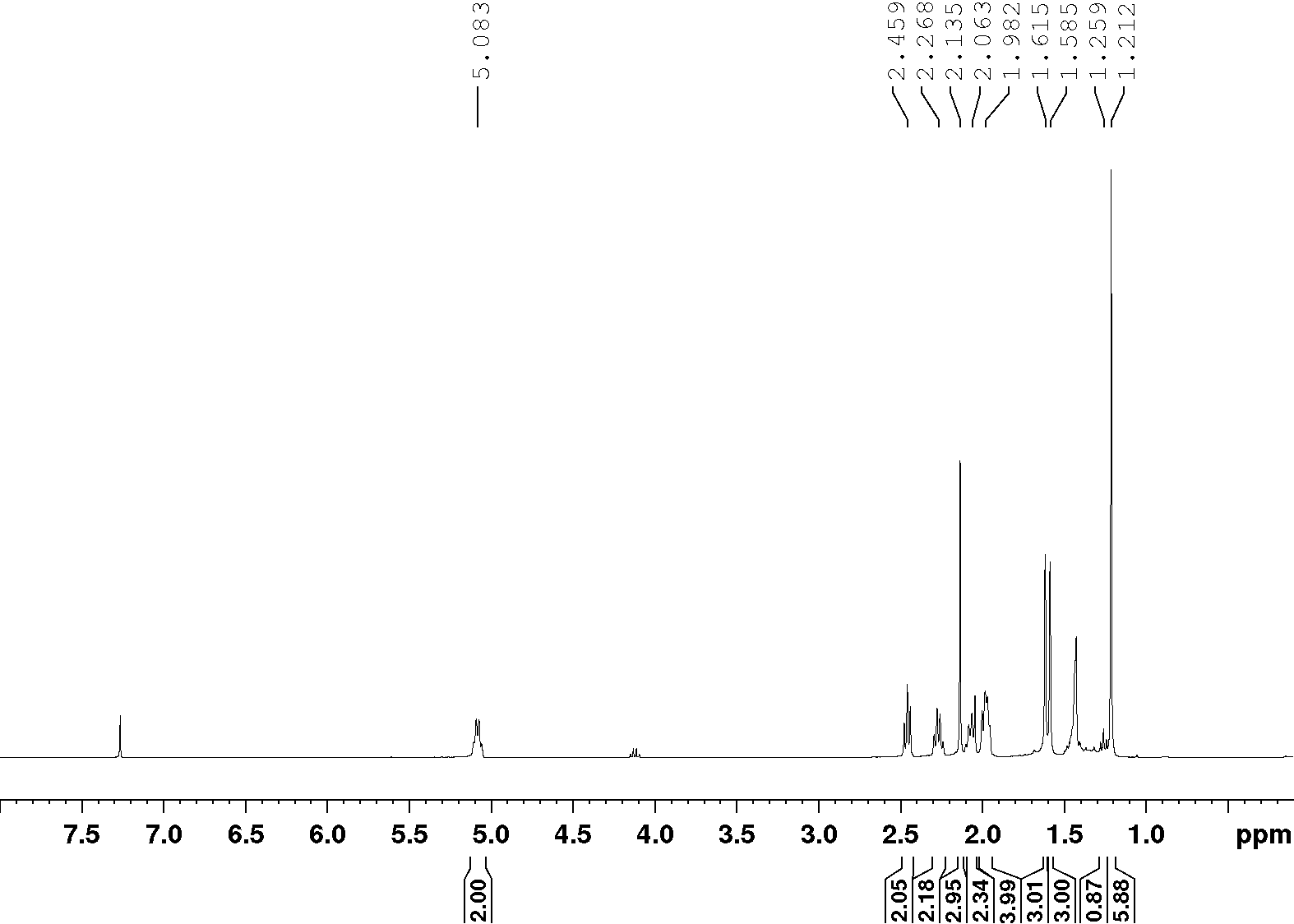


**^13^C-NMR**


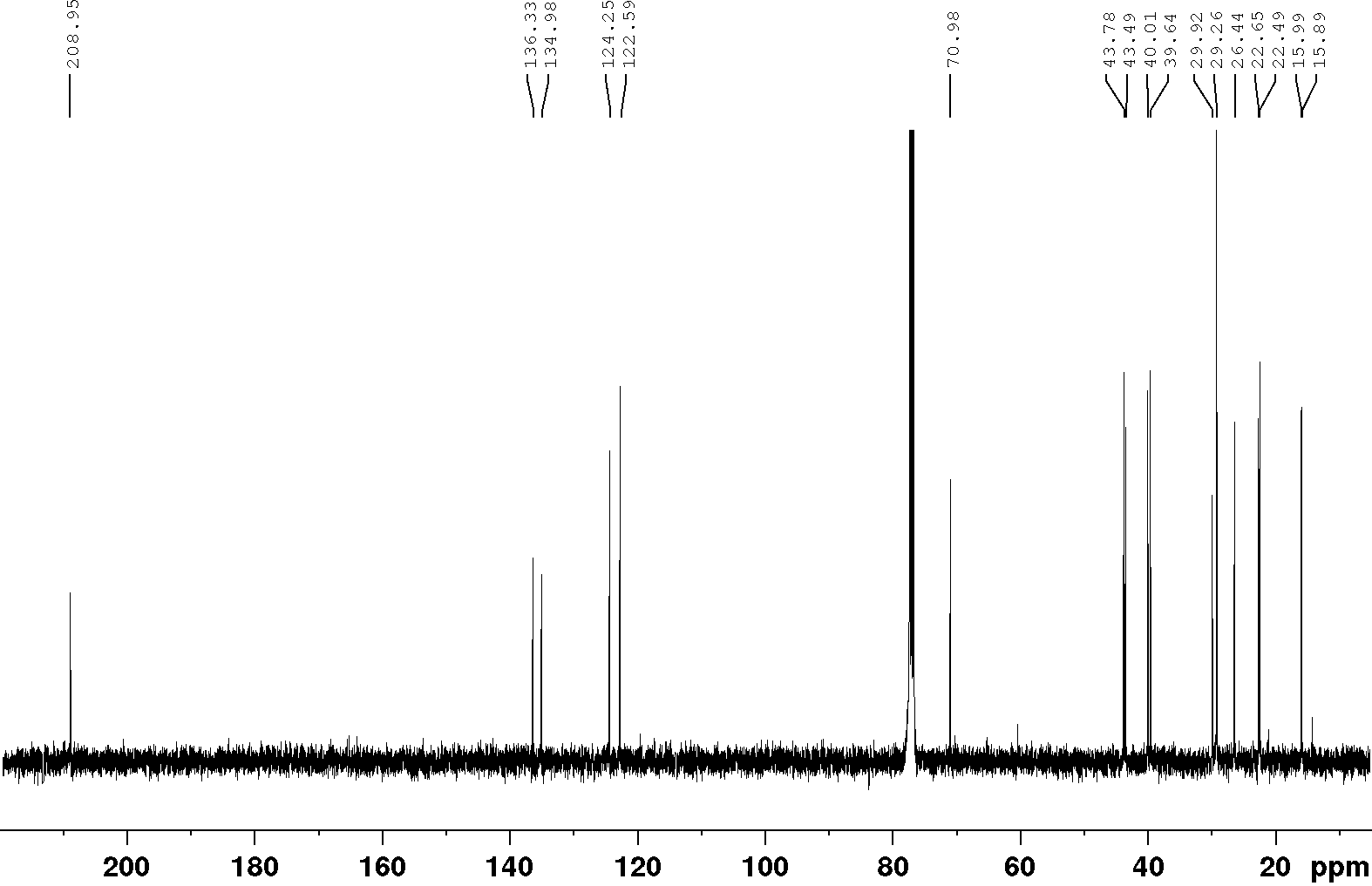


**Hydroxygeranylacetone 8:**

**^1^H-NMR**

**
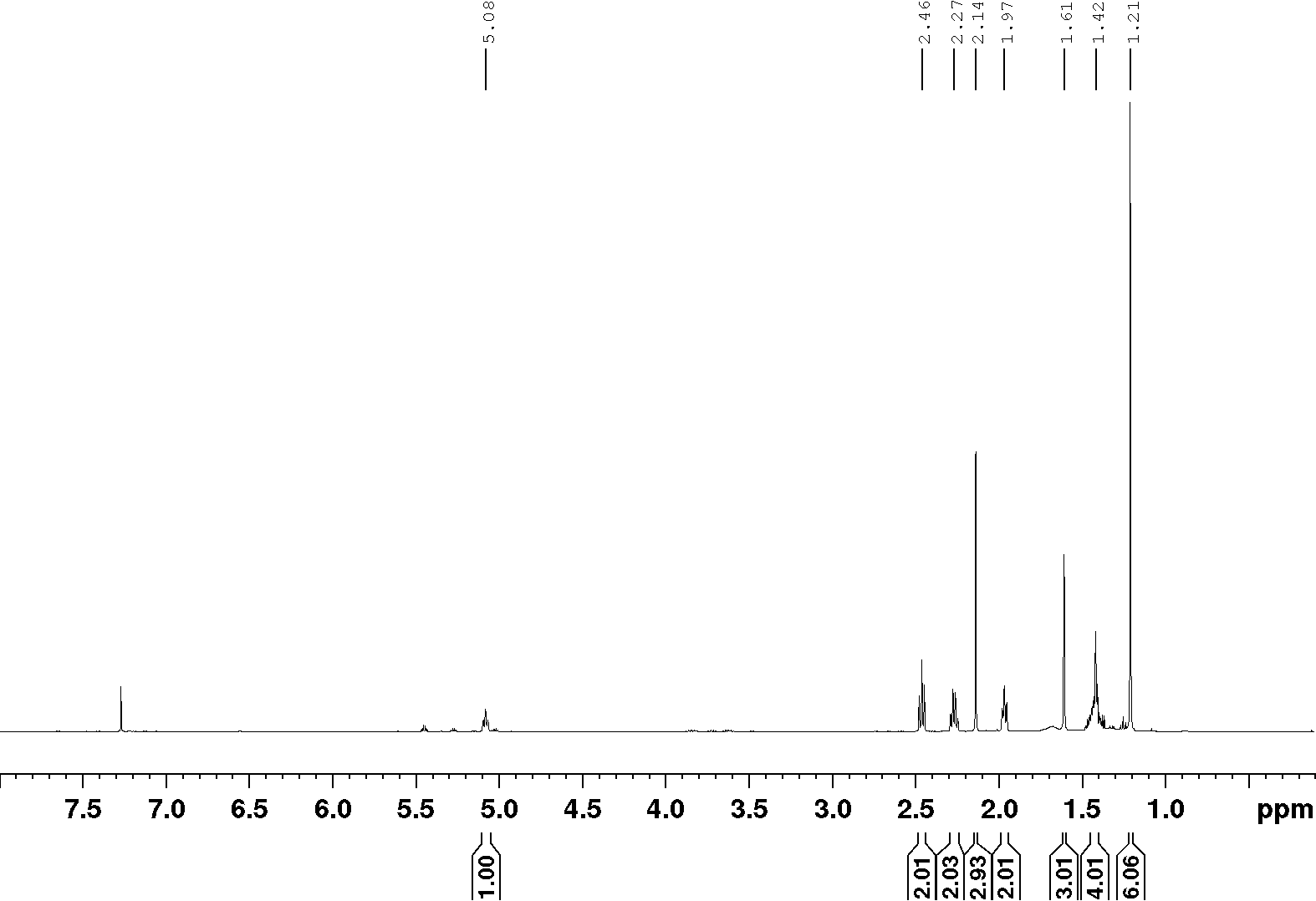
**

**^13^C-NMR**


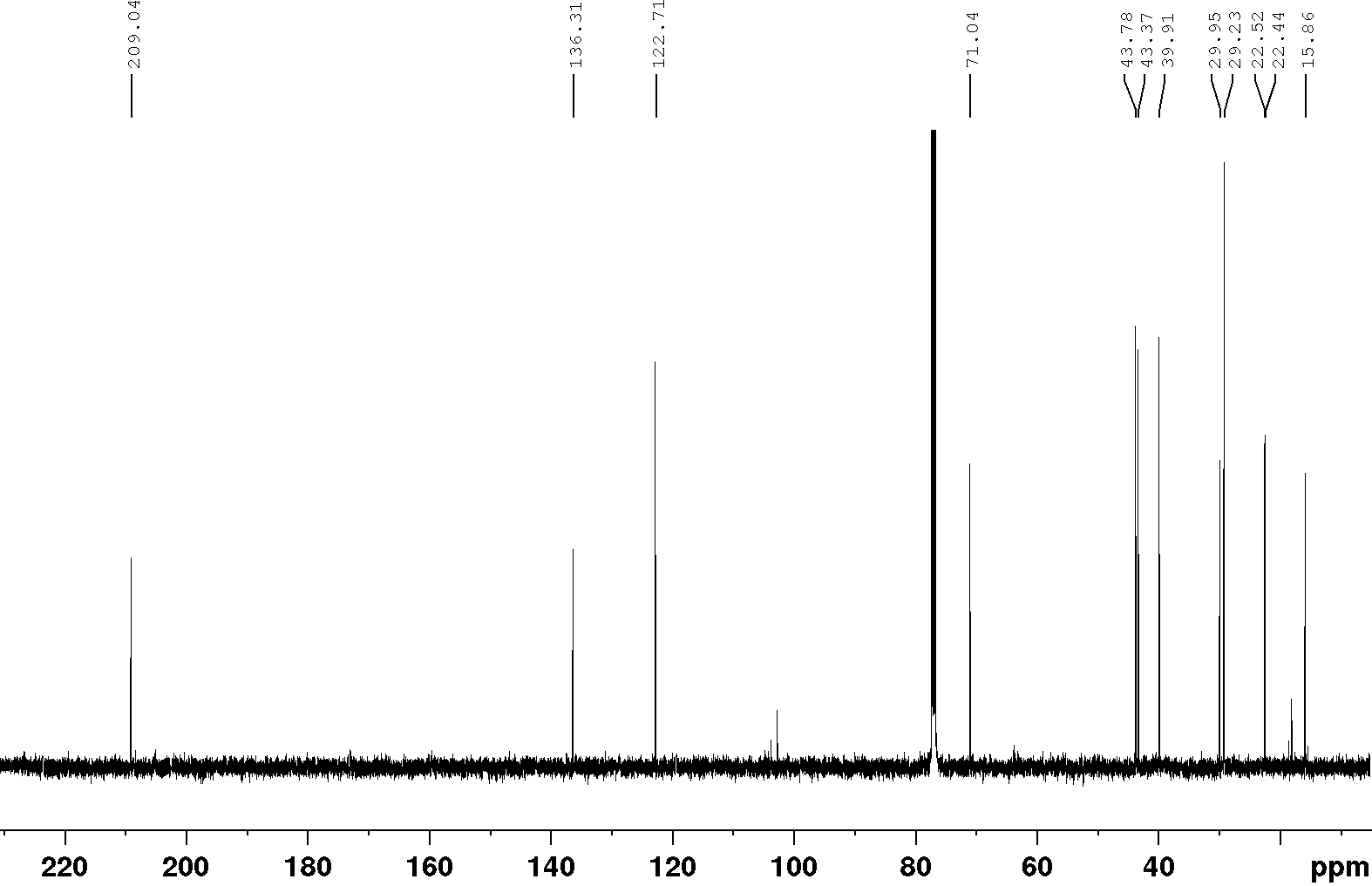


### **Hydroxypseudoionone 9:**

**^1^H-NMR**

**
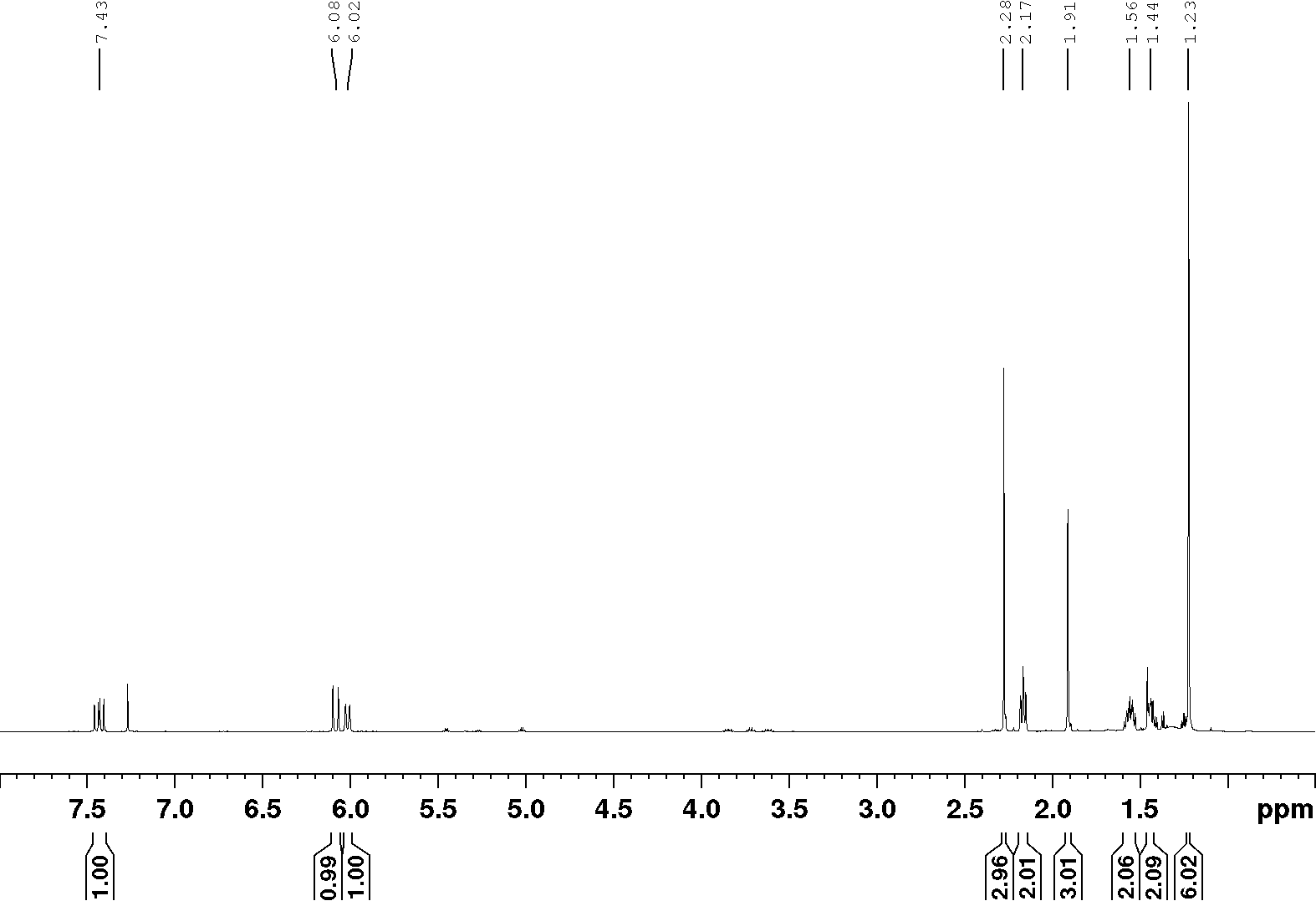
**

**^13^C-NMR**


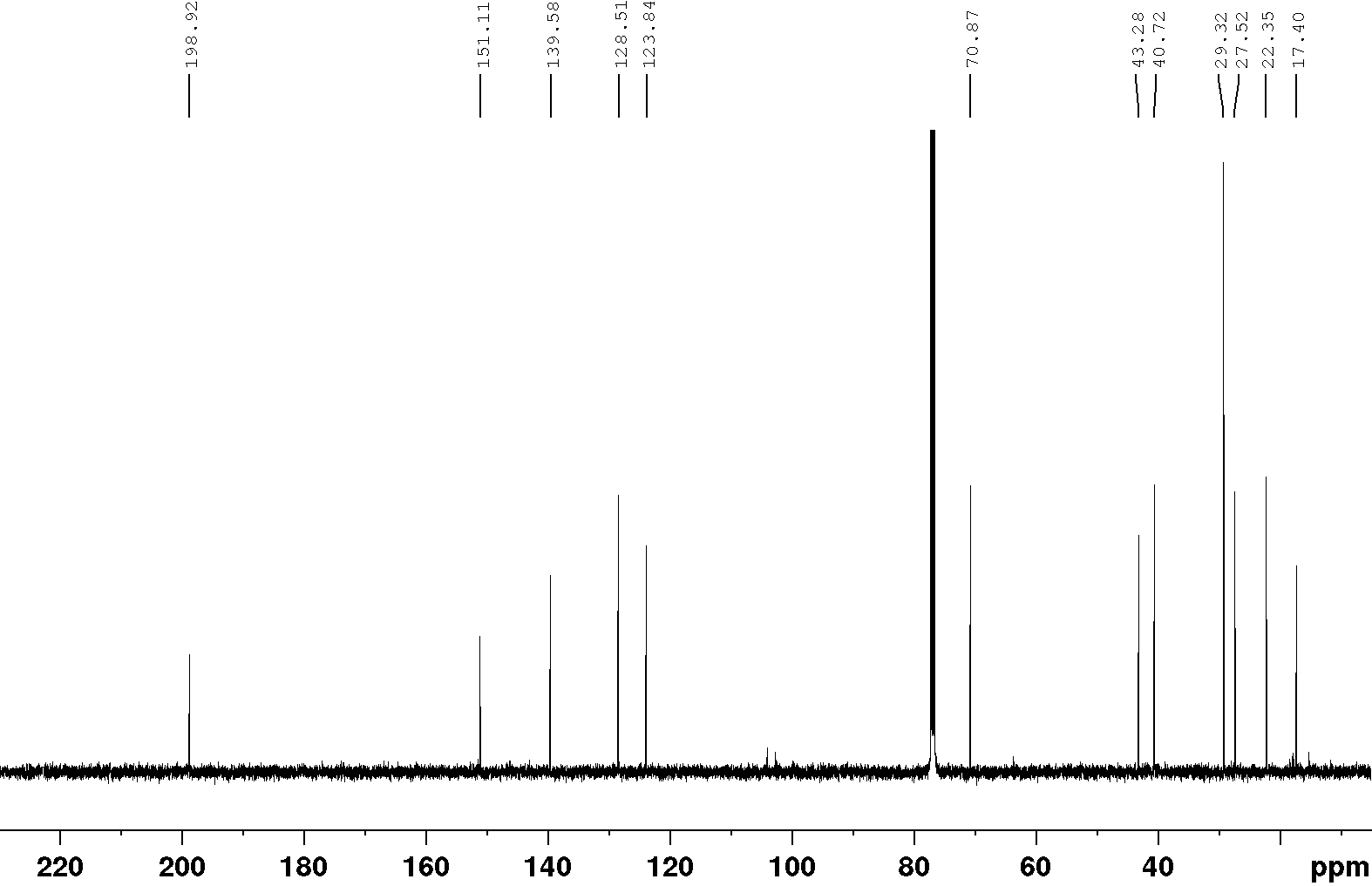


### **Hydroxyhomofarnesol 13:**

**^1^H-NMR**

**
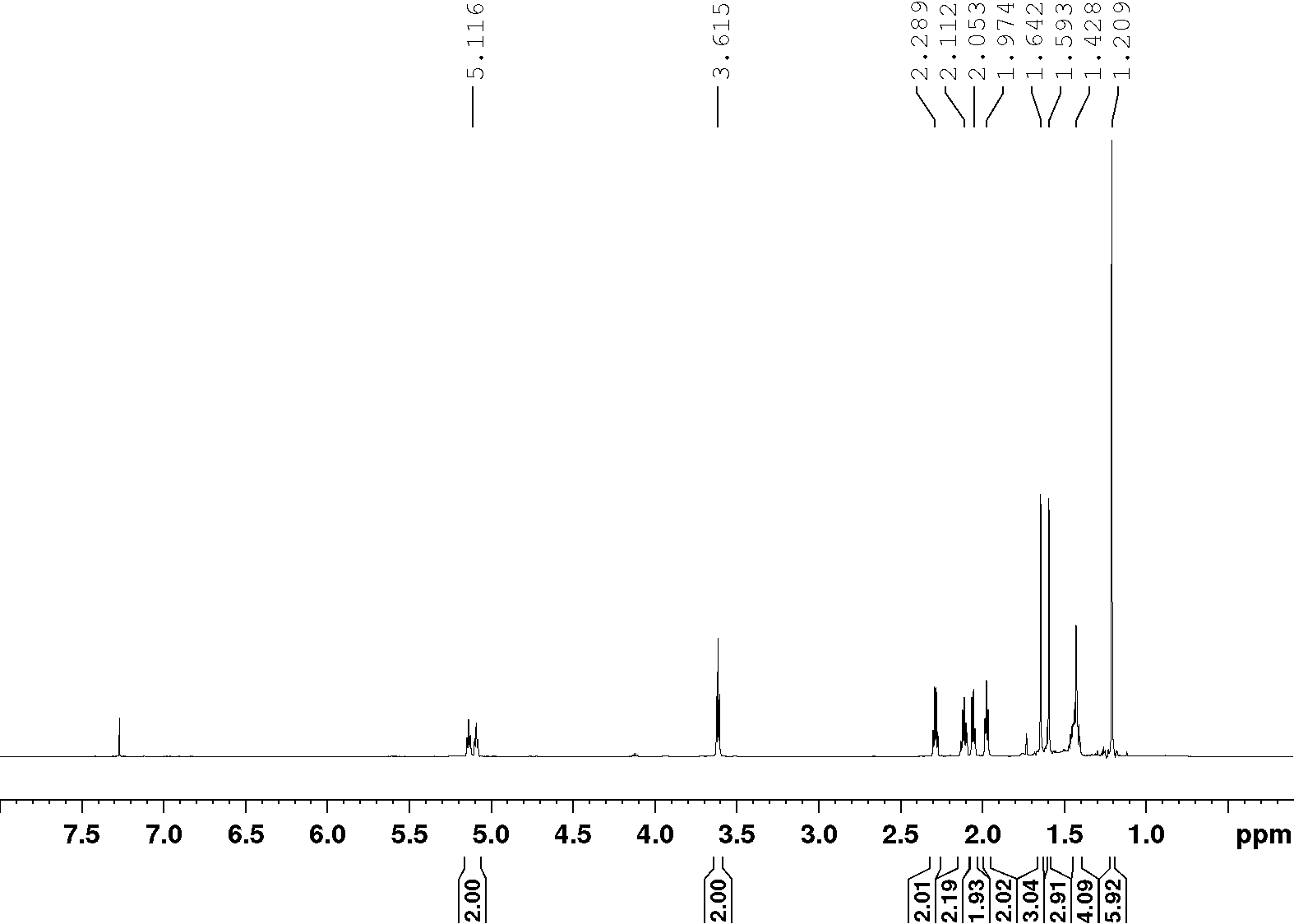
**

**^13^C-NMR**


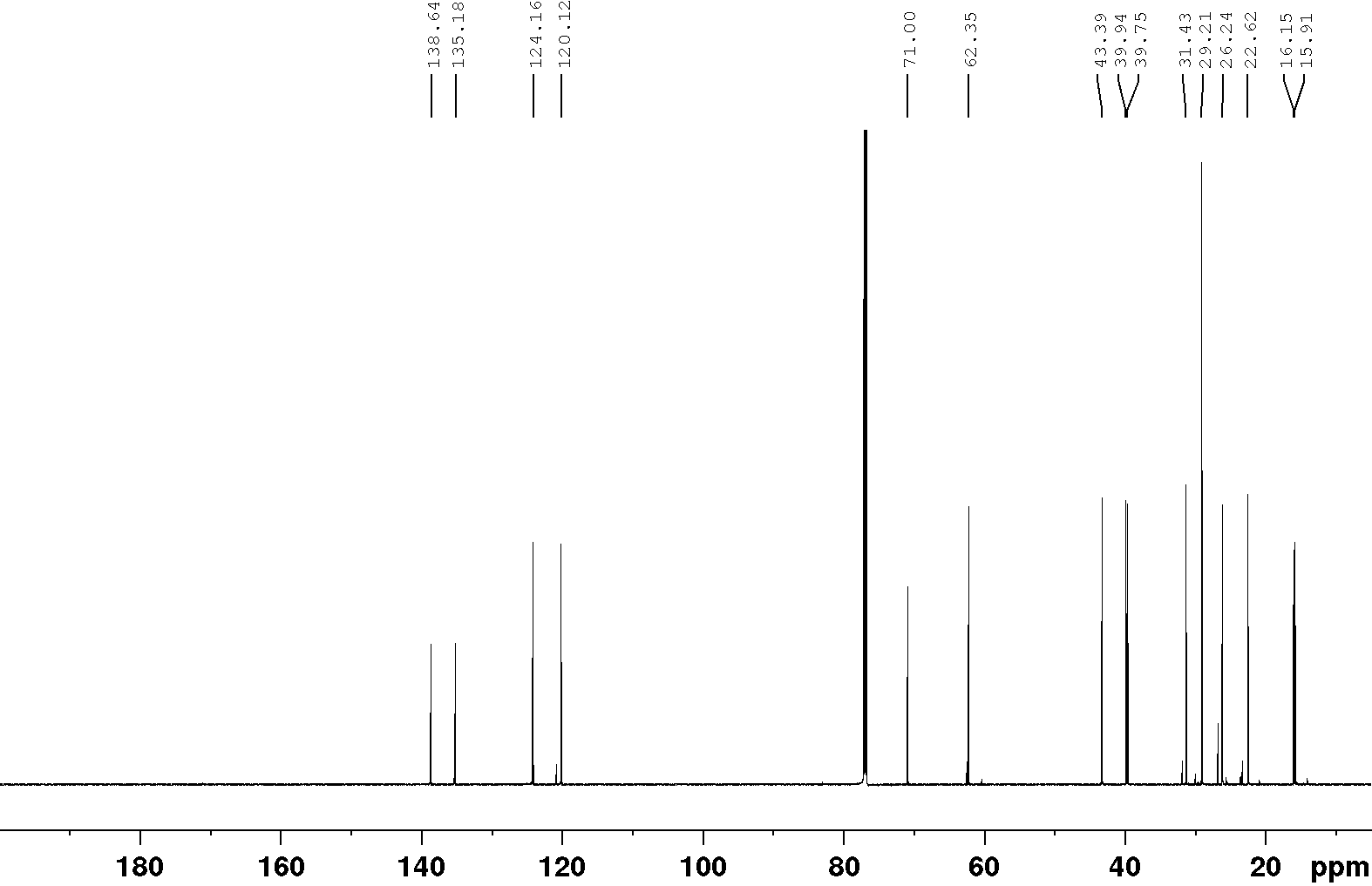


### **Hydroxyfarnesol 12:**

**^1^H-NMR**

**
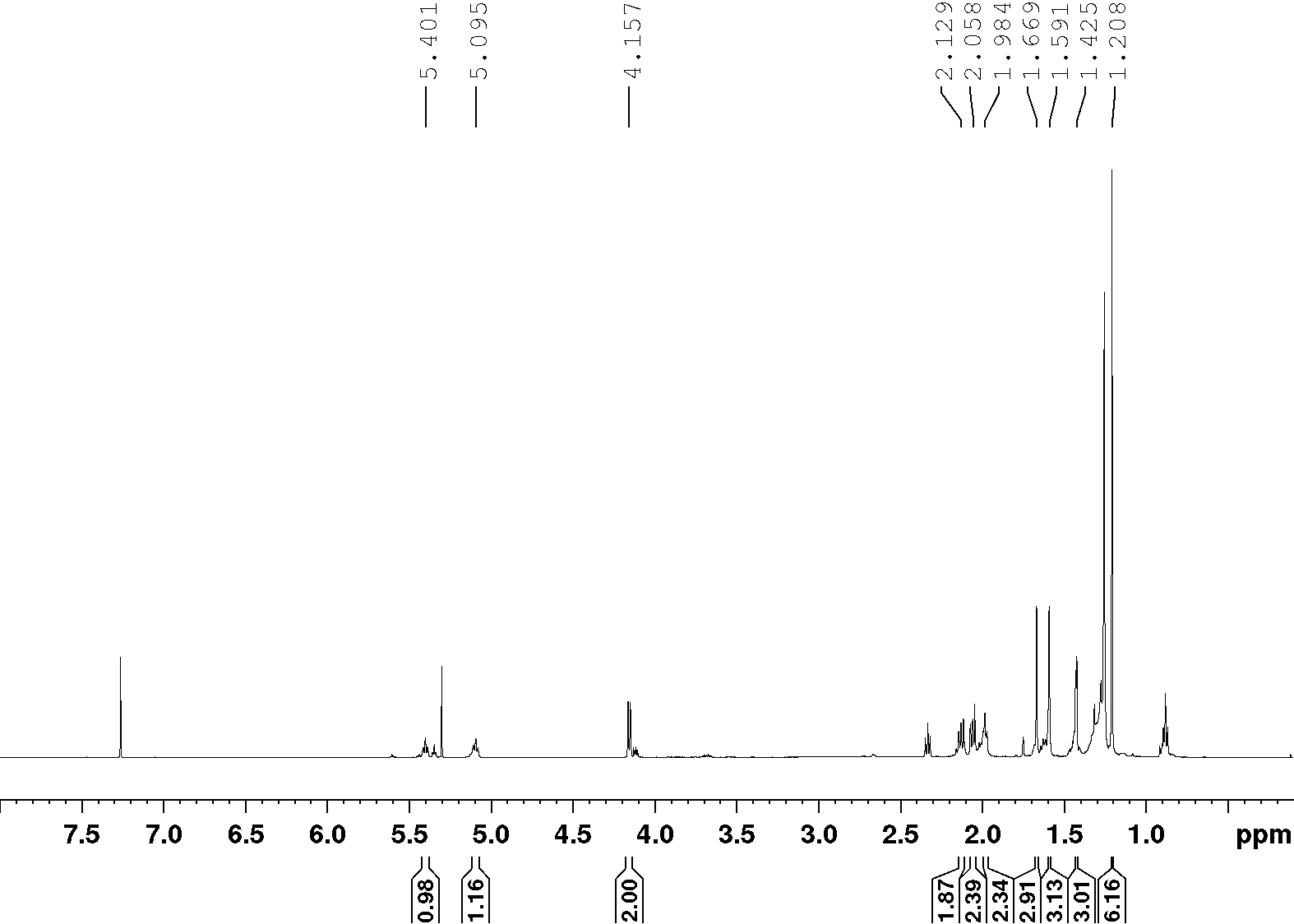
**

**^13^C-NMR**


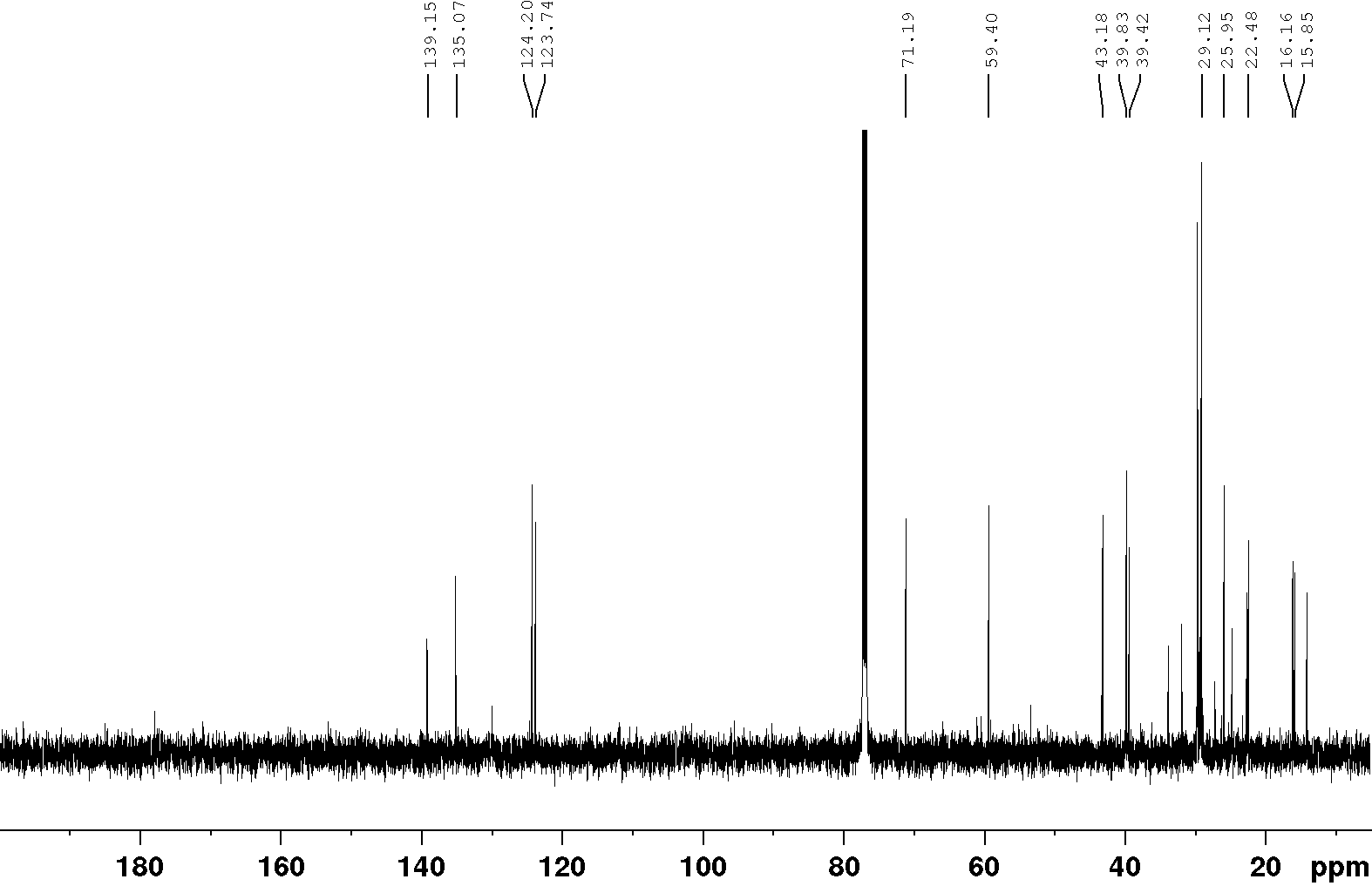


### **Hydroxygeranyllinalool 16:**

**^1^H-NMR**

**
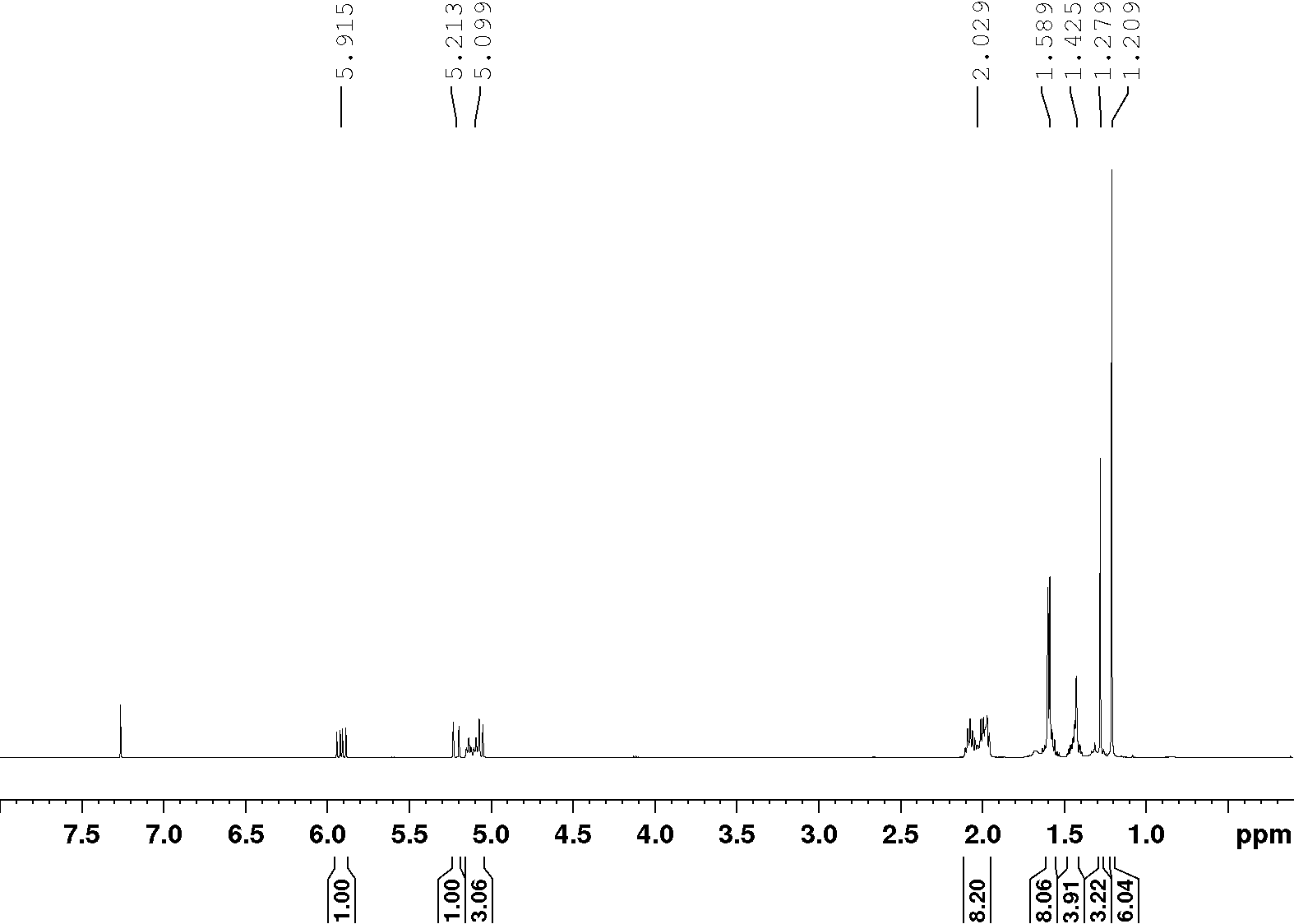
**

**^13^C-NMR**


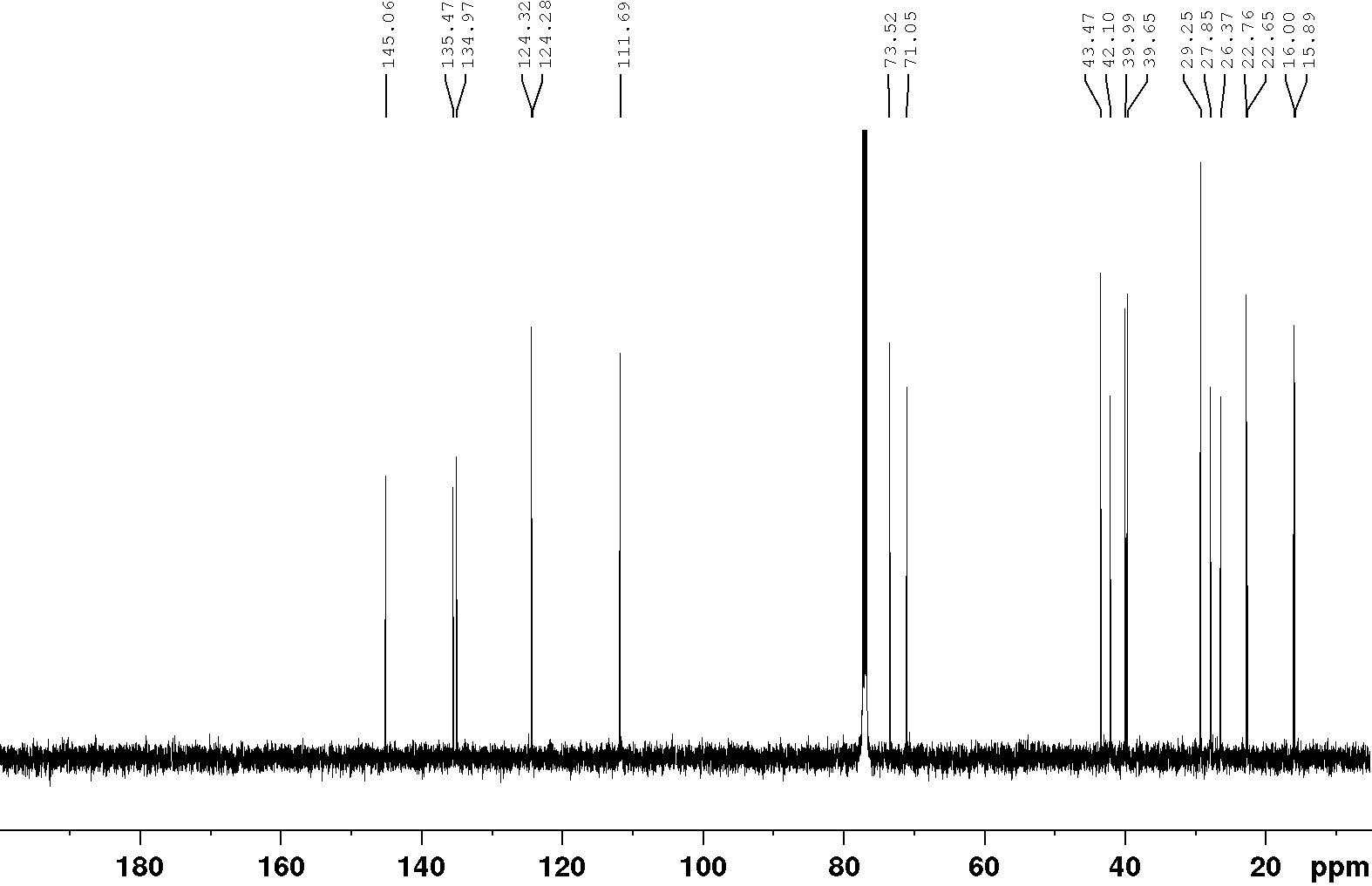


### Hydroxy-α-farnesene 23:

**^1^H-NMR**

**
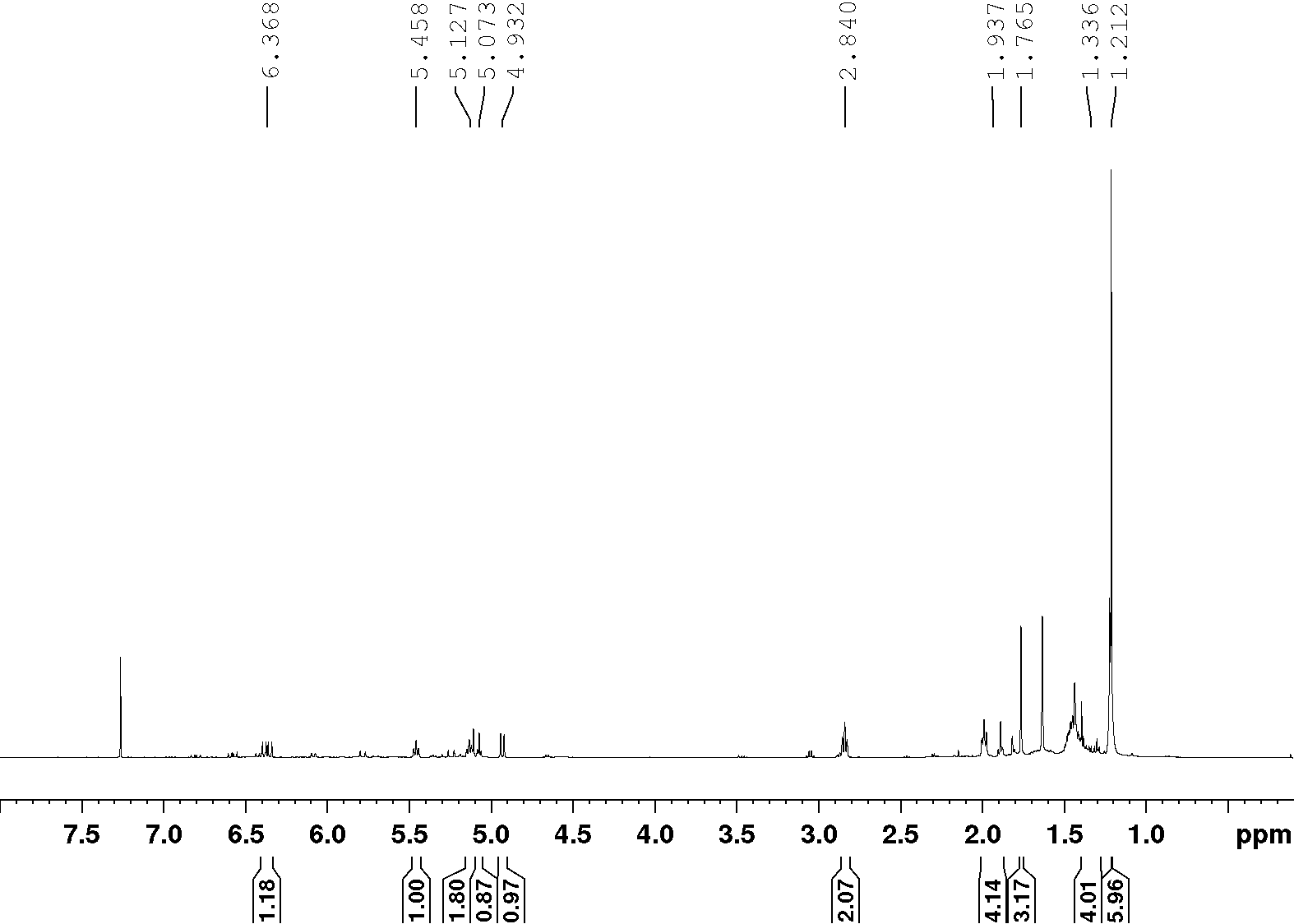
**

**^13^C-NMR**


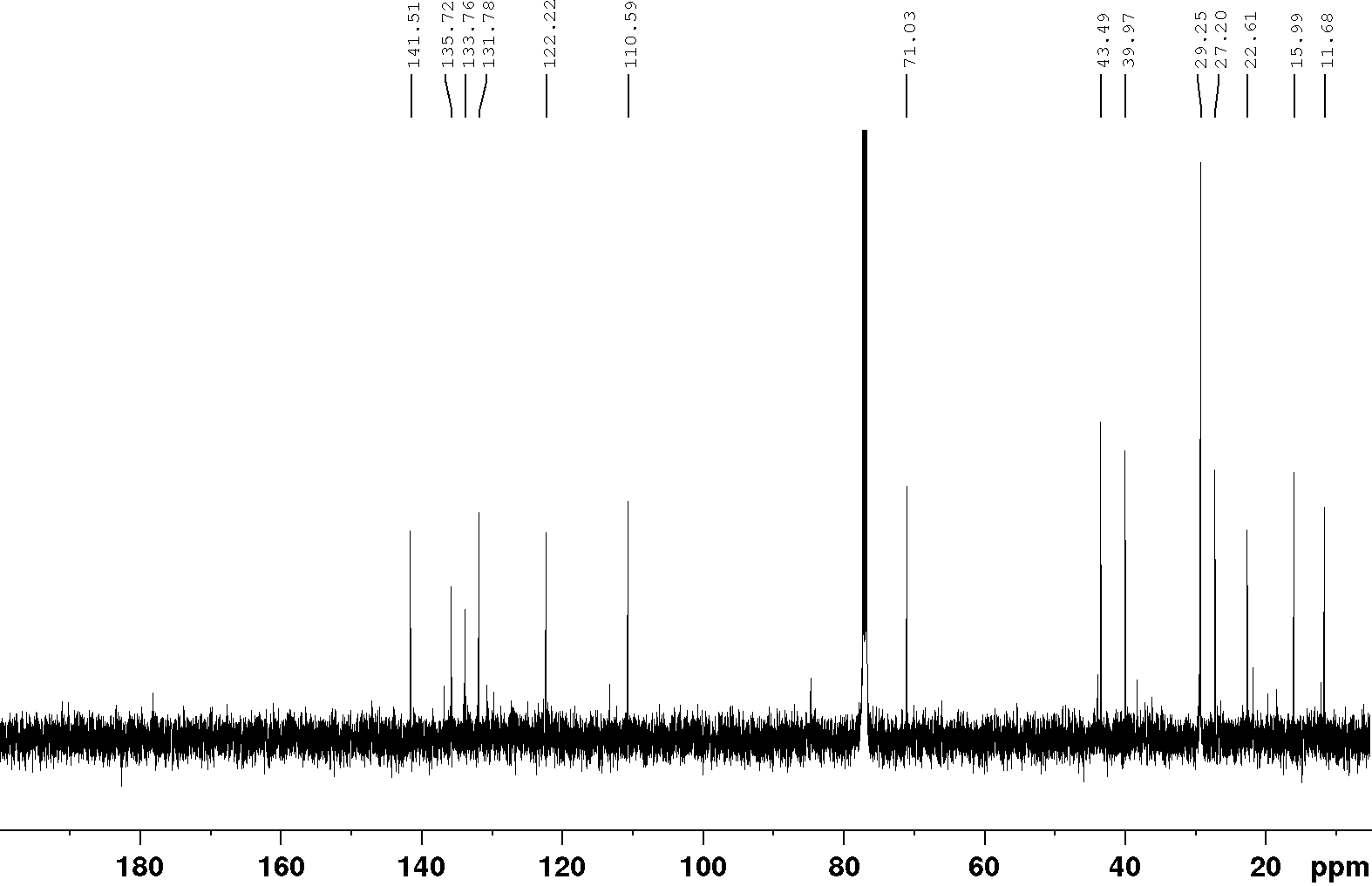


### Gernylisorpopanol:

**^1^H-NMR**

**
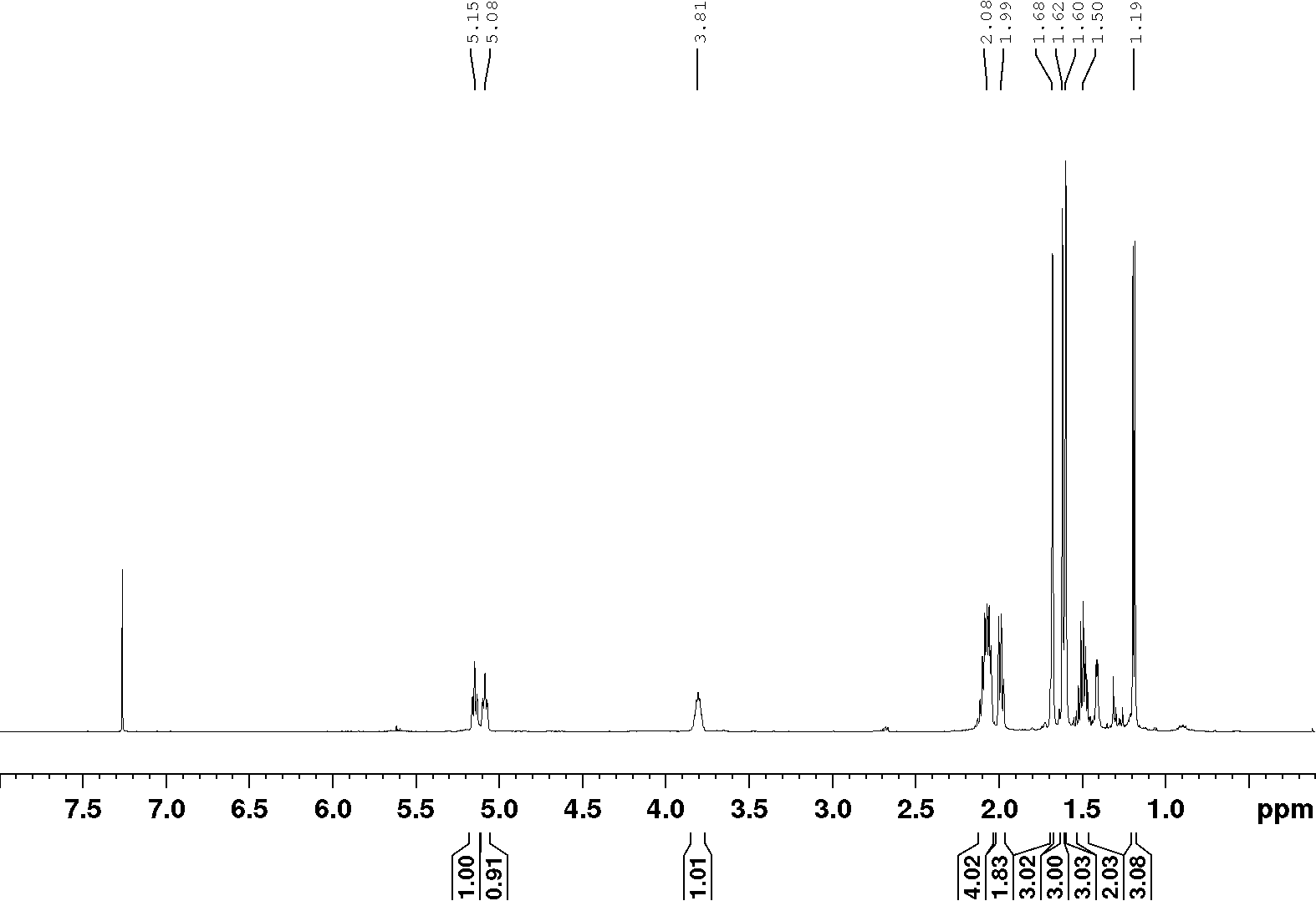
**

**^13^C-NMR**


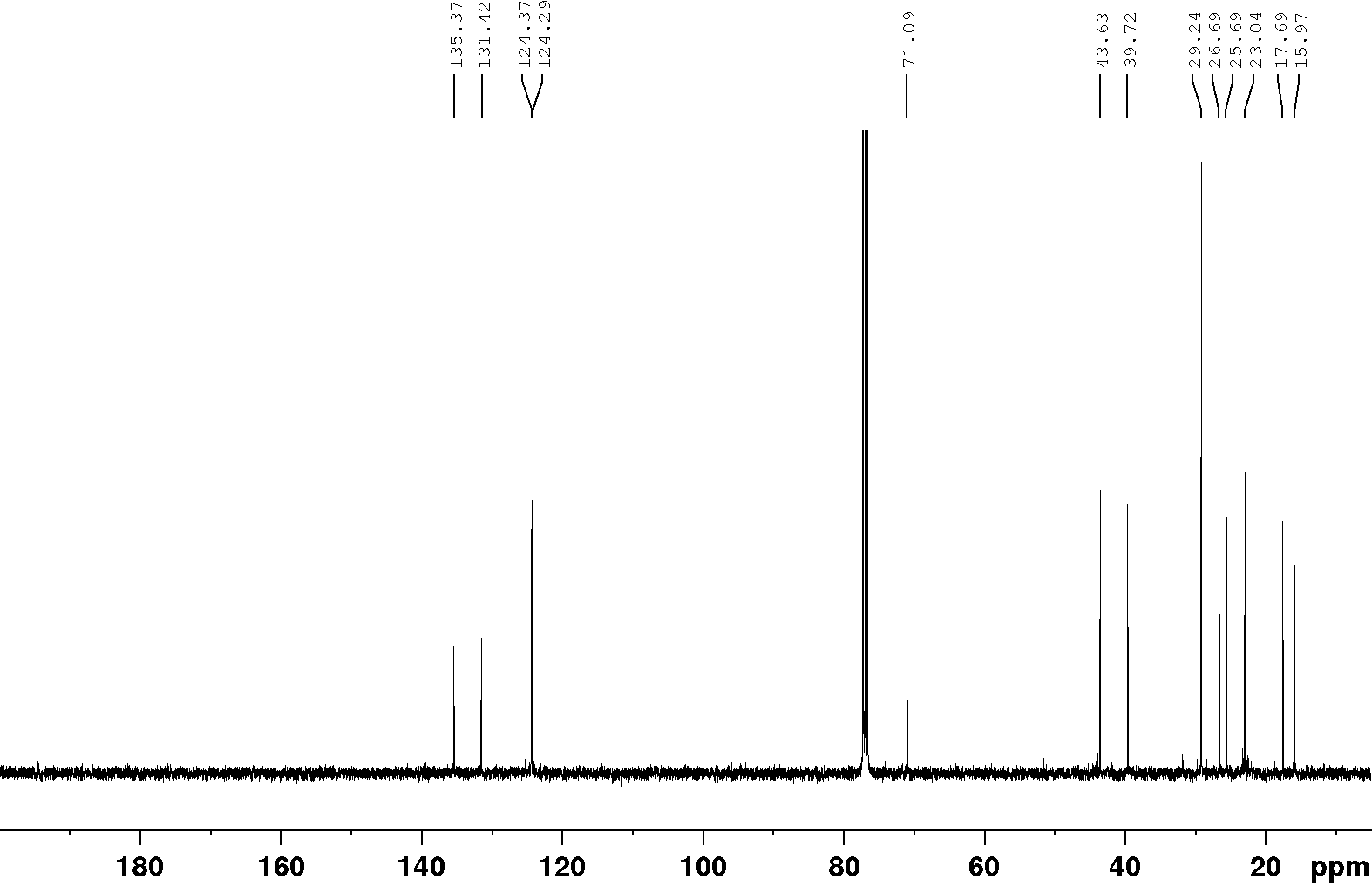


### (E)-5,9-dimethyldeca-4,8-dien-1-ol (calmusol):

**^1^H-NMR**

**
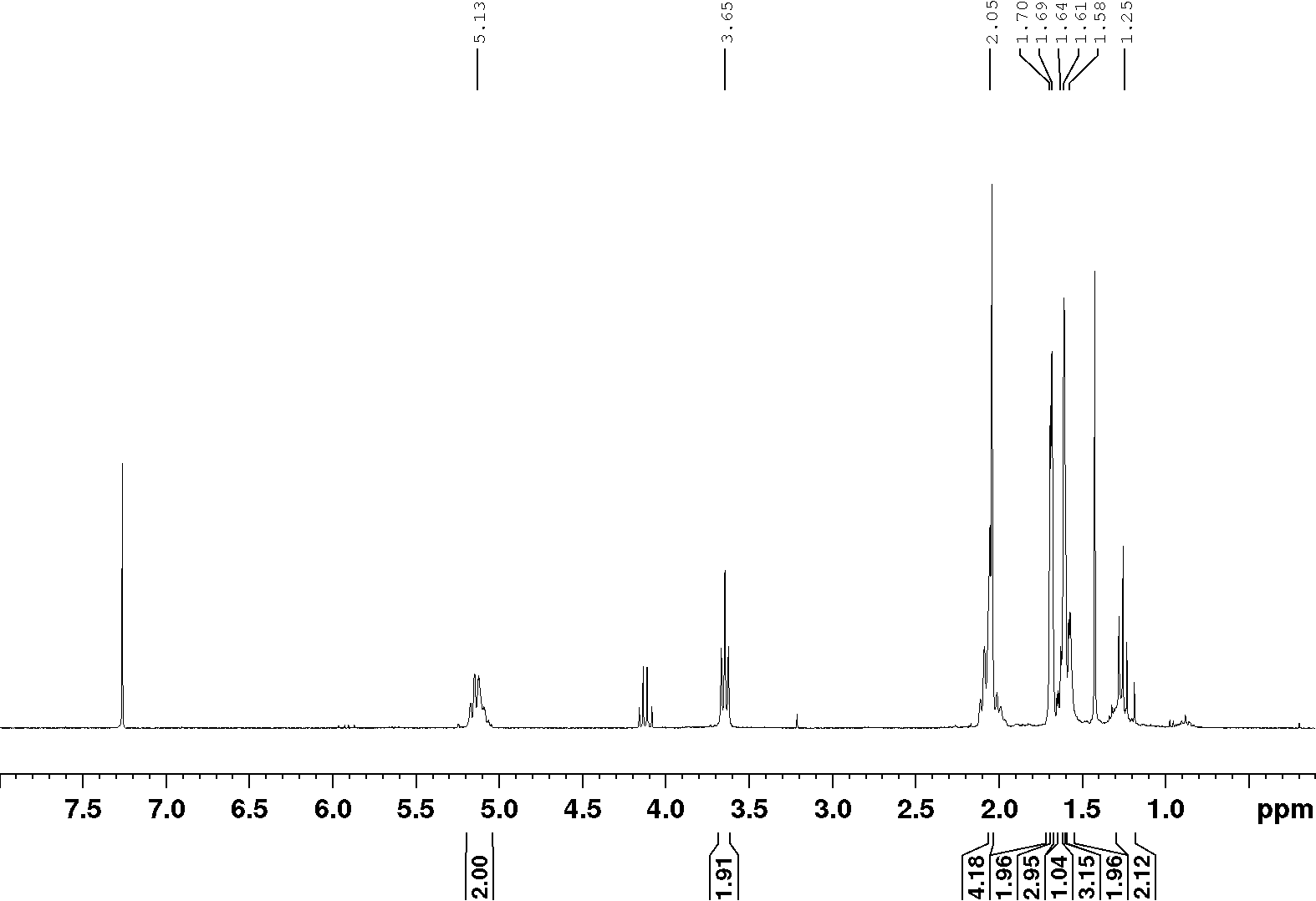
**

**^13^C-NMR**

**
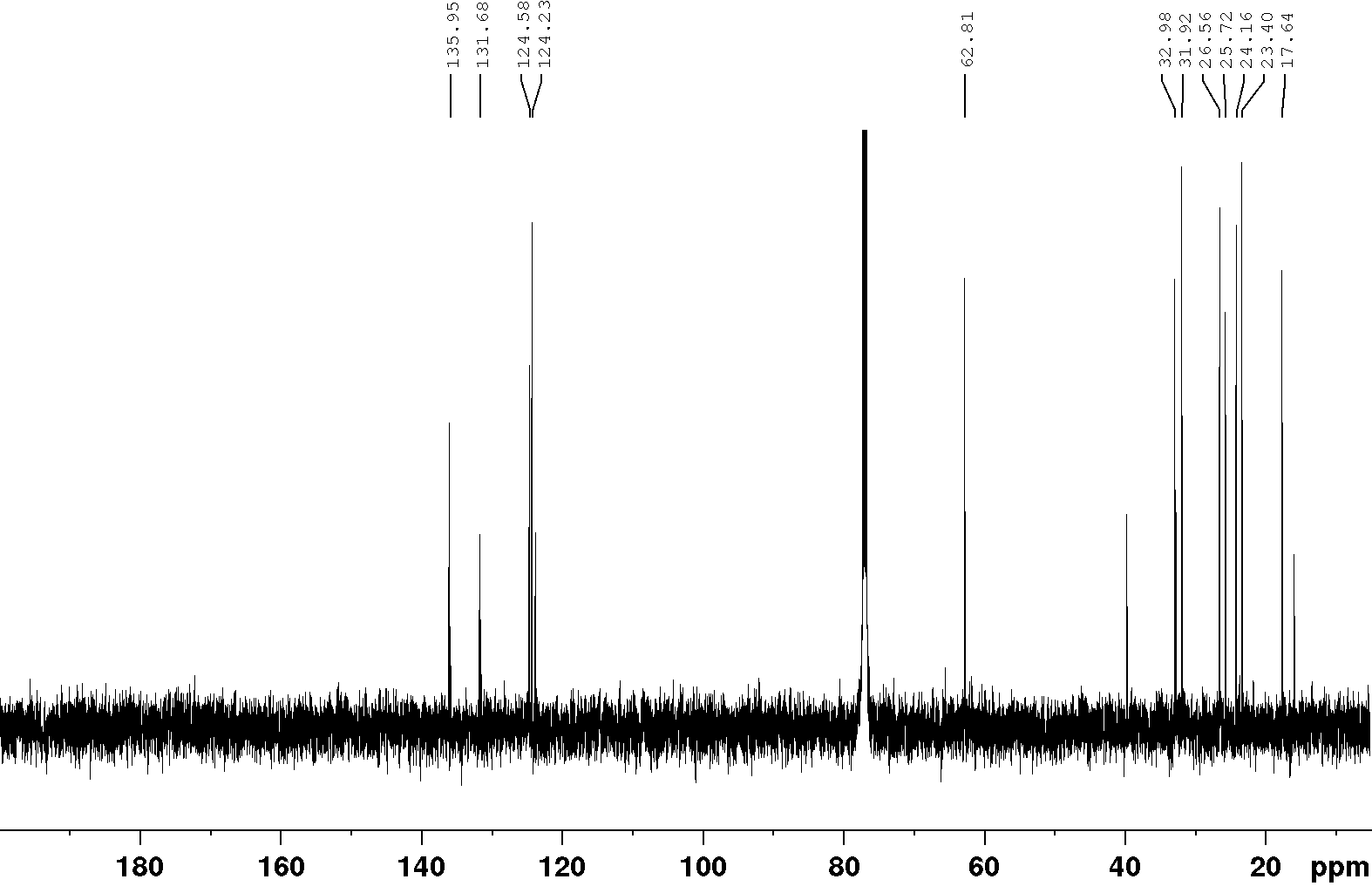
**

### Geranylthioacete:

**^1^H-NMR**

**
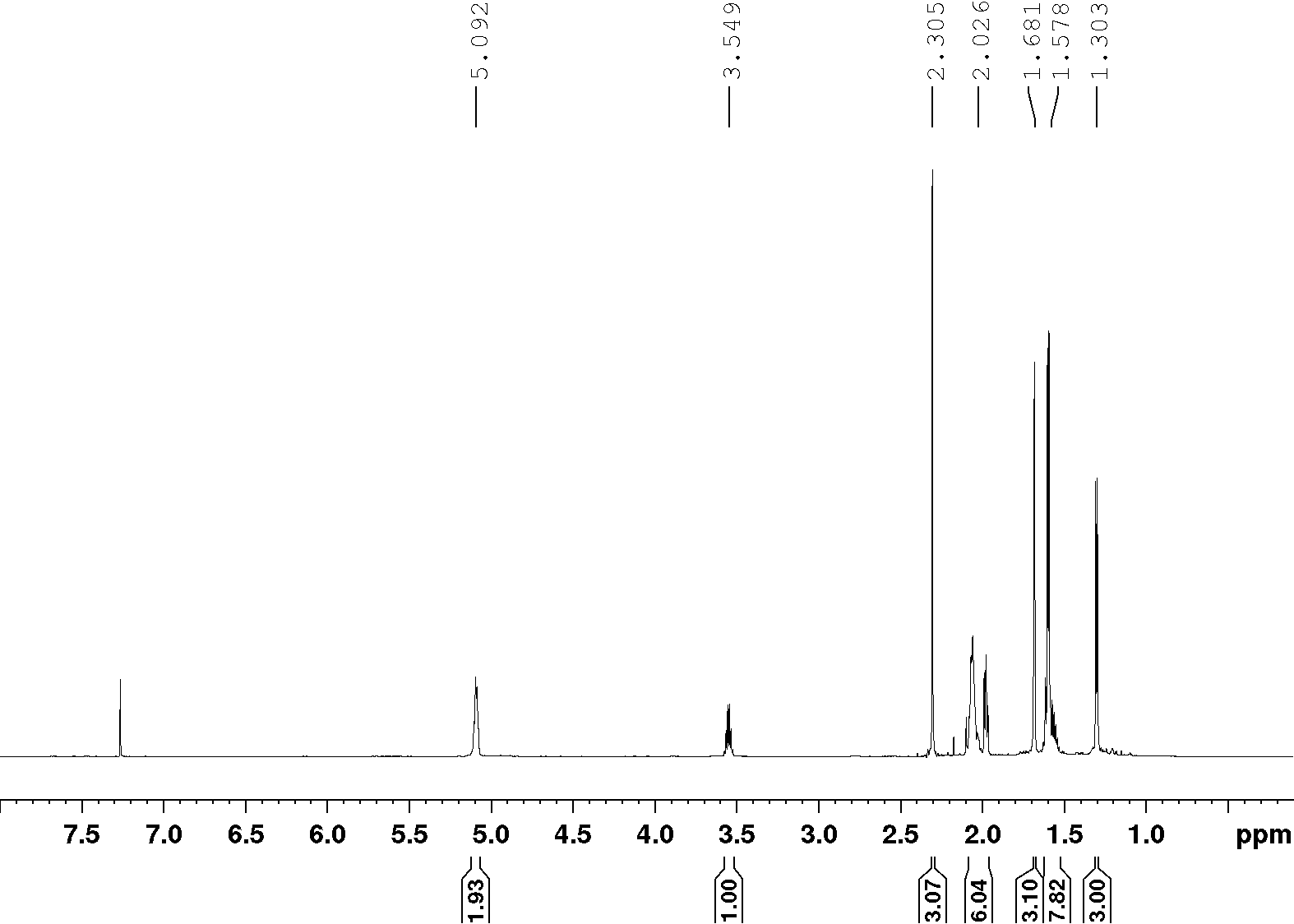
**

**^13^C-NMR**


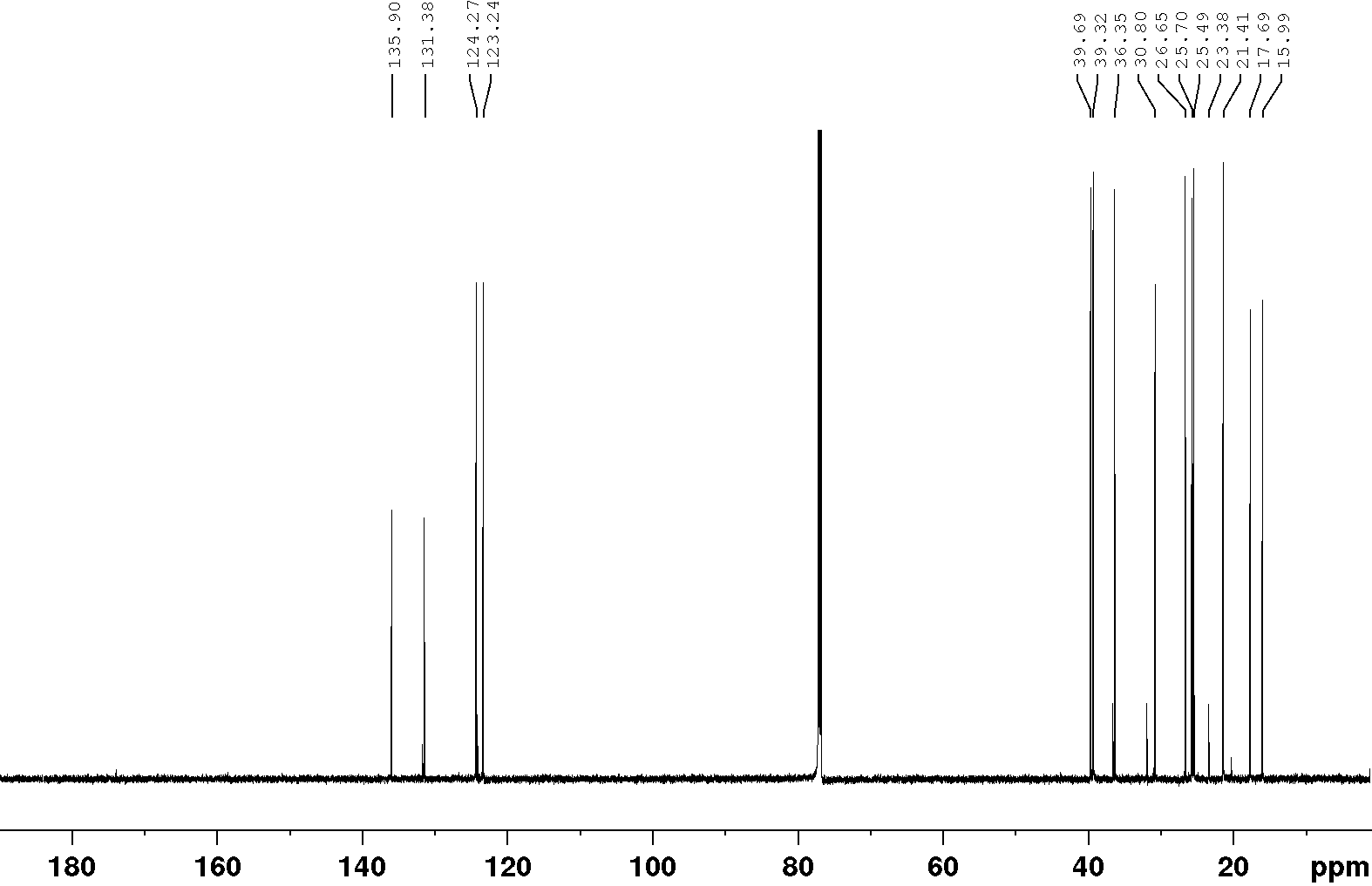


# Gen sequences and plasmid cards

## Carotenoid-1,2, hydratases

***Rg*CrtC IL144 WT:**

ATGCGTGCAGCAGAAAGCGGTGCAGATGCACGTGTTCGTCCGGATGATCGTCTGGAACCGGCAGATGCCCCTGCCGGTGATGCGGGTGAACTGCGTGCACCGGTTCCGGGTGATGGTGGTAGCGCAGTTCGTCCTGGTGATGCACGTCTGGATGTTCTGGTTCCGCCTGGTCTGGTTGATGAACCTGCAGCAGGTCCGGTTCCTGGTGGTCGTCAGCGTGCTCCGGGTGCAGGTCGTGCAGATGGTGGTGATGTGCGTCCGGCAGGCGGTCGTGATGCCGATGGTGCACCGCGTTTTGATCAGCCGGTTCCTCCGGGTGGTTATCTGTGGTGGTATGTTGATGCAGTTAGTGATGATGGTCGTCATGGTCTGACCTTTATTGCATTTGTTGGTAGCGTTTTCAGCCCGTATTATGCATGGGCTGGCGGTCCGAATGCAGATCGTGCCGATCCGGAAAATCATTGTGCACTGAATATTGCACTGTATGGTGATGCCGGTAAACGTTGGACCATGACCGAACGTGGTCGTCGTTGGATGCGTCGTAGCCGTGATGAATTTGTTATTGGTCCGAGCCGTCTGCATTGGGATGGTGAAAGCCTGCTGGTTGAATTTGATGAAATTGGTGTTCCGATTCCGCGTCGTGTTAAAGGTCGTGTTCGTGTTTGGCCGAAAGCACTGTGTCGTTTTGTTACCAGCCTGGATAGCGGTGGTCGCCATCGTTGGGGTCCGATTGCACCGTGTAGCCGTATTGAAGTTGAACTGGATAGCCCGAATGCACGTTGGAGCGGTCATGCCTATGTTGATAGCAATGAAGGTGATGAACCGATTGATCGTCCGTTTCGTGAATGGGATTGGAGCCGTGCAACCATGGCAGATGGTTCAACCGCAGTTATTTATGATGTTCGCCAGAAACGTGATGGTGATCGTGTTATTGCAGAACGTTTTCTGCTGGATGGTAGCACCGAAAGCTTTGAAGCACCGCCTCGTCAGCCGCTGCCGACCACACTGTGGCGTATTGATCGTACCATGCGTACCGAACCGGGTGTTCCGGCATTTGTGGAACAGACCCTGGAAGATACCCCGTTTTATGCACGTAGCATGGTTCGTAGCGGTCTGCTGGGTGAAGTTGTGACCAGCGTTCATGAAACCATGCTGCTGCCACGTGTTATTACACTGCCGGTGCGTCTGATGCTGCCGTGGCGTATGCCTCGTCGTAGCTAA

***Rg*CrtC S1 WT:**

ATGCGCGCAGCGGAAAGTGGAGCAGACGCACGTGTTCGTCCAGTAGATCGTGTTGAACCTGCGGATGCGCCCGCGGGCGATGCAGGCGGCCTGCGTGCCGCGGTGCCAGGTGATGGCGGCTCAGCAGTGCGTCCTGGAGATGCGCGTTTAGATGTTCTTGTGCCGCCAGGGTTGGTTGATGAACCAGCAGCAGGCGCCCTGCCGGGCGGTGGCCAACGTGCCCCGGGTGCTGGACGCGCAGACGGTGGAGACGTACGCCCTGTAGGTGGACGCGATGCAGACGGTGCCCCGCGTTTTGATCAGCCTGTACCACCAGGCGGCTACCTGTGGTGGTACGTTGATGCAGTGTCAGATGATGGACGTCACGGACTCACGTTTATTGCGTTTGTGGGATCCGTGTTTAGCCCGTACTATGCATGGGCGGGCGGTCCAAAAGCGGATCGCGCAGATCCTGAAAATCATTGTGCCTTGAATATTGCATTGTATGGCGATGCGGGTAAACGTTGGACCATGACGGAGCGTGGACGCCGTTGGATGCGTCGTTCGCGTGATGAATTTGTGATTGGTCCTTCTCGTTTACATTGGGATGGTGAAAGTTTACTGGTAGAATTTGATGAAGTGGGTGTTCCCATTCCTCGTCGTGTAAAAGGGCGTGTCCGTGTGTGGCCAAAAGCACTTTGTCGTTTTGTTACTTCTTTAGATTCTGGTGGGCGTCACCGTTGGGGTCCTATTGCCCCCTGTTCCCGTATTGAAGTAGAGTTAGATTCACCTCGCGTTCGTTGGTCTGGACATGCGTATCTGGATTCAAATGAGGGAGATGAACCCATTGATCGTCCTTTTCGTGAATGGGATTGGAGTCGTGCAACCATGGCGGATTCGTCGACAGCCGTCATTTATGATGTTCGTCAAAAGCGTGATGGAGACCGTGTCATTGCGGAACGCTTCTTATTGGATGGTTCTACTGAATCCTTTGAAGCTCCACCCCGTCAACCTCTTCCAACCACCTTGTGGCGTATTGGACGTACCATGCGCACGGAACCGGGTGTTCCTGCCTTAGTGGAACAAACTCTTGAAGATACCCCATTTTATGCCCGCAGCATGGTTCGCTCAGGCTTGCTTGGAGAAGTTGTTACGAGCGTTCATGAAACTATGTTGCTCCCCCGTGTAATTACCCTTCCTGTCCGCCTTATGCTTCCGTGGCGTATGCCACGTCGTGCCTAA

## Plasmid cards


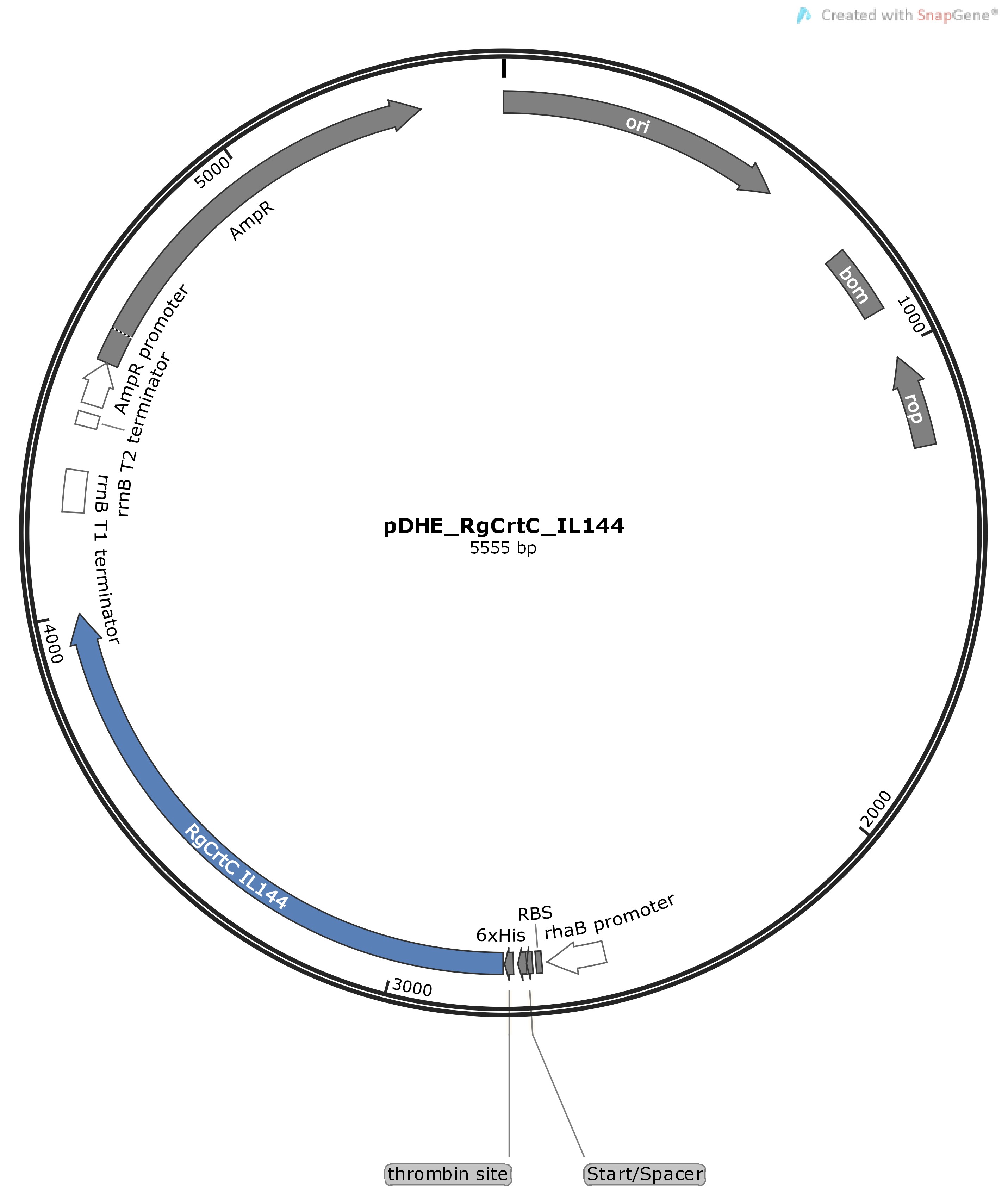


Figure S44: Plasmid card of pDHE_*Rg*CrtC_IL144. The plasmid contains an ampicillin resistance and a rhamnose promoter. Plasmid card was generated by using SnapGene 3.1.4 software from GSL Biotech LLC (California, USA).


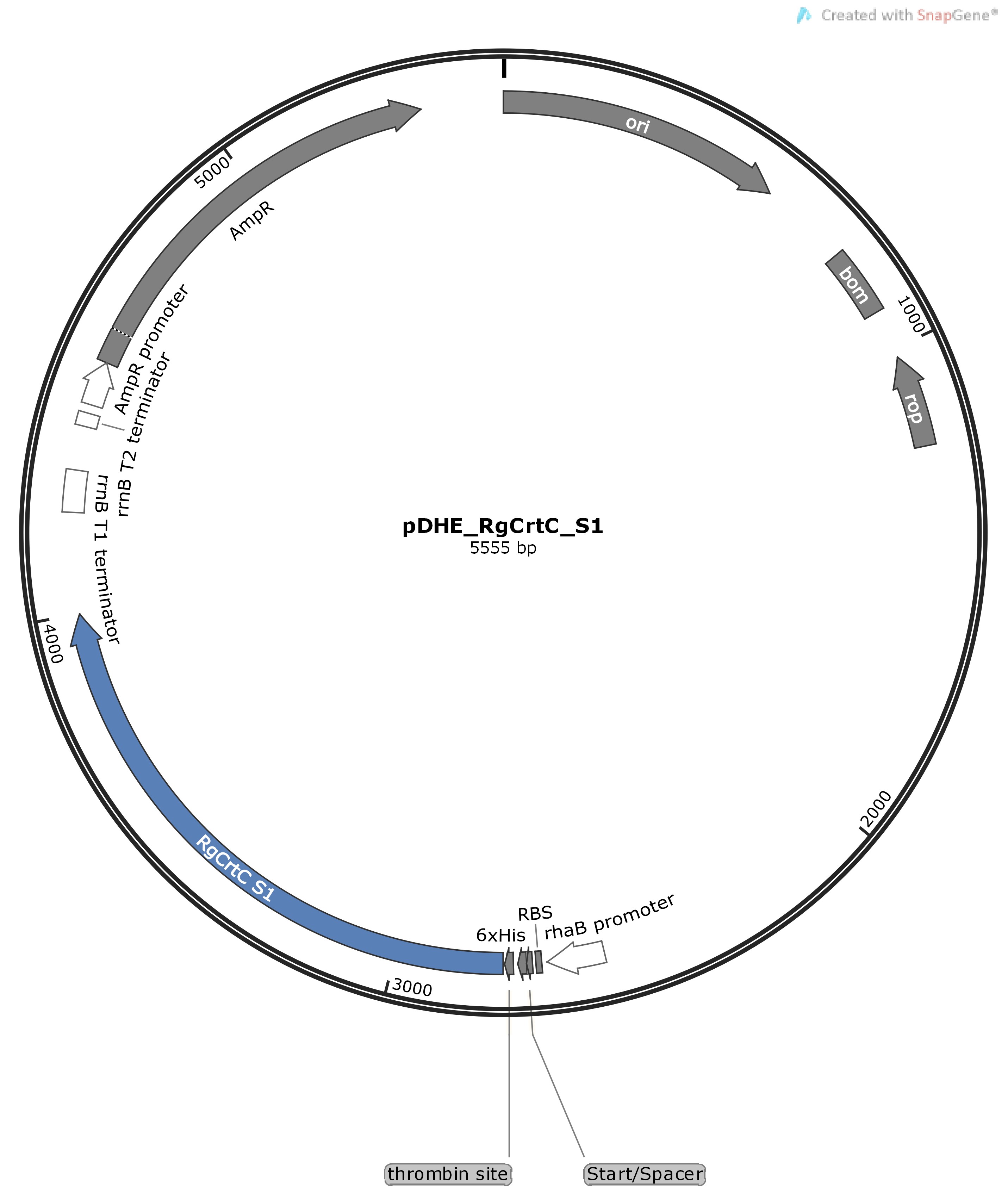


Figure S45: Plasmid card of pDHE_*Rg*CrtC_S1. The plasmid contains an ampicillin resistance and a rhamnose promoter. Plasmid card was generated by using SnapGene 3.1.4 software from GSL Biotech LLC (California, USA).


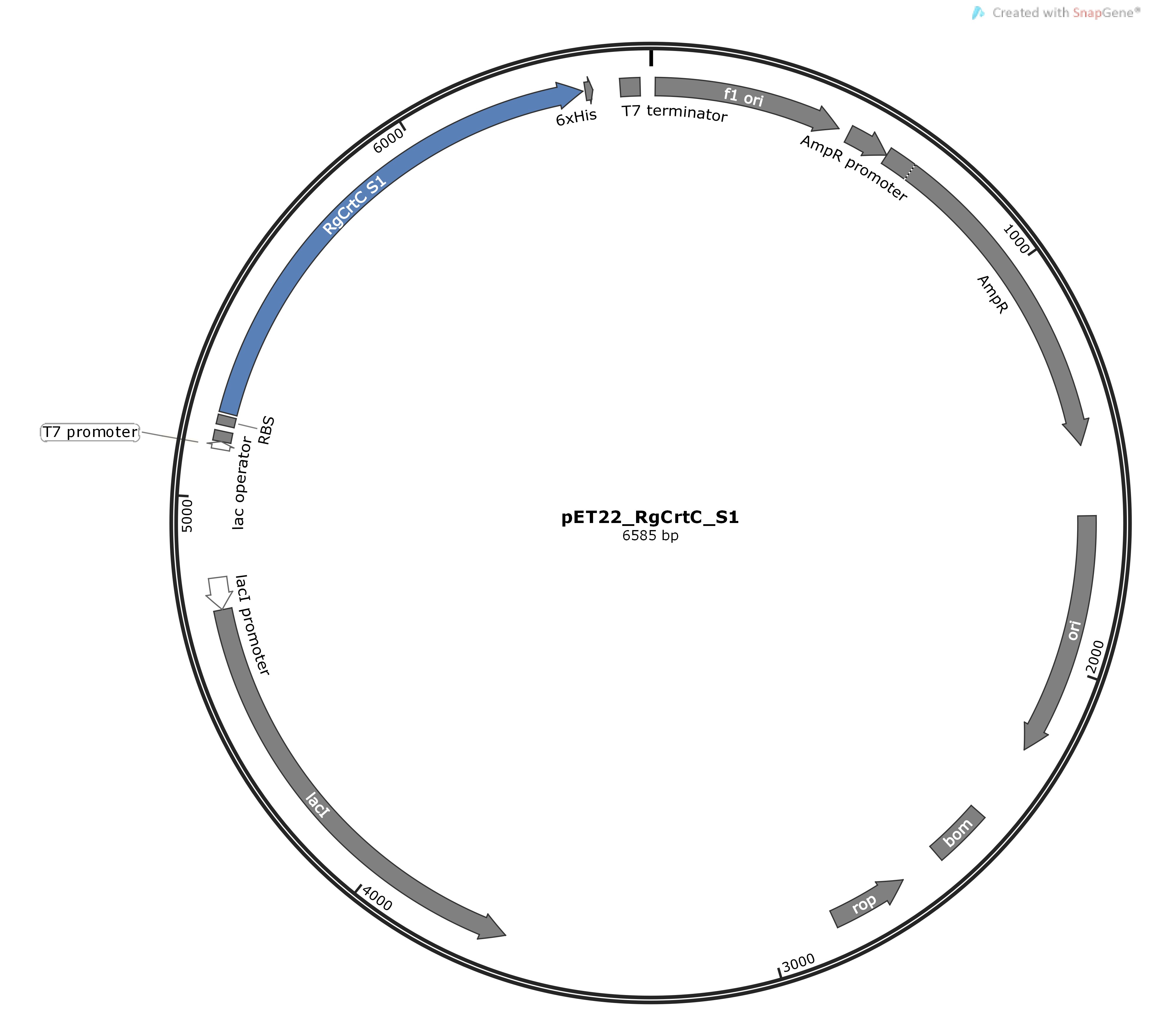


Figure S46: Plasmid card of pET22b(+)_*Rg*CrtC_S1. The plasmid contains an ampicillin resistance, a *lac* operon and a T7 promoter. Plasmid card was generated by using SnapGene 3.1.4 software from GSL Biotech LLC (California, USA).

[1] A. Alchenberger, C. Berbez, C. Finn, D. Lelievre, M. A. Lovchik, R. Poignon-Martel, G. Romey, A. Fr, P. Fr, D. Lelievre, K. Ch, M. Alan, D. Ch, *Organic Compounds*, **2013**, GB 2515128 (Givaudan).

[2] M. Vilkas, G. Senechal, *Verfahren Zur Herstellung von Hydrocitronellal*, **1969**, DE2045888 (L`Air Liquide).

[3] P. N. Davey, M. J. Earle, J. T. Hamill, S. P. Katdare, D. W. Rooney, K. R. Seddon, *Green Chem.* **2010**, *12*, 628–63.

[4] M. D. Argade, C. J. Straub, L. E. Rusali, B. D. Santarsiero, A. P. Riley, *Org. Lett.* **2021**, *23*, 7693–7697.

[5] M. Engleder, G. A. Strohmeier, H. Weber, G. Steinkellner, E. Leitner, M. Müller, D. Mink, M. Schürmann, K. Gruber, H. Pichler, *Angew. Chemie - Int. Ed.* **2019**, *58*, 7480–7484.

[6] R. M. Demming, S. C. Hammer, B. M. Nestl, S. Gergel, S. Fademrecht, J. Pleiss, B. Hauer, *Angew. Chemie* **2019**, *131*, 179–183.

[7] M. Gajdoš, J. Wagner, F. Ospina, A. Köhler, M. K. M. Engqvist, S. C. Hammer, *Angew. Chemie - Int. Ed.* **2023**, *62*, 1–6.

[8] M. Engleder, M. Horvat, A. Emmerstorfer-Augustin, T. Wriessnegger, S. Gabriel, G. Strohmeier, H. Weber, M. Müller, I. Kaluzna, D. Mink, M. Schürmann, H. Pichler, *PLoS One* **2018**, *13*, 1–19.

[9] P. Marliere, *B. Method Prod. an alkene comprising step Convert. an alcohol by an Enzym. dehydration step (Editor ed.^ eds.). City EP Pat.* **2011**, *2*.

[10] S. Steiger, A. Mazet, G. Sandmann, *Arch. Biochem. Biophys.* **2003**, *414*, 51–58.

[11] A. Hiseni, I. W. C. E. Arends, L. G. Otten, *Appl. Microbiol. Biotechnol.* **2011**, *91*, 1029–1036.

[12] A. Hiseni, L. G. Otten, I. W. C. E. Arends, *Appl. Microbiol. Biotechnol.* **2016**, *100*, 1275–1284.

[13] J. Jumper, R. Evans, A. Pritzel, T. Green, M. Figurnov, O. Ronneberger, K. Tunyasuvunakool, R. Bates, A. Žídek, A. Potapenko, A. Bridgland, C. Meyer, S. A. A. Kohl, A. J. Ballard, A. Cowie, B. Romera-Paredes, S. Nikolov, R. Jain, J. Adler, T. Back, S. Petersen, D. Reiman, E. Clancy, M. Zielinski, M. Steinegger, M. Pacholska, T. Berghammer, S. Bodenstein, D. Silver, O. Vinyals, A. W. Senior, K. Kavukcuoglu, P. Kohli, D. Hassabis, *Nature* **2021**, *596*, 583–589.

[14] G. Lozano Terol, J. Gallego-Jara, R. A. Sola Martínez, A. Martínez Vivancos, M. Cánovas Díaz, T. de Diego Puente, *Front. Microbiol.* **2021**, *12*, 1–12.

[15] A. Schütz, F. Bernhard, N. Berrow, J. F. Buyel, F. Ferreira-da-Silva, J. Haustraete, J. van den Heuvel, J. E. Hoffmann, A. de Marco, Y. Peleg, S. Suppmann, T. Unger, M. Vanhoucke, S. Witt, K. Remans, *STAR Protoc.* **2023**, *4*, 1–16.

[16] A. Schneider, P. Jegl, B. Hauer, *Angew. Chemie - Int. Ed.* **2021**, *60*, 13251–13256.

[17] M. Miyazawa, H. Nankai, H. Kameoka, *Phytochemistry* **1995**, *40*, 1133–1137.

[18] M. Miyazawa, H. Nankai, H. Kameoka, *Phytochemistry* **1996**, *43*, 105–109.

[19] M. A. Boone, R. Tong, F. E. McDonald, S. Lense, R. Cao, K. I. Hardcastle, *J. Am. Chem. Soc.* **2010**, *132*, 5300–5308.

[20] J. L. Brooks, L. Xu, O. Wiest, D. S. Tan, *J. Org. Chem.* **2017**, *82*, 57–75.

[21] Y. F. Zheng, A. C. Oehlschlager, N. H. Georgopapadakou, P. G. Hartman, P. Scheliga, *J. Am. Chem. Soc.* **1995**, *117*, 670–680.

[22] D. G. Gibson, L. Young, R. Y. Chuang, J. C. Venter, C. A. Hutchison, H. O. Smith, *Nat. Methods* **2009**, *6*, 343–345.

[23] J. Schmid, *Dissertation* **2019**, DOI http://dx.doi.org/10.18419/opus-10787.

[24] M. Kállai, Z. Veres, J. Balla, *Chromatographia* **2001**, *54*, 511–517.

[25] J. T. Scanlon, D. E. Willis, *J. Chromatogr. Sci.* **1985**, *23*, 333–340.

[26] G. C. Gruber K, Steinkellner G, *Determining Novel Enzymatic Functionalities Using Three-Dimensional Point Clouds Representing Physico Chemical Properties of Protein Cavities*, **2020**, WO2014080005A1.

[27] E. Krieger, G. Vriend, *Bioinformatics* **2014**, *30*, 2981–2982.

[28] O. Trott, A. J. Olson, *J. Comput. Chem.* **2010**, *31*, 455–461.

[29] O. Khersonsky, R. Lipsh, Z. Avizemer, Y. Ashani, M. Goldsmith, H. Leader, O. Dym, S. Rogotner, D. L. Trudeau, J. Prilusky, P. Amengual-Rigo, V. Guallar, D. S. Tawfik, S. J. Fleishman, *Mol. Cell* **2018**, *72*, 178-186.e5.

[30] S. F. Altschul, T. L. Madden, A. A. Schäffer, J. Zhang, Z. Zhang, W. Miller, D. J. Lipman, *Nucleic Acids Res.* **1997**, *25*, 3389–3402.

[31] E. W. Sayers, E. E. Bolton, J. R. Brister, K. Canese, J. Chan, D. C. Comeau, R. Connor, K. Funk, C. Kelly, S. Kim, T. Madej, A. Marchler-Bauer, C. Lanczycki, S. Lathrop, Z. Lu, F. Thibaud-Nissen, T. Murphy, L. Phan, Y. Skripchenko, T. Tse, J. Wang, R. Williams, B. W. Trawick, K. D. Pruitt, S. T. Sherry, *Nucleic Acids Res.* **2022**, *50*, D20–D26.

[32] M. A. Larkin, G. Blackshields, N. P. Brown, R. Chenna, P. A. McGettigan, H. McWilliam, F. Valentin, I. M. Wallace, A. Wilm, R. Lopez, *bioinformatics* **2007**, *23*, 2947–2948.

[33] G. E. Crooks, G. Hon, J. M. Chandonia, S. E. Brenner, *Genome Res.* **2004**, *14*, 1188–1190.
